# Polyploid frequency decreases with elevation but increases with distance to glacial refugia in the Eastern European Alps

**DOI:** 10.1038/s41467-026-77752-1

**Published:** 2026-09-12

**Authors:** Teresa Zeni, Vera Margreiter, Mar Unzeta, Tomáš Urfus, Pau Carnicero, Božo Frajman, Johannes Wessely, Petr Koutecký, Filip Kolář, Stefan Dullinger, Peter Schönswetter

**Affiliations:** 1 Department of Botany, University of Innsbruck, Innsbruck, Austria; 2 Department of Botany, Charles University, Prague, Czech Republic; 3 Department of Animal Biology, Plant Biology and Ecology, Autonomous University of Barcelona, Bellaterra, Spain; 4 Department of Botany and Biodiversity Research, University of Vienna, Vienna, Austria; 5 Department of Botany, University of South Bohemia, České Budějovice, Czech Republic

**Keywords:** Biogeography, Macroecology, Plant ecology, Plant evolution

## Abstract

Polyploidy, the possession of more than two sets of chromosomes in somatic cells, is a major driver in the diversification of flowering plants. Whereas an association between the occurrence of polyploids and latitude suggests a major impact of temperature on their global distribution, it remains unknown how polyploid frequency varies at regional spatial scales, leaving unclear the overall robustness of such relationship and its major determinants. Here, we present a flora-wide dataset of newly sampled, georeferenced ploidy occurrence data for ca. 42,000 samples of angiosperms collected along 101 elevational transects across the Eastern European Alps. Based on these data, we find that on a regional level polyploid frequency decreases with elevation, in contrast to expectations derived from global latitudinal distribution patterns. Moreover, polyploid frequency increases with distance from glacial refugia for samples below the timberline, particularly for plants with limited dispersal abilities. These results suggest that range dynamics associated with Pleistocene climatic fluctuations rather than current temperature may have played a predominant role for the current distribution of polyploids in temperate mountain ranges. In sum, our systematic fine-scale survey identified a complex interplay between historical processes and current environments on polyploid distribution that have remained undetected at the global scale.

## Abstract (web summary)

This study reveals that polyploid frequency of angiosperms decreases with elevation and increases with distance from glacial refugia in the Eastern European Alps, highlighting the role of historical range dynamics in shaping polyploid distributions.

## Introduction

Polyploidy, the possession of more than two sets of chromosomes in somatic cells and more than one set in gametes, is widely recognised as one of the major drivers in the diversification of flowering plants^1,2^. By promoting reproductive isolation, polyploidy facilitates speciation^3,4^. Polyploids might exhibit increased tolerance to biotic and abiotic stress, including drought, salinity, and temperature extremes^4–8^ as a result of direct phenotypic effects of whole-genome duplication (WGD) per se, genetic redundancy or their combination. These traits might contribute to the ecological success and invasiveness of some polyploids^5^, as well as facilitate colonisation of newly available or disturbed habitats, such as postglacial landscapes, through advantages including fixed heterozygosity and enhanced adaptive potential^2,9,10^. On the other hand, initial problems with fertility and viability, increased genetic load and weaker positive selection may confer severe disadvantages for natural polyploid mutants^9,11^. Thus, it is still unclear how these diverse and often contradictory consequences of polyploidisation translate into distributional patterns and sorting along prominent environmental gradients.

It has long been recognised that polyploids are non-randomly distributed along climatic gradients^12,13^. Early observational and floristic studies highlighted the abundance of polyploids in arctic and alpine floras and their increased frequency at higher latitudes^14,15^. Besides the aforementioned adaptive explanations, a non-adaptive alternative mechanism underlying this pattern may reflect the higher rate of production of unreduced gametes at stressful lower temperatures, resulting in higher rates of polyploid formation^16–18^. These early observations have been confirmed by a recent comprehensive meta-analysis of global chromosome count data, which showed that polyploid frequency increases strongly with latitude^19^, suggesting that climate, particularly temperature, plays a key role in shaping the global distribution of polyploid plants. However, the uncovered temperature dependence of polyploid frequency is non-trivial as temperature was suggested to act mainly indirectly, through its influence on species richness, life-form and taxonomic composition^19^ and likely also reflects the dynamic (post)glacial history of biota. In addition, the extrapolation of ploidy information derived from a few accessions over the entire species’ range, as it is commonly done in large-scale analyses, does not accurately capture the occurrence of multiple ploidies in many flowering plant species^20^.

Here, we strive to overcome some limitations of previous studies by targeting the association between polyploid frequency and temperature at a finer spatial scale and using original, systematically sampled flora-wide data. While we concur with Rice et al.^19^ in covering the entire Angiosperm flora, our sampling covers temperature variation along replicated elevation gradients equally spaced across a mountain system, the Eastern European Alps. Unlike latitudinal gradients, where potentially confounding factors such as evolutionary history, time since glaciation, and climate vary strongly, elevational gradients within one mountain range are less affected by these complexities^21–23^. Further, migration distances required for species to respond to climatic changes are much shorter in mountain regions, as species can shift along slopes to track their climatic niche^24^. Finally, and most importantly, instead of employing chromosome counts from databases, we rely on original relative genome size data from accessions systematically sampled in the field and analysed via flow cytometry of silica-dried leaf tissue^25^.

Previous studies investigating the elevational distribution of polyploids yielded mixed results. Individual studies comparing diploid and polyploid populations of the same or sister species reported cases where higher elevations were occupied either by diploids^26–30^, polyploids^31–34^ or both ploidies with no difference^35–37^. Across the British Columbian flora, polyploids—especially those of hybrid origin—were found to be disproportionately frequent at high elevations^38^. In the Swiss Alps, Favarger^39^ did not find any difference between the frequency of polyploids in the nival belt and in the surrounding lowlands and attributes their lower frequency in comparison to the Arctic region to historical rather than climatic factors. On a global scale, elevation was not a significant predictor of the frequency of polyploid species^19^. However, this may reflect limitations of models in capturing fine-scale environmental variation and steep elevational gradients^19^. In a recent meta-analysis of mixed-ploidy species, Meirmans and Kolář found a significant association of autopolyploids with lower elevation, but just in a subset of 47 species that exhibited a significant niche differentiation, while this trend was absent globally across all 129 species studied, further illustrating the complexity of such relationships and the challenges when they are addressed in comparisons across different regions, historical contexts and spatial scales^40^.

Different dispersal capabilities of polyploids may interact with the factors directly related to elevation and contribute to distinct distribution patterns. Polyploids have been suggested to be superior to diploids in terms of colonisation abilities, which may confer advantages in times of climatic turmoil, such as the Pleistocene^7^. Repeated range contractions and expansions during glacial cycles created opportunities for secondary contact, promoting hybridisation and consequently polyploid formation^41^. Consistent with this view, a suite of studies has documented a positive association between the degree of glaciation during the Pleistocene and the frequency of polyploids in different areas, including the Arctic and the Alps^15,33,42^. These results suggest that glacial dynamics promoted both polyploid origin and establishment^41,43^. Other studies, however, have yielded more complex or even contradictory results. For instance, in *Senecio carniolicus*, diploids are absent from the most heavily glaciated parts of the Alps, where hexaploids prevail, but tetraploids are strictly tied to refugia and adjacent areas^44,45^. Taken together, the often claimed association of diploids with glacial refugia is far from universally established and hasn’t been systematically tested.

**Fig. 1:**
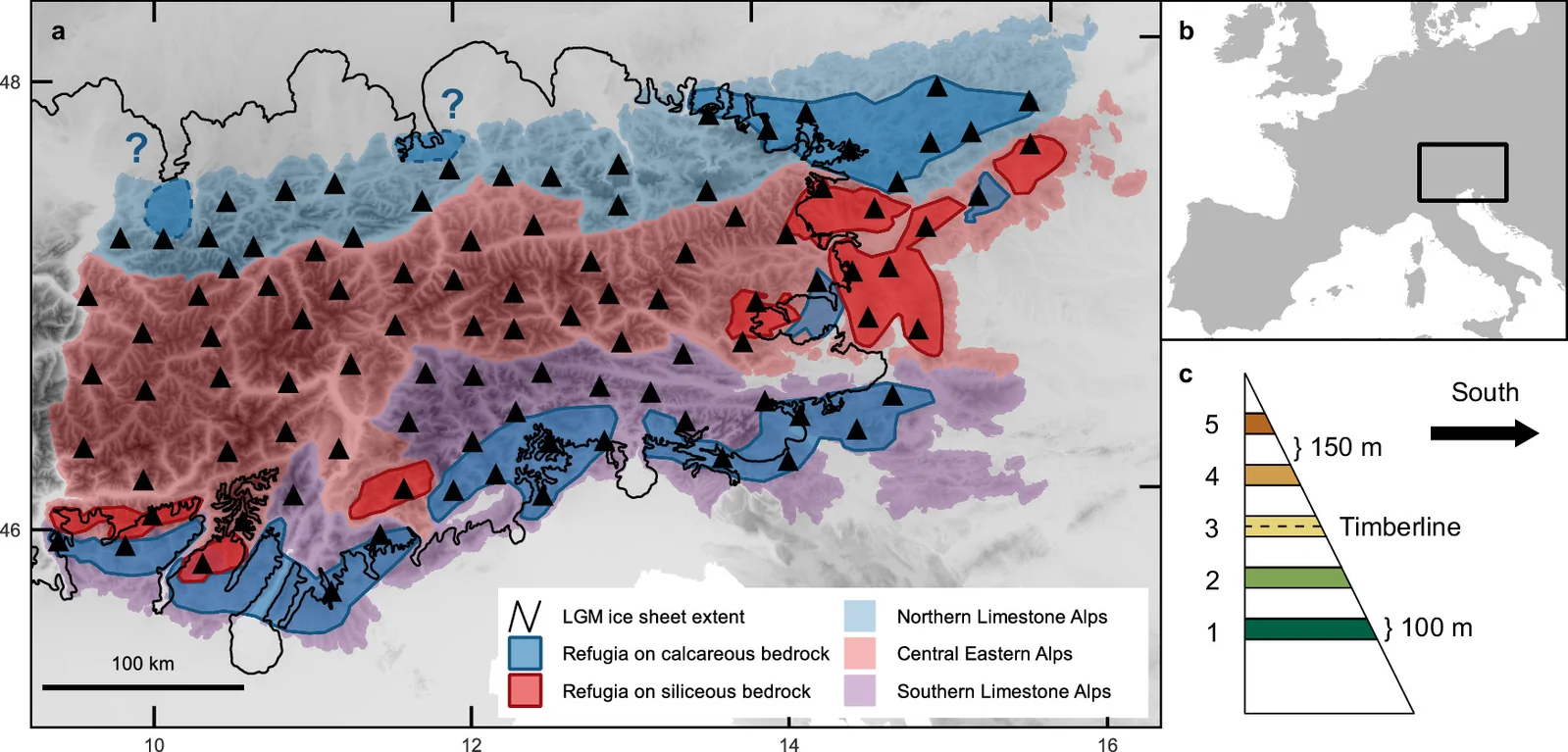
Study region and sampling design in the Eastern Alps. **a** Simplified geological map of the Eastern Alps with the 101 sampled mountain transects (black triangles), three major geographic subunits, major refugia on calcareous and siliceous bedrock (after Tribsch and Schönswetter^79^), and the extent of the Last Glacial Maximum (LGM) ice sheet (Ehlers et al.^94^, modified between Lago d’Iseo and Sava following Van Husen^95^). Limestone refugia in the western part of the Northern Limestone Alps (denoted by “?”) were not considered major refugia in this study due to their low endemism and absence of narrow-endemic taxa^79^. **b** Location of the study region within Europe. **c** Schematic representation of a sampling transect, consisting of five equally spaced belts situated on the southern slope of a mountain, centred on the timberline and spanning 1100 metres of elevation. The underlying basemap is based on the European Digital Elevation Model (EU-DEM, Eurostat GISCO)^96^ and has been modified using QGIS version 3.34.8^97^. Source data are provided as a Source Data file.

Here, we generate a flora-wide dataset of newly sampled, georeferenced ploidy occurrence dataset for ca. 42,000 samples of angiosperms collected along 101 elevational transects evenly spaced across the Eastern European Alps (Fig. 1; further details in Koutecký et al.^46^). Based on this systematic, standardised dataset, we tested two long-standing hypotheses on the contrasting distributions of di- and polyploid flowering plants. First, we hypothesised that, due to their higher tolerance to extreme environments^7,8^, polyploids are more frequent at lower temperatures, i.e., at higher elevation, reflecting the global distribution pattern of polyploids with latitude^19^. Second, we hypothesised that polyploid frequency increases with distance from glacial refugia, following the rationale that polyploids are more effective colonisers of previously glaciated areas^5,10,15^. Our results show that, in strict contrast to our first hypothesis, polyploid frequency in fact decreases with elevation. In line with our assumptions, polyploid frequency increases with distance from glacial refugia, but this effect is largely restricted to species of lower elevation belts. At higher elevations, the relationship breaks down, suggesting that elevation mediates the influence of refugial proximity on polyploid occurrence in an unexpected way.

## Results

### The dataset

We compiled a dataset of 42,229 individual plant accessions (for simplicity, “samples”) with ploidy information across 68 plant families and 1103 taxa (species or species complexes, hereafter for simplicity called “species”) (Supplementary Data 1). Altogether, we identified 210 (19.0%) species as polyploid, out of which 142 were tetraploid, and 68 were higher polyploids (hexaploids and higher). Additionally, 116 (10.5%) species were mixed-ploidy, and the remaining 777 (70.4%) were diploid. On average, we found 215 species per mountain and 85 per mountain belt, with diversity varying significantly across belts. In general, limestone mountains exhibited the strongest variation, with species richness being lowest in the upper belt (66 species in the Southern Limestone Alps and 65 in the Northern Limestone Alps) and highest in the second-lowest belt (114 and 111 species, respectively) or the timberline belt (117 and 106 species, respectively). In contrast, species richness in the Central Eastern Alps was generally lower, averaging 63 species in the upper belt and 76 at the timberline belt.

### Frequency of polyploids decreases with elevation

**Fig. 2:**
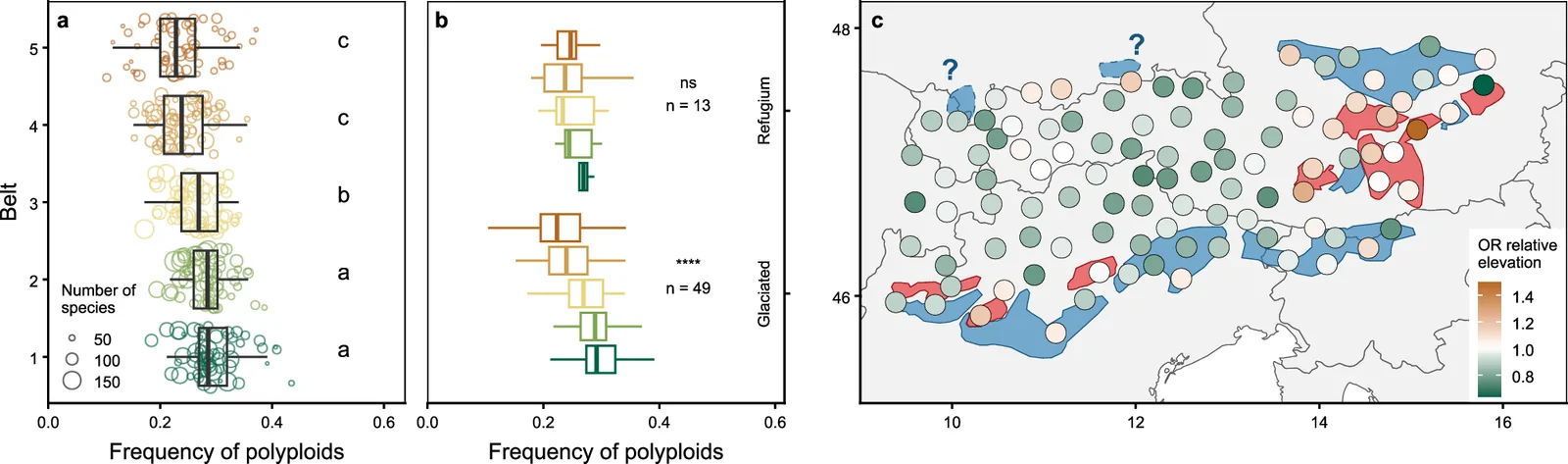
Decreasing frequency of polyploid plants with increasing elevation in the Eastern Alps. **a** Frequency of polyploids across elevation belts. Individual points depict 62 sampled mountains (for the analyses presented in **a** and **b**, 39 mountains from the Alpine periphery lacking the upper elevation belts were excluded), point size reflects the number of species collected per belt. Different letters represent significant differences among belts (based on pairwise Wilcoxon signed-rank tests, two-sided, Holm-adjusted, *P* < 0.05). Global differences across belts were tested with a two-sided Friedman test (*χ*^2^(4) = 53.21, *P* < 0.0001, *n* = 62). **b** Frequency of polyploids in regions that were glaciated during the Last Glacial Maximum and in refugial regions. Asterisks denote significance levels from two-sided Friedman tests within each group across elevational belts (**** *P* < 0.0001; ns, not significant; Glaciated: *χ*^2^(4) = 56.9, *P* < 0.0001, *n* = 49; Refugium: *χ*^2^(4) = 2.98, *P* = 0.561, *n* = 13). **c** Map with spatial distribution of odds ratios (OR) from binomial GLMs, quantifying the relationship between polyploid frequency and elevation in each of the 101 mountains; peripheral refugia on calcareous (red shading) and siliceous (blue shading) bedrock are indicated. Limestone refugia denoted by “?” were not considered due to their low endemism and absence of narrow-endemic taxa^79^. Odds ratio > 1 indicates an increasing likelihood of polyploidy with elevation, while OR < 1 indicates a decreasing likelihood. Country boundaries are from Natural Earth^98^. Box plots in panels **a** and **b** show the median (centre line) and interquartile range (IQR); whiskers extend to the most extreme values within 1.5 ⨯ IQR from the box. Exact *P*-values are available in the corresponding Source Data file. Source data are provided as a Source Data file.

We found a statistically significant difference in frequency of polyploids across elevation belts (Friedman test *χ*^2^(4) = 53.21, *P* < 0.0001, *n* = 62). Polyploid frequency was highest in the belts 1 and 2 below the timberline, intermediate at timberline (belt 3) and lowest in the subalpine and alpine belts 4 and 5. Pairwise Wilcoxon signed-rank tests revealed significant differences between elevation belts, with belt 3 showing a marked decrease relative to belts 1 and 2, and belts 4 and 5 exhibiting significantly lower frequencies than all lower belts (Fig. 2a). In a generalised linear mixed-effects model (GLMM), the effect of relative elevation was negative overall (odds ratio OR = 0.92, 95% CI = 0.90–0.94, *P* < 0.001). This negative relation (i.e., OR < 1) was strong in the majority of the mountains in interior parts of the Eastern Alps, while peripheral mountains exhibited more variation in the strength and direction of this relationship (Fig. 2c); the difference among belts was thus significant in previously glaciated mountains and not significant for mountains situated in glacial refugia (Fig. 2b). This result was qualitatively similar for a more permissive definition of polyploidy (Supplementary Fig. 1).

No trend was observed for a subset of species encompassing multiple ploidies and thus involving polyploids of presumably more recent origin than in ploidy-uniform species representing cases of completed polyploid speciation. Many polyploid lineages are thought to arise frequently but persist only transiently, implying that recently formed polyploids may not yet show consistent evolutionary or ecological patterns^47^. Across such 43 representatively sampled mixed-ploidy species (altogether 5280 samples), there was no consistent difference in elevation preference between higher- and lower-ploidy cytotypes relative to the timberline (two-sided permutation test, *P* = 0.55).

### Polyploid frequency is positively associated with distance from refugia

**Fig. 3:**
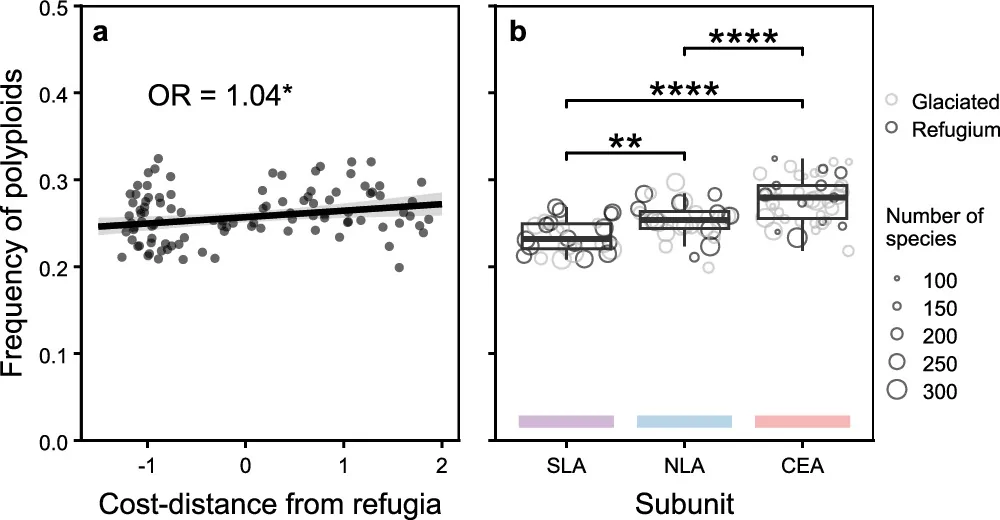
Relationship between distance from glacial refugia and frequency of polyploid plants. **a** A scatterplot shows polyploid frequency calculated for each of the 101 mountains against average cost-distance from the closest refugium with suitable bedrock. The black line represents a binomial generalised linear mixed model with 95% confidence interval (grey shading; OR = 1.04, 95% CI = 1.01–1.07, two-sided Wald test, *P* = 0.01). Cost-distance was square-root transformed and then z-standardised prior to analysis. **b** Distribution of frequency of polyploid plants across the three subunits of the Eastern Alps (SLA Southern Limestone Alps; NLA Northern Limestone Alps; CEA Central Eastern Alps). Box plots show the median (centre line) and interquartile range (IQR); whiskers extend to the most extreme values within 1.5 ⨯ IQR from the box; point size reflects the number of species collected per mountain. Differences in polyploid frequency were significant (** *P* < 0.01, **** *P* < 0.0001, two-sided pairwise t-test). Exact *P*-values are available in the corresponding Source Data file. Source data are provided as a Source Data file.

Least-cost path distances to the nearest suitable refugium were computed based on bedrock type and species’ substrate preferences, reflecting the assumption that, for instance, calcicolous species were able to migrate only through calcareous areas. Across all transects, a weak but significant positive correlation was observed between polyploid frequency and cost-distance from glacial refugia (Fig. 3a), implying that polyploids occur more frequently in areas that are further away from the closest refugium than diploids. The frequency of polyploids varied significantly across the three geographic subunits of the Eastern Alps (ANOVA, F(2, 98) = 29.52, *P* < 0.001). Mean frequency was lower in the two areas covering mainly the peripheral refugia (SLA, Southern Limestone Alps; NLA, Northern Limestone Alps) while it was significantly higher in the Central Eastern Alps (CEA), which encompasses most of the previously glaciated area (Figs. 3b and 1a).

In order to further explain the emerging patterns, we analysed species classified by their dispersal ability. The same elevational and spatial patterns as for the entire data set were revealed for poor dispersers (800 autochorous species, 30,459 samples; Supplementary Fig. 2), whereas no significant trend was found for good dispersers (303 species dispersed by water, animals or wind, 11,770 samples; Supplementary Fig. 3). However, this lack of significance coincided with high variability in polyploid frequency, particularly at high elevations, where both positive and negative trends were observed across taxa (Supplementary Fig. 4).

### Disentangling the effects of elevation and distance from refugia

**Fig. 4:**
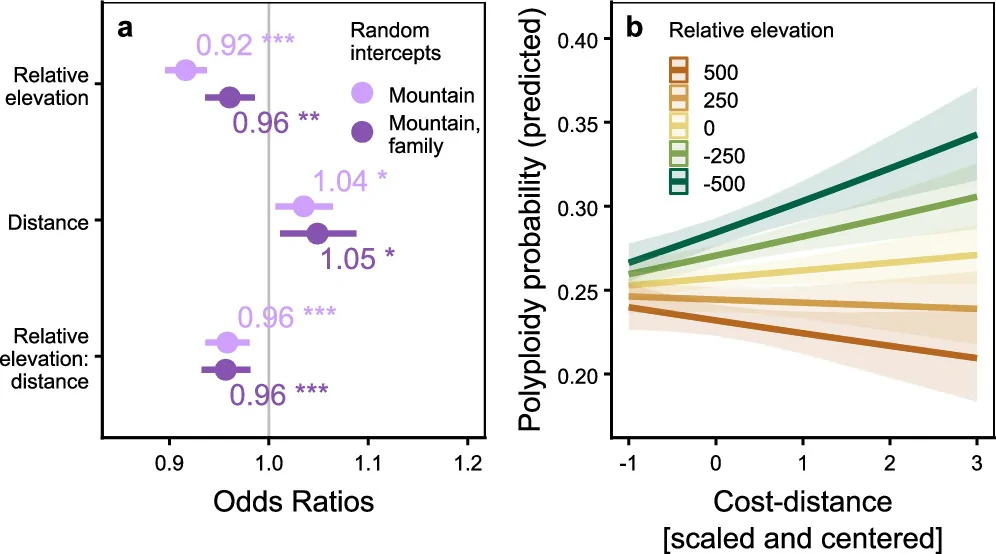
Effects of elevation and distance from refugia on the probability of a sample being polyploid. **a** Points show odds ratios with 95% confidence intervals obtained from generalised linear mixed models (GLMMs) with a binomial error distribution. Models included a random intercept for mountain to account for non-independence of samples within the same mountain and, where indicated, an additional plant family-level random intercept to account for possible taxonomic bias. Explanatory variables were z-transformed prior to analysis. Statistical significance of fixed effects was assessed using two-sided Wald tests and is indicated as * *P* < 0.05, ** *P* < 0.01, *** *P* < 0.001. **b** Predicted probability of being polyploid against cost-distance from refugia at relative elevations corresponding to the midpoints of the elevation belts. Lines show the fitted mean; shaded regions indicate 95% confidence intervals. Exact *P*-values are available in the corresponding Source Data file. Source data are provided as a Source Data file.

Generalised linear mixed-effects models (GLMMs) corroborated the significant main effects of both distance from glacial refugia and elevation relative to timberline on polyploid frequency detected in the preceding analyses. Moreover, they demonstrated a significant interaction between them (Fig. 4). The odds of polyploidy decreased by 8% for each standard deviation increase in elevation (OR = 0.92, 95% CI: 0.90–0.94) and increased by 4% for each standard deviation increase in distance OR = 1.04, 95% CI: 1.01–1.06). The interaction between elevation and distance from glacial refugia indicated a reduction of the positive distance effect on polyploid frequency at greater elevation, corresponding to a 4% reduction in odds per standard deviation increase in elevation (OR = 0.96, 95% CI: 0.94–0.98) (Fig. 4a, b). Comparison of the additive model (assuming independent effects of elevation and distance) with the interaction model (allowing the effects of elevation and distance to vary depending on each other) confirmed that including the interaction term significantly improved model fit (*χ*^2^(1) = 13.01, *P* < 0.001).

When accounting for potential effects of taxonomic composition by including plant family as an additional random intercept (GLMMs with mountain and family random effects; Fig. 4a), the overall patterns were qualitatively similar, but the effect of elevation was quantitatively reduced (OR = 0.96, 95% CI: 0.94–0.99). The effect of distance from refugia was even reinforced, with each standard deviation increase in distance now associated with a 5% increase in the odds of polyploidy (OR = 1.05, 95% CI: 1.01–1.09). The interaction between elevation and distance remained significant, indicating a weaker positive distance effect with increasing elevation (OR = 0.96, 95% CI: 0.93–0.98). Again, model comparison supported inclusion of the interaction term (*χ*^2^(1) = 11.42, *P* < 0.001). Results were consistent across GLMMs and remained robust when using alternative calculations of cost-distance and an alternative ploidy interpretation (Supplementary Data 2). All results shown here are based on a conservative ploidy interpretation, and cost-distance is calculated assuming an intermediate permeability level of unsuitable bedrock.

To test whether the elevational pattern in ploidy persists after accounting for shared ancestry, we fitted a phylogenetic logistic regression at the species level. Specifically, this model tested whether the probability of a species being polyploid varies with the mean distance of its occurrence records to the timberline, while accounting for phylogenetic relatedness. Polyploidy was negatively associated with relative elevation (β = −0.29 ± 0.13, z = −2.23, *P* = 0.03), corresponding to a 25% decrease in odds per 1 SD increase in relative elevation. Ploidy showed a weak but significant phylogenetic signal (λ = 0.17, *P* = 1.43 ⨯ 10^−5^).

Across mixed-ploidy taxa (including taxonomic species, groups of closely related species and mixed-ploidy genera), elevation and cost-distance effects on polyploidy were variable in both direction and strength and did not differ consistently between dispersal categories (Supplementary Fig. 4–8, Supplementary Data 3 and 4). While several taxa showed significant relationships with elevation and cost-distance, the direction of this effect was inconsistent and overall group-level patterns were not significantly different from no effect. In addition, the spatial distribution of cytotypes within mixed-ploidy species also varied. In some species, cytotypes were restricted to former peripheral refugia (Supplementary Fig. 9a, b), while in others, one cytotype occurred in the western part and the other in the eastern part (Supplementary Fig. 9c, d).

**Fig. 5:**
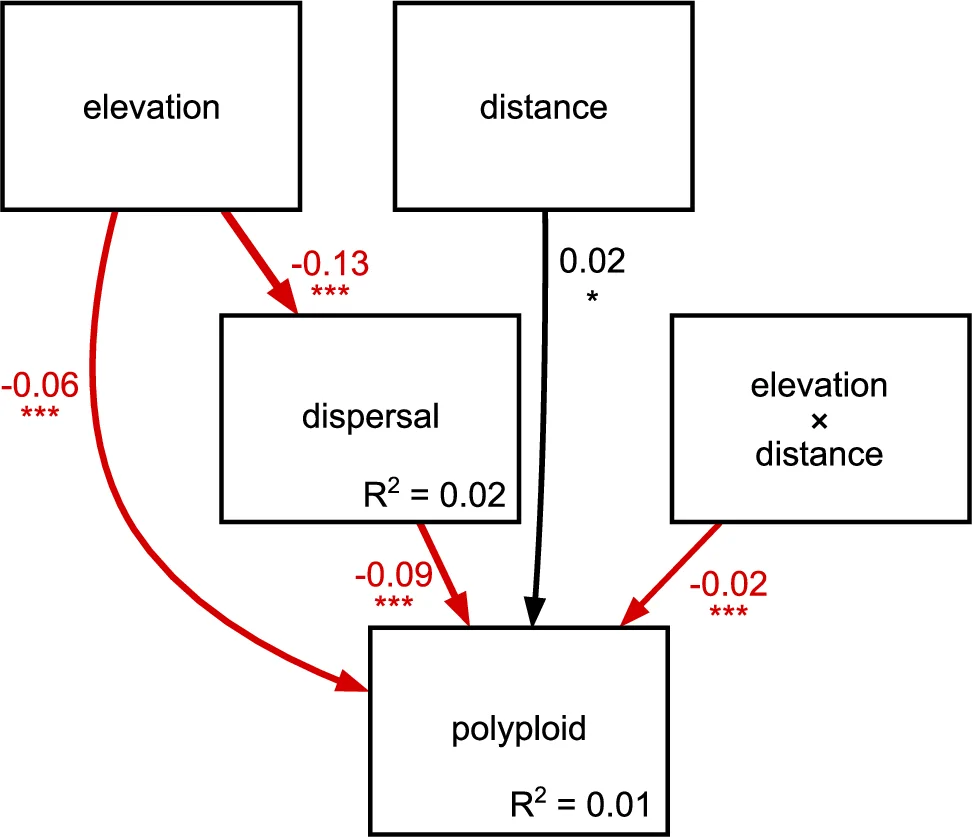
Piecewise structural equation model linking elevation, distance from glacial refugia, dispersal ability, and polyploidy. The model tests direct and indirect effects of relative elevation to timberline (elevation) and cost-distance from glacial refugia (distance) on the probability that a sample is polyploid, mediated by dispersal ability (dispersal: good vs. poor disperser). Boxes represent variables, arrows indicate directional relationships, and numbers next to arrows are standardised path coefficients. Asterisks indicate statistical significance of the path coefficients based on two-sided Wald tests (* *P* < 0.05, *** *P* < 0.001). Global fit was assessed using a test of directed separation (right-tailed *χ*^2^ test): Fisher’s C(2) = 3.199, *P* = 0.202. *R*^2^ values shown are marginal *R*^2^ computed with the theoretical variance method for binomial models. Exact *P*-values are available in the corresponding Source Data file. Source data are provided as a Source Data file.

To disentangle direct effects from indirect relationships among correlated predictors, we used piecewise structural equation models^48^ to evaluate how elevation, distance from refugia and dispersal ability interact to shape the observed pattern in the probability of a sample being polyploid. Good dispersers were less likely to be polyploid. Elevation influenced the probability of being polyploid indirectly through its effect on dispersal strategy. Distance showed weaker indirect effects, as its relationships with dispersal ability were not statistically significant, but had a significant direct effect on polyploid probability (Fig. 5).

## Discussion

Based on a large flora-wide dataset, our analyses uncovered how the spatial distribution of polyploids relates to elevation and distance from glacial refugia, revisiting old hypotheses that were so far addressed in isolated case studies and by verbal summaries. First, and in contrast to our expectations, the analysis of a systematically sampled dataset from 101 elevational transects spanning the entire Eastern European Alps detected a statistically significant negative effect of elevation on the frequency of polyploids. Second, we show that polyploid frequency increases with distance from glacial refugia; this pattern is strongest at lower elevations, while at higher elevations the positive effect of distance vanishes. By focusing on fine-scale data, we capture patterns that broader-scale studies may overlook and mitigate the influence of potential confounding factors, such as floristic composition or climate, which can dominate at larger geographical scales.

Contrary to our initial hypothesis, we observed a decrease in polyploid frequency with increasing elevation (Fig. 2a), also when potential interactions with geography and taxonomic biases in floristic composition were considered (Fig. 4a). This result is largely in line with a recent world-wide compilation targeting mixed-ploidy taxa^40^ and probably suggests polyploidy does not provide any universal adaptive solutions to low-temperature and alpine stress and/or that UV-induced formation of polyploids^49^ has at most a limited effect on the frequency of established polyploids above the timberline. Instead, several other ecological and physiological mechanisms may explain the lower frequency of polyploids at higher elevations. Specifically, (neo-)polyploids exhibit reduced growth rates and longer meiotic and mitotic cycles in comparison to related diploids^50,51^, which may be disadvantageous in environments with short growing seasons^52^. Many polyploids are taller^45,53^ and require a longer time than diploids for reproduction^54^, which can limit reproductive success at high elevations. However, these patterns are not consistent across taxa. In some taxa, polyploids flower earlier than diploids^55,56^, and size differences between cytotypes are likewise variable^55^. Further, polyploids might also experience limited freezing tolerance due to increased vulnerability to cavitation in their larger vessel elements^57^. However, we highlight that the listed challenges are shared with high-latitude habitats. Finally, previous studies suggested that polyploids better thrive in complex, competitive habitats^58^, which are more common at lower elevations^59,60^.

Differences in taxonomic composition across elevations probably contribute to the observed pattern, as, hypothetically, polyploid-rich taxa might be disproportionately represented in low-elevation communities such as forests, following the variation in polyploid frequency among families^47^. To assess whether this pattern reflects taxonomic non-independence, we conducted two complementary analyses: a GLMM with family as a random intercept and a phylogenetic logistic regression that accounts for shared ancestry among taxa. Indeed, when accounting for taxonomic composition by using family as a random intercept in the GLMMs, the negative effect of elevation was reduced. That it remained significant (Fig. 4a), however, suggests that the decrease in frequency of polyploids with elevation is not solely driven by the differing taxonomic composition of high- and low-elevation communities. Consistent with these results, the phylogenetic model also supports a negative relationship between polyploidy and elevation, indicating that this pattern is robust to alternative ways of accounting for non-independence among taxa.

As a second major trend, we found a consistent increase in polyploid frequency with increasing distance from glacial refugia across the entire data set (Fig. 3a), pointing to an important role of postglacial colonisation dynamics. Several mechanisms might lead to polyploids accumulating at greater distances from refugia. Among “arctic specialist” taxa, diploids are more frequent in refugia^15^, consistent with the view that polyploids are more successful colonisers following deglaciation. This success is likely due to their ability to buffer colonisation-related bottlenecks and inbreeding^61,62^. For instance, autopolyploids at leading edges were shown to maintain genetic diversity during range expansion^10^; similar mechanisms were suggested to explain the frequent occurrence of high levels of allopolyploidy in arctic archipelagos^15^. In contrast, when focusing on the mixed-ploidy species in our dataset, the lack of consistent cost-distance effects rejects the hypothesis that secondary contact between lineages that diverged in separate refugia promoted the formation of polyploids^41^ in the Eastern European Alps. In the same line, the inconsistent distribution of cytotypes in mixed-ploidy species—with recently emerged polyploids sometimes restricted to refugia and sometimes exclusively occurring outside of them (Supplementary Fig. 9)—does not support the hypothesis that newly deglaciated, sparsely vegetated terrain uniformly facilitates both establishment and early persistence of new polyploid cytotypes by reducing minority cytotype exclusion^63,64^.

Polyploid frequency varied significantly among the three major subunits of the Eastern European Alps, likely reflecting, amongst others, the combined effects of different substrate-associated species pools and the configuration of glacial refugia. The Southern Limestone Alps, of which large parts have acted as, or were located close to, refugia, harbour lower polyploid frequencies than the Northern Limestone Alps and the Central Eastern Alps (Figs. 1a and 3b), which were more strongly glaciated. This pattern is consistent with refugial persistence of diploid lineages and postglacial expansion of polyploids into newly deglaciated terrain^33^. In addition, different bedrock is known to confer differences in nutrient availability. In particular, soils on calcareous bedrock typically exhibit lower availability of plant-accessible phosphorus than soils on siliceous bedrock^65,66^. Low nutrient availability has been suggested to restrict the abundance of polyploid plants due to the increased nutrient demands associated with larger (duplicated) genomes^67,68^, and this hypothesis was confirmed in grassland fertilisation experiments^69,70^. Finally, we cannot exclude that differences in taxonomic composition of the angiosperm floras across the three regions and the two substrate types contribute to the observed spatial variation in polyploid frequency.

The effect of distance from refugia on polyploid frequency was elevation-dependent as shown by the significant interaction of elevation and distance in the GLMMs (Fig. 4). Focussing on the elevational trend separately in formerly glaciated areas versus major refugia, we found that transects located within refugia—even those lacking direct phylogeographic support (indicated by question marks in Figs. 1a and 2c)—showed no clear elevational trend, while previously glaciated regions exhibited a significant decrease in polyploid frequency with elevation (Fig. 2b, c). This, again, points at a stronger sorting of established polyploids vs. diploids in the course of postglacial colonisation dynamics—but not necessarily in recently emerged polyploids in mixed-ploidy species—and underlines the overall superiority of polyploids in times of climatic turmoil.

Notably, both elevational and distance trends were absent in species dispersed by wind, water or birds (Supplementary Fig. 3), suggesting that the dispersal mode modifies the relationship between ploidy and elevational gradient. This is likely because efficient migrators maintained higher connectivity among populations via seed dispersal during glacial cycles and colonised deglaciated terrain more rapidly, probably preventing the establishment of newly emerged polyploids on the leading edge of a species’ distribution^71,72^. Although there is no direct, mechanistic link between WGD and long-distance dispersal, successful long-distance dispersers have been reported to be more likely to be polyploid in grasses^73^. Consequently, polyploidy may buffer these species against founder events and phases of small population size, mitigating the demographic and spatial constraints of high elevations and likely reducing the influence of postglacial colonisation history. However, the large variation in polyploid frequency observed, especially above the timberline, indicates this pattern is not consistent across taxa. Such variability might arise from heterogeneity in dispersal strategies and life-history traits within this group of species with good dispersal ability.

Separate analyses of mixed-ploidy taxa (species and groups of closely related species) offer additional insights into the origins of the observed broad-scale patterns. Mixed-ploidy taxa represent recent or ongoing polyploidisation events, capturing “snapshots” of WGD early in its evolutionary trajectory^71^. Importantly, mixed-ploidy taxa showed no consistent patterns, neither in terms of the strength nor the direction of the effect, indicating that they did not drive the elevational or distance trends in overall polyploid frequency in our data (Supplementary Figs. 4–7). This suggests that the flora-wide patterns are unlikely to reflect ecological traits directly connected to recent WGD. At this early stage, ecological responses might be noisy and context-specific^40^. By contrast, lineages with uniform ploidy level reflect older, stabilised WGD events in which polyploid cytotypes have undergone divergence, long-term selection, and/or gradual genome evolution towards diploidy (e.g., subfunctionalisation) over longer time scales^11^. While previous comparative studies found frequent differences in climatic niches between diploid and polyploid lineages in mixed-ploidy taxa^6,40^, this trend was absent in some other studies^74–76^. Most importantly, the direction of the difference was not consistent, suggesting there is no general parameter consistently separating polyploids from their lower-ploidy progenitors^40^. In line with this notion, our results support the emerging view that recent WGD does not produce consistent or predictable ecological outcomes across species.

Our study bridges the gap between many single-species case studies, which yielded contradictory results on the one hand, and global studies addressing species-level patterns at coarser scales^19^ on the other hand. We show that the distribution of polyploidy is non-random at landscape scales and that there is significant interaction between sorting along steep environmental gradients and a dynamic Pleistocene history. Specifically, elevation contributed to polyploid probability largely indirectly through its effects on traits (Fig. 5), whereas distance from refugia exhibited a direct effect, suggesting that historical processes associated with postglacial colonisation may play a prominent role in shaping current polyploid distribution. Our results clearly show that on a regional level, ploidy level does not increase with elevation, challenging at least the generality of the notion of the positive association of polyploidy with harsh and unpredictable environments^7^. Overall, our results suggest that the relationships between polyploidy and elevation are not an intrinsic ecological or physiological consequence of WGD that would act as a constraint or provide adaptive benefit at particular elevations, but largely reflect species’ idiosyncrasies as well as differences in taxonomic composition, ecological strategies, and historical range dynamics of the species occupying different elevational belts.

## Methods

### Sampling and ploidy estimation

Appropriate permits for sample collection were obtained from the relevant local authorities prior to fieldwork. Details of the issuing authorities and permits are provided in Supplementary Table 1. Samples were collected along 101 elevational transects evenly spaced across the Eastern Alps (Switzerland, Germany, Austria, Italy, Slovenia; Fig. 1a, b and Supplementary Data 1). We strived to position transects on southern slopes, which was not possible in all cases. However, northern expositions were strictly avoided. Each transect covered 1100 m in elevation difference and was centred on the timberline (Fig. 1c).

Timberline position was identified using Google Earth by selecting the highest-elevation occurrences of tree groups within a 5 km radius of each summit (or a nearby summit if image quality was poor). This approach aims to counterbalance the anthropogenic lowering of the timberline by including areas likely to be unaffected. Transects comprised five equally spaced belts spanning 100 m in elevational difference, separated by non-sampled intermittent 150 m in elevation. In 39 transects, the highest belt (5) was absent due to insufficient summit height; in 9 transects, the second-to-highest belt (4) was lacking. In these cases, we sampled the uppermost 100 m of elevation difference of the north-exposed summit slope (hereafter referred to as belt N), in order to cover the local species pool as completely as possible by including also the most cold-adapted species that usually occupy north-exposed slopes. Overall, the sampled elevation range spanned between 972 and 2971 m. In each belt, we sampled all native (i.e., non-neophytic) angiosperms from all types of natural or semi-natural vegetation except for aquatic macrophytes; clearly anthropogenic habitats such as ruderal and segetal places as well as strongly altered secondary grasslands were avoided. Species from Annex II of the Habitats Directive (in our case *Campanula zoysii* and *Cypripedium calceolus*) were not collected, as well as the taxonomically highly intricate, predominantly apomictic genera *Alchemilla*, *Hieracium*, *Pilosella*, and *Taraxacum*, as well as *Rubus* sect. *Rubus* and the *Rosa canina* aggregate, which could not be exhaustively sampled without the permanent presence of a specialist.

Silica-dried plant material from each accession was measured by flow cytometry, following protocols detailed in Koutecký et al^46^. This approach yields a raw estimate of relative genome size for each sample, which was then used to infer each individual’s ploidy cytotype. From an initial dataset of 43,745 samples, we excluded 885 samples from 63 species due to unreliable flow cytometric histograms, and 498 samples from 4 species (*Poa alpina*, *P. angustifolia*, *P. nemoralis*, *P. pratensis*), for which no discrete ploidy cytotypes could be differentiated due to pervasive aneuploidy (known in *Poa*^77,78^). Combining published chromosome data and measured relative genome size values, we used a dual approach for the coding of ploidy. In the first approach, biased towards diploids, we assigned diploid status to the lowest reliable European sporophytic chromosome count within each genus; a few, usually old, counts with unusual base chromosome numbers were not taken into account. In large and karyologically diverse genera (i.e., different base chromosome numbers, due to rearrangements in distantly related species), infrageneric entities such as sections, subsections, or series were treated as base units, and the lowest chromosome count within each entity was considered diploid. In the second approach, biased towards polyploidy, diploidy was defined only at the genus level using both European and reliable extra-European counts, i.e., infrageneric entities were not taken into account. Since we focused on recent polyploids rather than ancient genome duplications where diploidisation has already progressed, we present the results obtained based on the first coding approach in the main manuscript, while those of the second coding approach are shown in the Supplementary Information. Cytotypes were excluded from all analyses if they were both odd-ploid (e.g., triploids, pentaploids) and rare; the latter was defined as comprising less than 5% of occurrences within a species, or representing a single sample in species with 20 or fewer samples, which accounted for 128 samples. For 340 samples with missing ploidy data due to failed flow cytometric measurements, but belonging to species with uniform ploidy, ploidy was assigned based on the successfully measured samples of the species.

### Cost-distance calculation

Using least-cost path analysis, we calculated the cost-distance from each transect to the nearest refugium on silicate and carbonate substrates by modelling movement across the landscape based on geology-resistance values. Major refugia on limestone and siliceous bedrock (Fig. 1) were based on Tribsch and Schönswetter^79^; limestone refugia in the western part of the Northern Limestone Alps were not included, as they were not considered major refugia due to their low endemism and absence of narrow-endemic taxa^79^. The conductance rasters, representing the inverse of movement difficulty, were first generated by rasterising a geo-lithological map for Central Europe (Geo-LiM^80^) using a reference raster with 100 m resolution. Three versions of the conductance rasters (vA, v0, vB) were generated, each representing increasing (assumed) permeability levels of the unsuitable bedrock categories. These rasters were then aggregated to 200 m resolution by averaging values over 2 × 2 pixel blocks to enhance computational efficiency while preserving spatial patterns. Conductance values were assigned based on a conversion table that links geological properties to movement difficulty for three plant categories: acidophilous (preference for siliceous bedrock); basiphilous (preference for calcareous bedrock); and intermediate-preference species, which can occur on both substrates (Supplementary Table 2). Plant species were classified based on ecological indicator values for the Austrian flora^81^. For species restricted to Italy and Slovenia, values were derived from Pignatti et al^82^. Categories were defined as follows: acidophilous = [1–3], intermediate = [4–6], basiphilous = [7–9]. Separate conductance rasters were created for each category, reflecting their specific movement constraints across the landscape. For each category, the conductance raster was then converted into a transition matrix via an 8-direction connectivity model using a mean function to determine movement costs between connected cells using the transition function in the package gdistance^83^ version 1.6.4. Least-cost paths were then computed from each transect to the nearest refugium of the appropriate geological type using the costDistance function in the package gdistance^83^. Cost-distances for acidophilous plants were calculated to refugia located on siliceous bedrock, while basiphilous plants were linked to refugia on limestone. For intermediate-preference species, cost distances were calculated to the nearest refugium, regardless of bedrock type. A correction was then applied by setting the cost-distance to zero for transects already located within refugia.

### Statistical analyses

Polyploid frequency was calculated at the elevation-belt level as the number of polyploids divided by the number of samples for which the ploidy level was determined within each belt. Differences in polyploid frequency among elevation belts and geographic subunits of the Eastern Alps were assessed using Friedman tests (for elevation belts, with mountain as blocking factor) and ANOVA (for geographic subunits), followed by pairwise Wilcoxon signed-rank tests and t-tests, respectively, to identify differences between specific belts or subunits. *P*-values were adjusted for multiple comparisons using Holm-Bonferroni correction^84^. The relationship between mountain-level polyploid frequency and average cost-distance from the nearest glacial refugium was assessed using a binomial generalised linear model (GLM), implemented with the glm function in the stats package (version 4.4.1)^85^. Polyploid frequency on each mountain was modelled as the number of polyploid cytotypes divided by the total number of cytotypes recorded on that mountain, counting each cytotype only once. Prior to analysis, cost-distance values were square-root transformed and z-standardised.

To assess whether dispersal ability influenced the observed patterns, we repeated the analyses for good and poor dispersers. Taxa were assigned to categorical groups based on dispersal ability^86^. Transect-level polyploidy was further analysed using binomial GLMs and generalised linear mixed-effects models (GLMMs), implemented with the glmer function in the lme4 package (version 1.1-35.5)^87^, with polyploid samples treated as “successes” and diploid samples as “failures”. Relative elevation to timberline was included as a numeric predictor, scaled to one standard deviation and centred on the mean. Estimated fixed-effects were exponentiated to obtain odds ratios, representing the multiplicative change in the odds of polyploidy per one standard deviation increase in elevation. Transect-specific models were fitted for each mountain, and a global GLMM across all transects, including a random intercept for mountain identity, was used to describe the general trend.

We additionally tested whether higher-ploidy cytotypes within mixed-ploidy taxa (mixed-ploidy species and aggregates according to Schratt-Ehrendorfer et al.^88^) occur at higher elevations in comparison to lower-ploidy cytotypes. Cytotypes with fewer than ten occurrences were excluded from the analysis. For taxa with more than two cytotypes, only the two ploidy levels with the highest number of occurrences were retained. For each cytotype in each taxon, mean elevation relative to the local timberline was calculated, and taxon-level elevation differences were defined as the difference in mean elevation between higher- and lower-ploidy cytotypes. To assess significance, we performed 10,000 within-taxon permutations: in each iteration, cytotype labels were randomised within taxon and taxon-level mean elevation differences recalculated. The observed difference was compared to the distribution of the permuted differences, and a two-sided *P*-value was determined as the proportion of permuted differences with absolute values greater than or equal to the absolute observed difference.

The effect of distance from glacial refugia and elevation, and their interaction on the distribution of ploidy levels was also examined using binomial GLMMs with a logit link function. This was done to address potential limitations of the Friedman test, which could not include all mountains due to missing upper elevation belts in some transects. In this model, polyploid samples were treated as “successes” and diploid samples as “failures”. Elevation of each sample was recalculated relative to the local timberline by subtracting the timberline elevation from the observed elevation. Numerical predictors (elevation and distance from the closest refugium) were scaled to one standard deviation and centred on the mean, allowing for easier interpretation of effect sizes. To account for non-independence among samples from the same mountain, the mountain was included as a random intercept. Each predictor was initially tested independently, followed by the fitting of an additive model, including both elevation and distance as main effects. Finally, a model with interaction between elevation and distance was fitted to explore whether the relationship between these predictors and ploidy levels depended on one another. To compare the fit of the additive model and the interaction model, a likelihood ratio test was performed between the two nested models (elevation + distance vs. elevation * distance). To account for a possible effect of changes in taxonomic composition with elevation on the likelihood of sampling a polyploid, family was also included as an additional random intercept in a second set of models using the same fixed-effects structure. This approach allowed for variability in the likelihood of being polyploid across families, since families are not distributed homogeneously along the elevational gradient, and the frequency of polyploidy varies across families^89^. Two hundred fifty-eight samples from five species that could not be assigned to a bedrock-preference category—required to calculate cost-distance from glacial refugia—were excluded from the models testing the role of distance from glacial refugia. For the models with elevation, only samples collected in belts 1–5 were included (39,720 samples), implying that samples from belt N were excluded, as they lie outside the continuous elevational gradient relative to the timberline.

As an alternative to accounting for taxonomic non-independence using family-level random effects, we performed a complementary phylogenetic analysis that explicitly incorporates shared ancestry among species. We used a pruned phylogeny of European arctic-alpine vascular plants^90^, including only species with a direct match in the tree and at least ten samples in our database (*n* = 589). Phylogenetic signal in species ploidy (calculated as the proportion of polypoid samples per species) was quantified using Pagel’s λ^91^, estimated by maximum likelihood and tested against λ = 0 with a likelihood-ratio test, as implemented in the phytools package (version 2.4.4)^92^. To assess whether elevation predicts the probability of a species being polyploid while accounting for phylogenetic non-independence, we fitted a phylogenetic logistic regression using the function phyloglm from the R package phylolm (version 2.6.5)^93^. Ploidy was coded as a binary species-level trait based on a majority rule ( > 50% polyploid samples). Distance to timberline was scaled to one standard deviation and centred on the mean; species-level predictor values were obtained by averaging these standardised distances across samples within each species.

To assess whether associations between polyploidy and elevation or cost-distance were consistent across taxa, we conducted taxon-specific binomial GLMs using mixed-ploidy taxonomic species, groups of closely related species, and mixed-ploidy genera. Ploidy was treated as a binary response (polyploid vs. diploid), relative elevation to timberline and cost-distance from glacial refugia were included as fixed effects. Models were fitted for each taxon with sufficient data (at least ten samples per response category). To test whether dispersal ability was associated with taxon-level elevation effects on ploidy, we compared odds ratios across groups. Group-level differences in elevation and distance effects were assessed by comparing the distribution of taxon-level odds ratios.

We tested direct and indirect relationships among elevation, cost-distance from glacial refugia, dispersal ability, and the probability of a sample being polyploid using piecewise structural equation modelling (piecewise SEM), implemented in the package piecewiseSEM (version 2.3.0)^48^. Piecewise SEM integrates multiple GLMMs, accounting for non-normal responses and hierarchical data^48^. All component models were fitted as binomial GLMMs with a logit link and a random intercept for transect identity to account for non-independence among samples from the same mountain. Dispersal ability (1 = good, 0 = poor) was modelled as a function of elevation. Polyploidy status (1 = polyploid, 0 = diploid) was modelled as a function of elevation, distance, and dispersal ability, including an interaction term between elevation and distance. Standardised path coefficients were used to compare effect sizes.

All analyses were performed in R (version 4.4.1)^85^.

### Reporting summary

Further information on research design is available in the Nature Portfolio Reporting Summary linked to this article.

## Supplementary information

Supplementary Information

Description of Additional Supplementary Files

Supplementary Data 1

Supplementary Data 2

Supplementary Data 3

Supplementary Data 4

Reporting Summary

Transparent Peer Review file

## Source data

Source Data

## Data Availability

The complete dataset used for all analyses presented in this study is provided in Supplementary Data 1. The raw flow cytometry files are not included in this study. The flow cytometry methodology is described in detail in Koutecký et al^46^. The relative genome size values and corresponding reference standards are provided in Supplementary Data 1. The raw flow cytometry data are available upon request from the corresponding author. Source data are provided with this paper.

## Code availability

The R scripts used for the analyses presented in this study are publicly available on GitHub at https://github.com/terezeni/polyalp_distribution_patterns and are archived at Zenodo (https://doi.org/10.5281/zenodo.21701400). The archived version used for this study is version 1.0.0. The code is released under the GNU General Public License v3.0 (GPL-3.0).
