# Supplementary material for: Polyploid frequency decreases with elevation but increases with distance to glacial refugia in the Eastern European Alps
Source: Nat Commun. 2026 Sep 12;17:10768. doi: 10.1038/s41467-026-77752-1 (PMC13661597; doi:10.1038/s41467-026-77752-1)
Supplement: Supplementary file 1 [file 41467_2026_77752_MOESM1_ESM.pdf]

Supplementary Information for: Polyploid frequency decreases with elevation but increases with distance to glacial refugia in the Eastern European Alps

Supplementary Tables 2
Supplementary Figures 5
Supplementary References 32

Supplementary Tables

Supplementary Table 1. Permits for sample collection.

Supplementary Table 2. Conversion table for cost distance-calculation.

Supplementary Table 1. Permits obtained for sample collection. Issuing authorities and corresponding permit or protocol numbers.

Issuing authority	Protocol number
Autonome Provinz Bozen – Amt für Natur	599690 p_bz (15.7.2022), 534663 p_bz (12.7.2021)
Kanton Graubünden – Amt für Natur und Umwelt	AV-2022-151 (3.3.2022)
Bezirkshauptmannschaft Bregenz	BHBR-I-7100.00-20/2022-16
Bezirkshauptmannschaft Feldkirch	BHFK-II-6101-9/2022-8
Bezirkshauptmannschaft Feldkirchen	FE3-NS-3275/2022 (07/2022)
Bezirkshauptmannschaft Kirchdorf	BHKIN-2022-426748/9-Eb
Bezirkshauptmannschaft Lienz	LZ-NSCH-3/1289-2022
Bezirkshauptmannschaft Spittal an der Drau	SP3-NS-3939/2022 (004/2022)
Bezirkshauptmannschaft St. Veit an der Glan	SV19-ALL-1582/2022 (005/2022)
Bezirkshauptmannschaft Villach-Land	VL3-NS-3914/2022 (005/2022)
Bezirkshauptmannschaft Wolfsberg	WO3-NS-3296/2022 (006/2022)
Comunità della Val di Non	304/SEG dd. 10 06 2021
Comunità della Vallagarina	C10/C10-SAM-0016284-PROT-01/07/2021-P
Comunità delle Giudicarie	006855/11.3 (9/6/2021)
Comunità Valle di Sole	C7-0003565-19/05/2022-11:13 P
Comunità Valsugana e Tesino	cvt_tn-10/06/2021-0007757-P
Republika Slovenija – Ministrstvo za okolje in prostor	35606-61/2022-2550-4
Amt der Niederösterreichischen Landesregierung – Abteilung Naturschutz	RU5-BE-1843/001-2022
Parco Naturale Adamello Brenta	pnab_tn-04/06/2021-0002356
Parco Nazionale dello Stelvio - Lombardia	ERSAF.2021.0007342
Parco Nazionale Dolomiti Bellunesi	74(14-7-2021)
Provincia Autonoma di Trento – Servizio Sviluppo Sostenibile e Aree Protette	PAT/RFS175-13/07/2021-0504988
Regierung von Oberbayern	ROB-55.1-8645.NAT_02-25-9-4
Regione Lombardia – Direzione Generale Ambiente e Clima	T1.2021.0060540
Amt der Salzburger Landesregierung – Abteilung 5 Natur- und Umweltschutz, Gewerbe	205-05RI/1208/8-2022
Regione Autonoma Friuli Venezia Giulia – Direzione centrale risorse agroalimentari, forestali e ittiche	0040791/P, 0037054/P
Amt der Steiermärkischen Landesregierung – Abteilung 13 Umwelt und Raumordnung	ABT13-348560/2022-6
Comun General de Fascia	2560-11.3
Regione Veneto – Unità Organizzativa Servizi Forestali, Belluno	261033 (9/6/2021), 261035 (9/6/2021), 198511 (3/5/2022), 197574 (2/5/2022), 197591 (2/5/2022), 197558 (2/5/2022), 198548 (3/5/2022)
Regione Veneto – Unità Organizzativa Servizi Forestali, Vicenza	256267 (7/6/2021)

Supplementary Table 2. Conversion table for cost-distance calculation. Values to convert lithological map Geo-LiM[1] to conductance for three different categories of species based on substrate preference. S: acidophilous; K: basiphilous; I: intermediate. Versions vA, v0, vB represent increasing (assumed) permeability levels of the unsuitable bedrock categories.

	vA			v0			vB		
category in Geo-LiM	S	K	I	S	K	I	S	K	I
acid rocks	1	0.05	0.05	1	0.1	0.1	1	0.2	0.2
claystone	1	0.05	0.05	1	0.1	0.1	1	0.2	0.2
mafic rocks	1	0.5	1	1	0.5	1	1	0.5	1
sandstone	1	0.05	0.05	1	0.1	0.1	1	0.2	0.2
intermediate rocks	1	0.5	1	1	0.5	1	1	0.5	1
gypsum evaporite	0.05	1	1	0.1	1	1	0.2	1	1
mix carbonate rocks	0.05	1	1	0.1	1	1	0.2	1	1
pure carbonate rocks	0.05	1	1	0.1	1	1	0.2	1	1
metamorphic rocks	0.5	0.5	1	0.5	0.5	1	0.5	0.5	1

Supplementary Figures

Supplementary Figure 1. Distribution of frequency of polyploid plants across elevation belts in the Eastern Alps using a more permissive definition of polyploidy.

Supplementary Figure 2. Elevational patterns and refugial-distance effects on polyploid frequency in autochorous plant species in the Eastern Alps.

Supplementary Figure 3. Elevational patterns and refugial-distance effects on polyploid frequency in anemo-, hydro-, and zoochorous plant species in the Eastern Alps.

Supplementary Figure 4. Genus-specific model predictions from binomial GLMs (elevation).

Supplementary Figure 5. Species-specific model predictions from binomial GLMs (elevation).

Supplementary Figure 6. Genus-specific model predictions from binomial GLMs (distance).

Supplementary Figure 7. Species-specific model predictions from binomial GLMs (distance).

Supplementary Figure 8. Species-level elevation and distance effects on polyploidy.

Supplementary Figure 9. Geographic distribution of cytotypes in mixed-ploidy species.

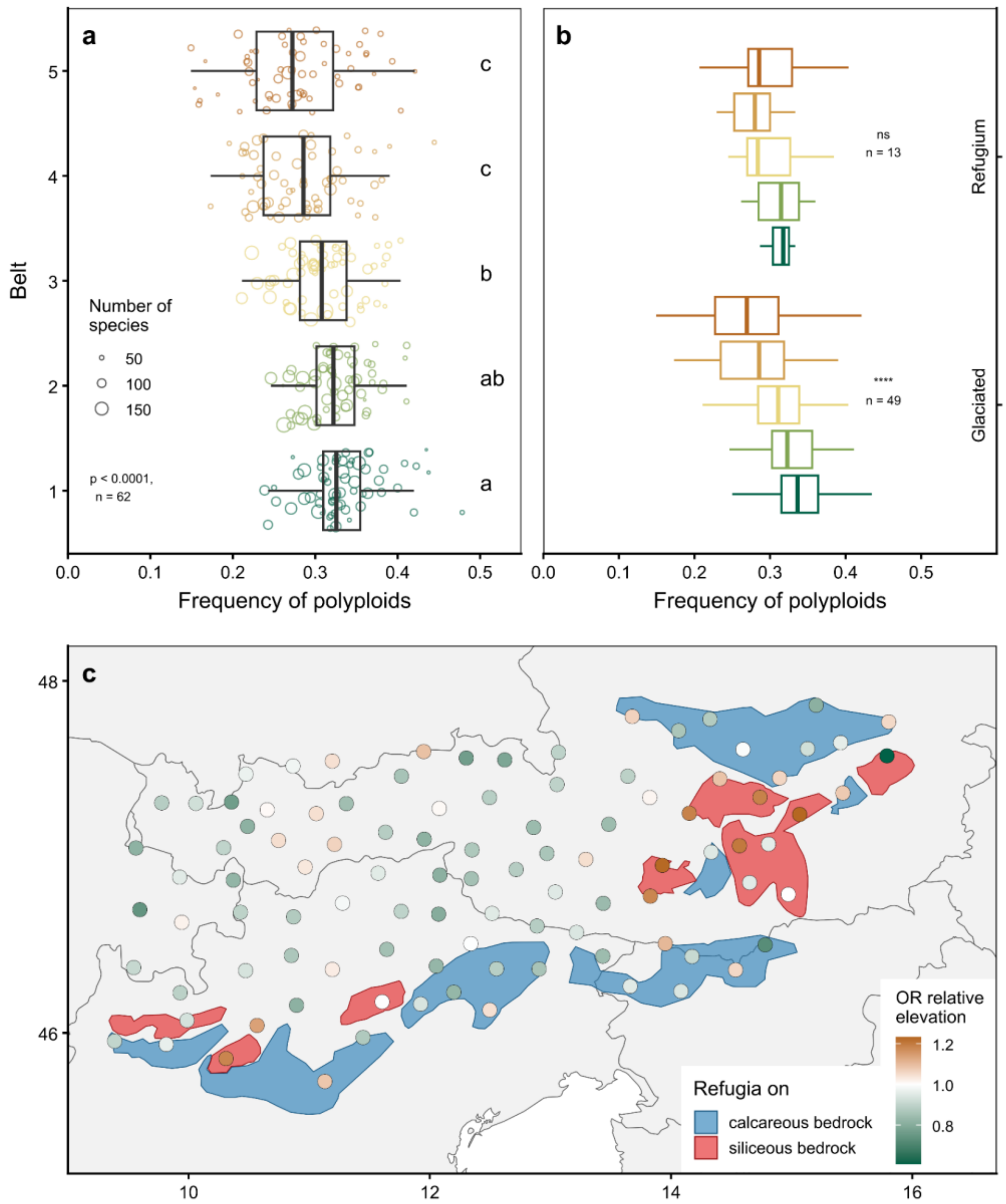

Supplementary Figure 1. Distribution of frequency of polyploid plants across elevation belts in the Eastern Alps using a more permissive definition of polyploidy. Distribution of frequency of polyploid plants across elevation belts in the Eastern Alps. a) Frequency of polyploids across elevation belts (two-sided Friedman test $\chi^2(4) = 51.7$, $p < 0.001$, $n = 62$). Individual points depict 62 sampled mountains, point size reflects the number of species collected per belt. Different letters represent significant differences among belts (based on pairwise Wilcoxon signed-rank tests, two-sided, Holm-adjusted, $p < 0.05$). b) Frequency of polyploids in regions that were glaciated during the Last Glacial Maximum (Friedman test $\chi^2(4) = 55.7$, $p < 0.001$, $n = 49$) and in refugial regions (Friedman test $\chi^2(4) = 2.15$, $p = 0.71$, $n = 13$). Asterisks denote significance levels from Friedman tests (**** $p < 0.0001$; ns, not significant). c) Spatial distribution of odds ratios (OR) for binomial GLMs, quantifying the relationship between polyploid frequency and elevation. Odds ratio > 1 indicates an increasing likelihood of polyploidy with elevation, while OR < 1 indicates a decreasing likelihood. Country boundaries are from Natural Earth[2]. Box plots in panels a and b show the median (center line) and interquartile range (IQR); whiskers extend to the most extreme values within 1.5 × IQR from the box. Source data are provided as a Source Data file. Exact p-values are available in the corresponding Source Data file.

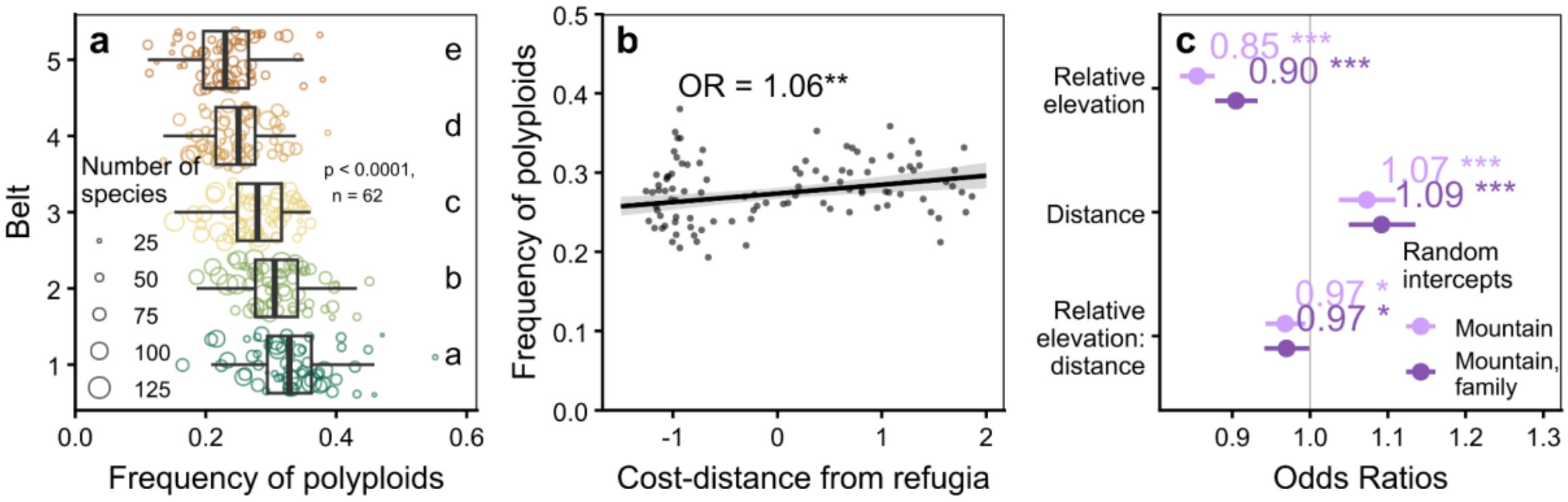

Supplementary Figure 2. Elevational patterns and refugial-distance effects on polyploid frequency in 800 autochorous (slow dispersers) plant species in the Eastern Alps. a) Frequency of polyploids across elevation belts (two-sided Friedman test $\chi^2(4) = 105.0$, $p < 0.001$, $n = 62$). Boxes show the median (center line) and interquartile range (IQR); whiskers extend to the most extreme values within 1.5 × IQR from the box; individual points depict 62 mountains with sufficient sampling density, point size reflects the number of species collected per belt. Different letters indicate significant differences among belts (based on pairwise Wilcoxon signed-rank tests, two-sided, Holm-adjusted, $p < 0.05$). b) Scatterplot of polyploid frequency for each sampling transect (n = 101) versus the average cost-distance from the closest glacial refugium with suitable bedrock. The black line represents a binomial generalised linear mixed model with 95% confidence interval (grey shading; OR = 1.06, 95% CI = 1.02–1.09, two-sided Wald test, $p = 0.002$). Cost-distance was square-root transformed and z-standardised prior to analysis. c) Points show odds ratios with 95% confidence intervals obtained from generalised linear mixed models (GLMMs) with a binomial error distribution. Models included a random intercept for mountain to account for non-independence of samples within the same mountain and, where indicated, an additional plant family-level random intercept to account for possible taxonomic bias. Explanatory variables were z-transformed prior to analysis. Statistical significance of fixed effects was assessed using two-sided Wald tests and is indicated as * $p < 0.05$, ** $p < 0.01$, *** $p < 0.001$. Source data are provided as a Source Data file. Exact p-values are available in the corresponding Source Data file.

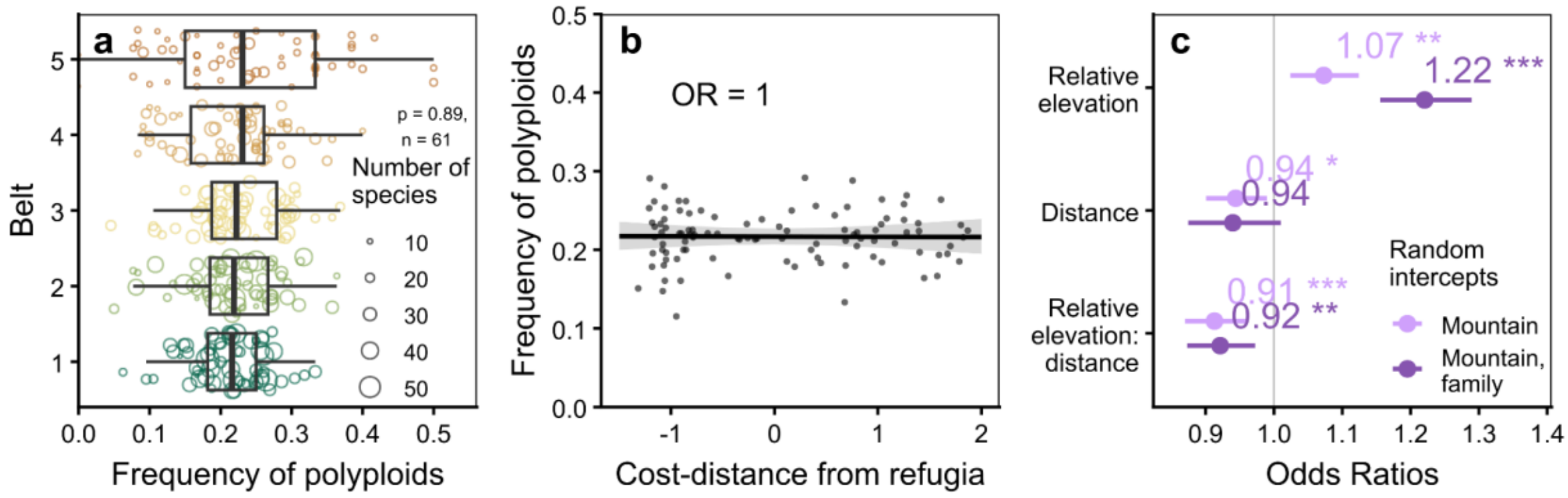

Supplementary Figure 3. Elevational patterns and refugial-distance effects on polyploid frequency in 303 anemo-, hydro-, and zoochorous plant species in the Eastern Alps. a) Frequency of polyploids across elevation belts (two-sided Friedman test $\chi^2(4) = 1.13$, $p = 0.89$, $n = 61$). Boxes show the median (center line) and interquartile range (IQR); whiskers extend to the most extreme values within 1.5 × IQR from the box; individual points depict 61 mountains with sufficient sampling density, point size reflects the number of species collected per belt. b) Scatterplot of polyploid frequency for each sampling transect (n = 101) versus the average cost-distance from the closest glacial refugium with suitable bedrock. The black line represents a binomial generalised linear mixed model with 95% confidence interval (grey shading; OR = 1.00, 95% CI = 0.94–1.06, two-sided Wald test, p = 0.95). Cost-distance was square-root transformed and z-standardised prior to analysis. c) Points show odds ratios with 95% confidence intervals obtained from generalised linear mixed models (GLMMs) with a binomial error distribution. Models included a random intercept for mountain to account for non-independence of samples within the same mountain and, where indicated, an additional plant family-level random intercept to account for possible taxonomic bias. Explanatory variables were z-transformed prior to analysis. Statistical significance of fixed effects was assessed using two-sided Wald tests and is indicated as * $p < 0.05$, ** $p < 0.01$, *** $p < 0.001$. Source data are provided as a Source Data file. Exact p-values are available in the corresponding Source Data file.

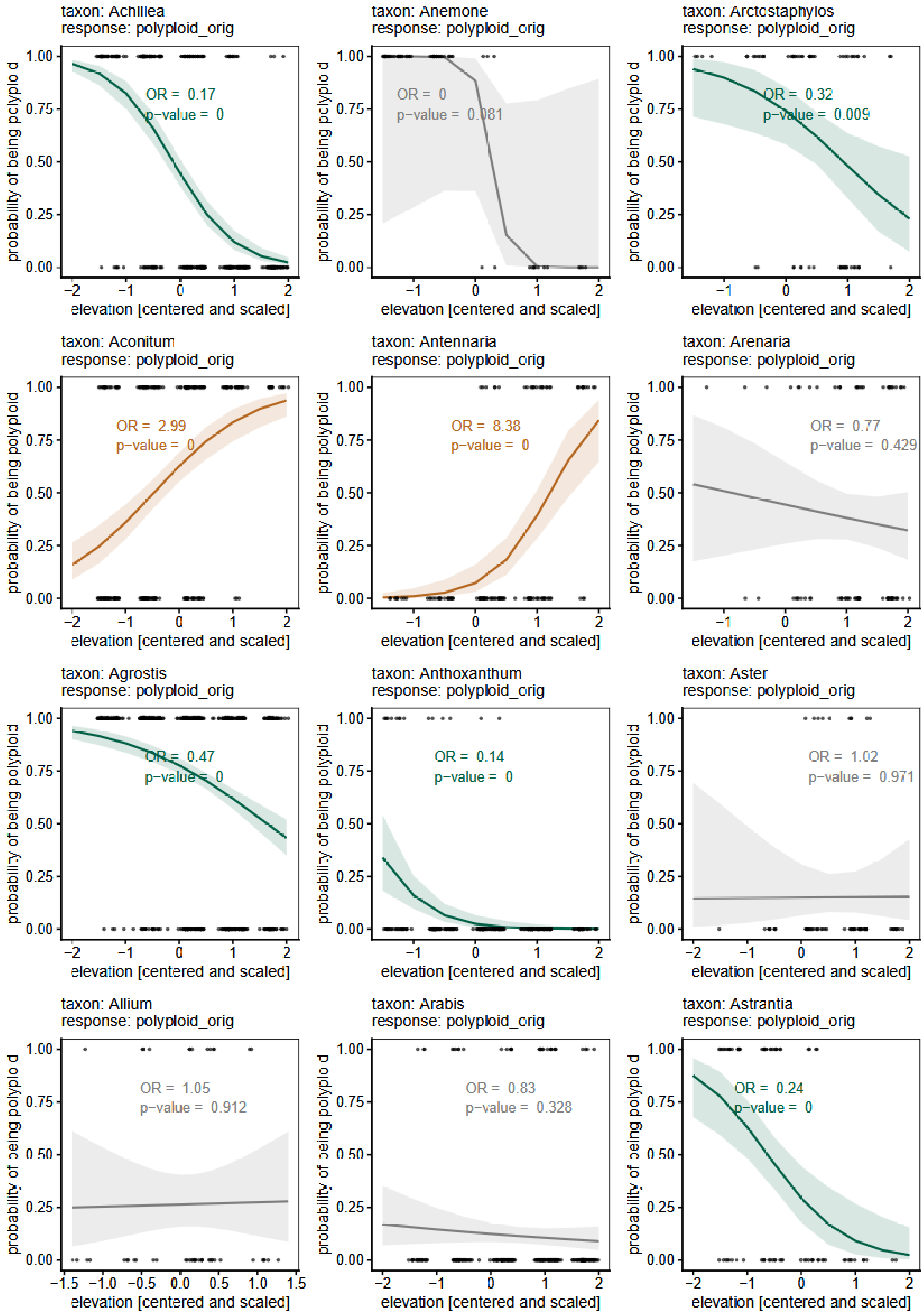

Supplementary FIgure 4, continues on the next page

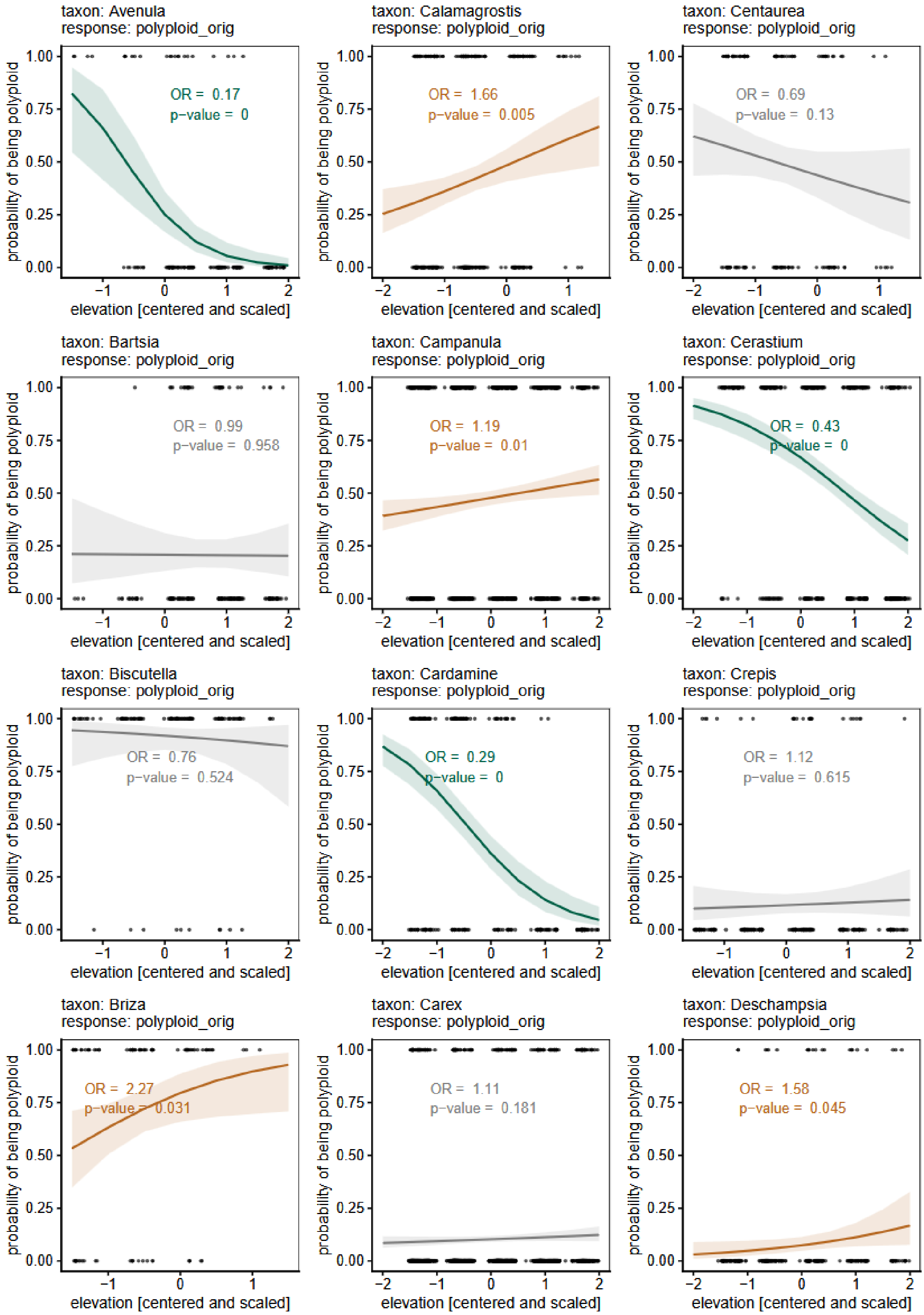

Supplementary FIgure 4, continues on the next page

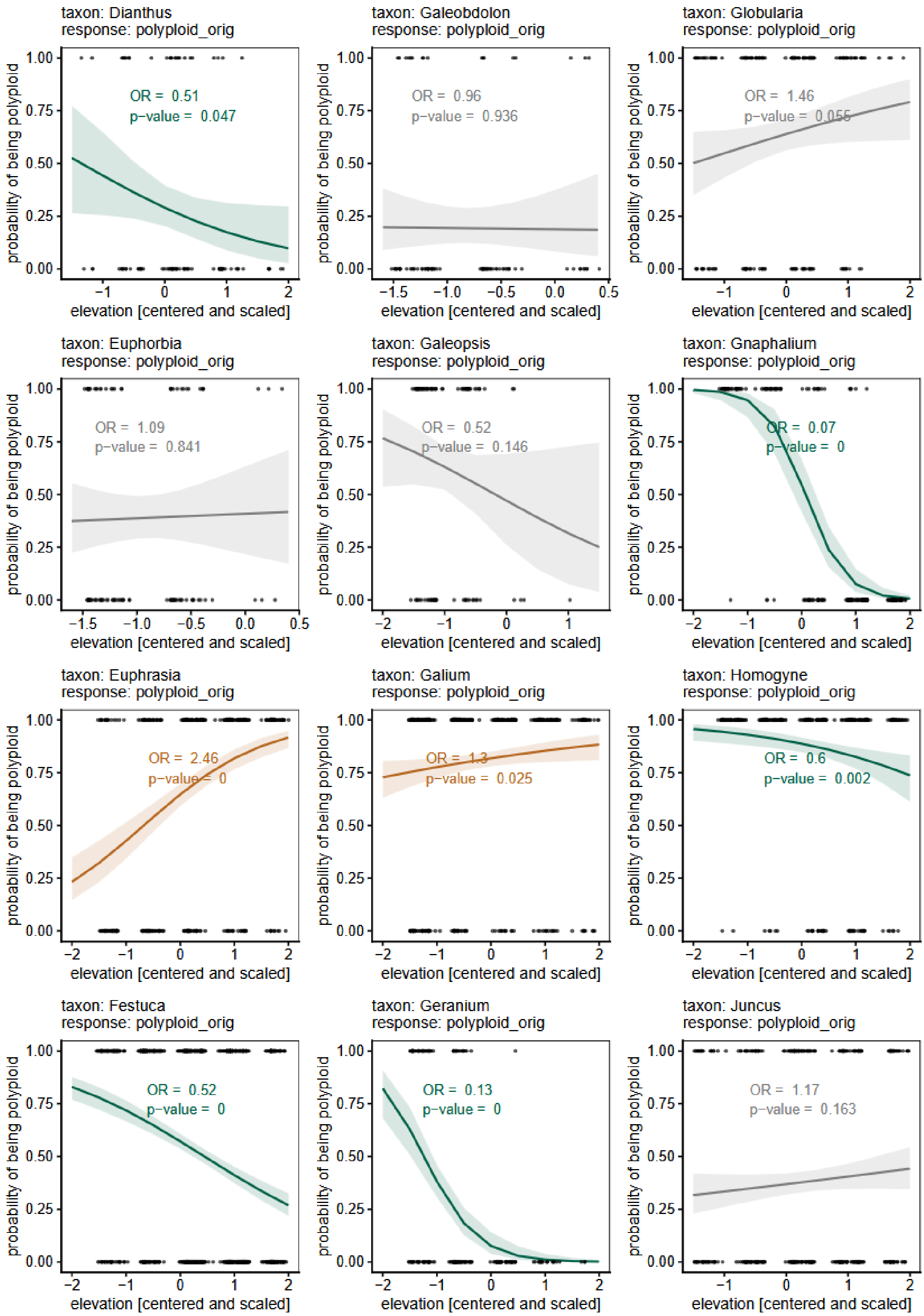

Supplementary FIgure 4, continues on the next page

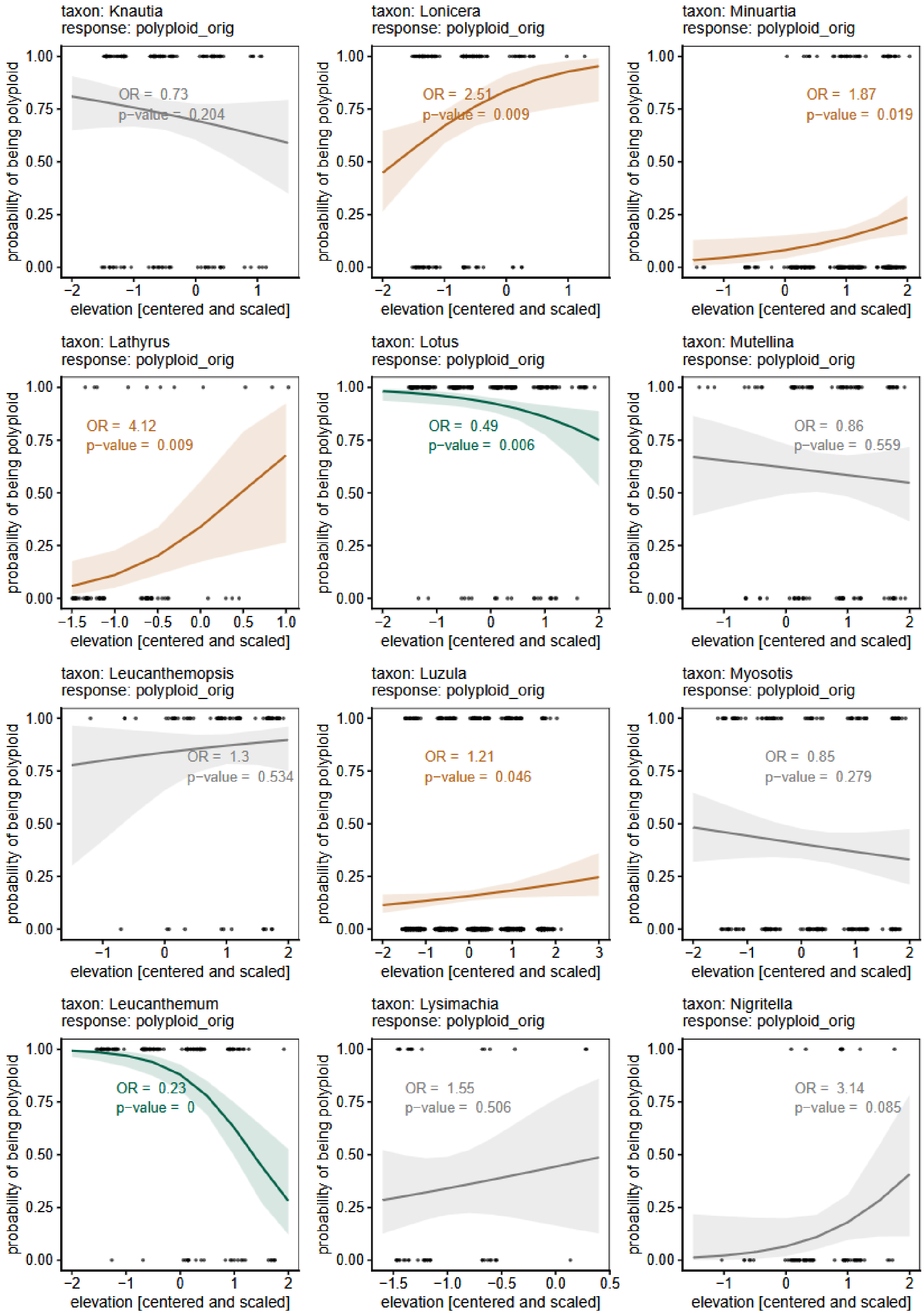

Supplementary FIgure 4, continues on the next page

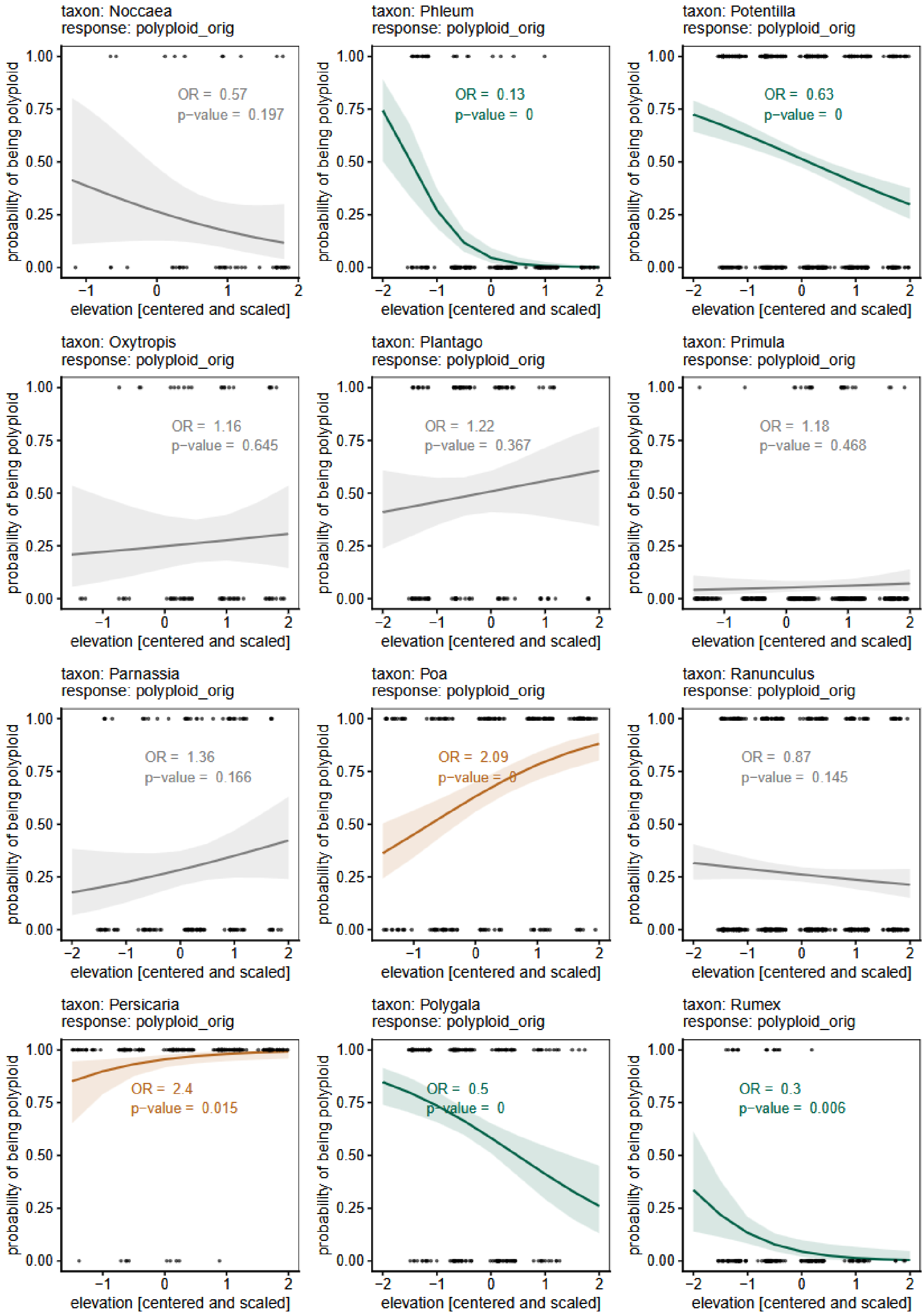

Supplementary FIgure 4, continues on the next page

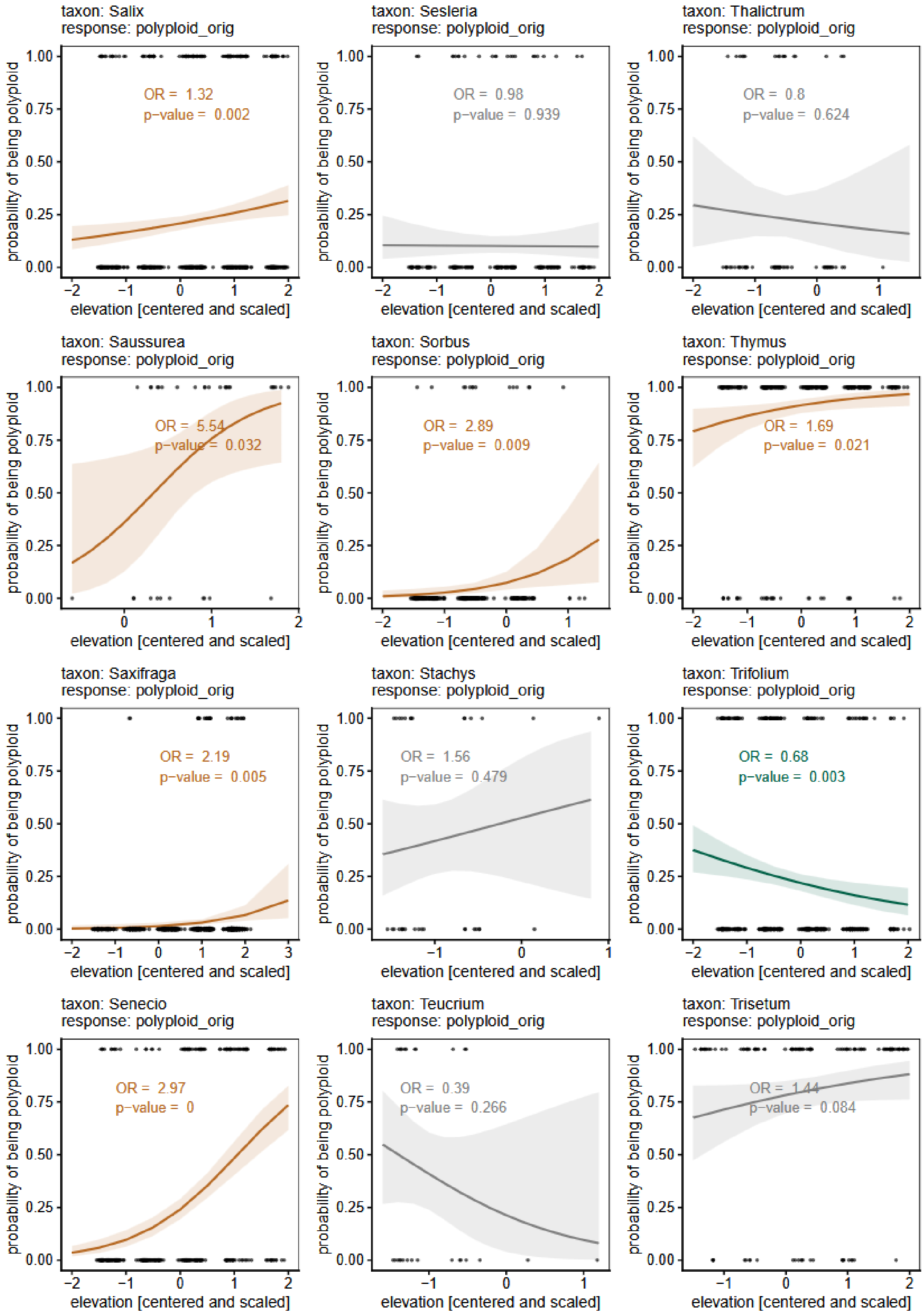

Supplementary FIgure 4, continues on the next page

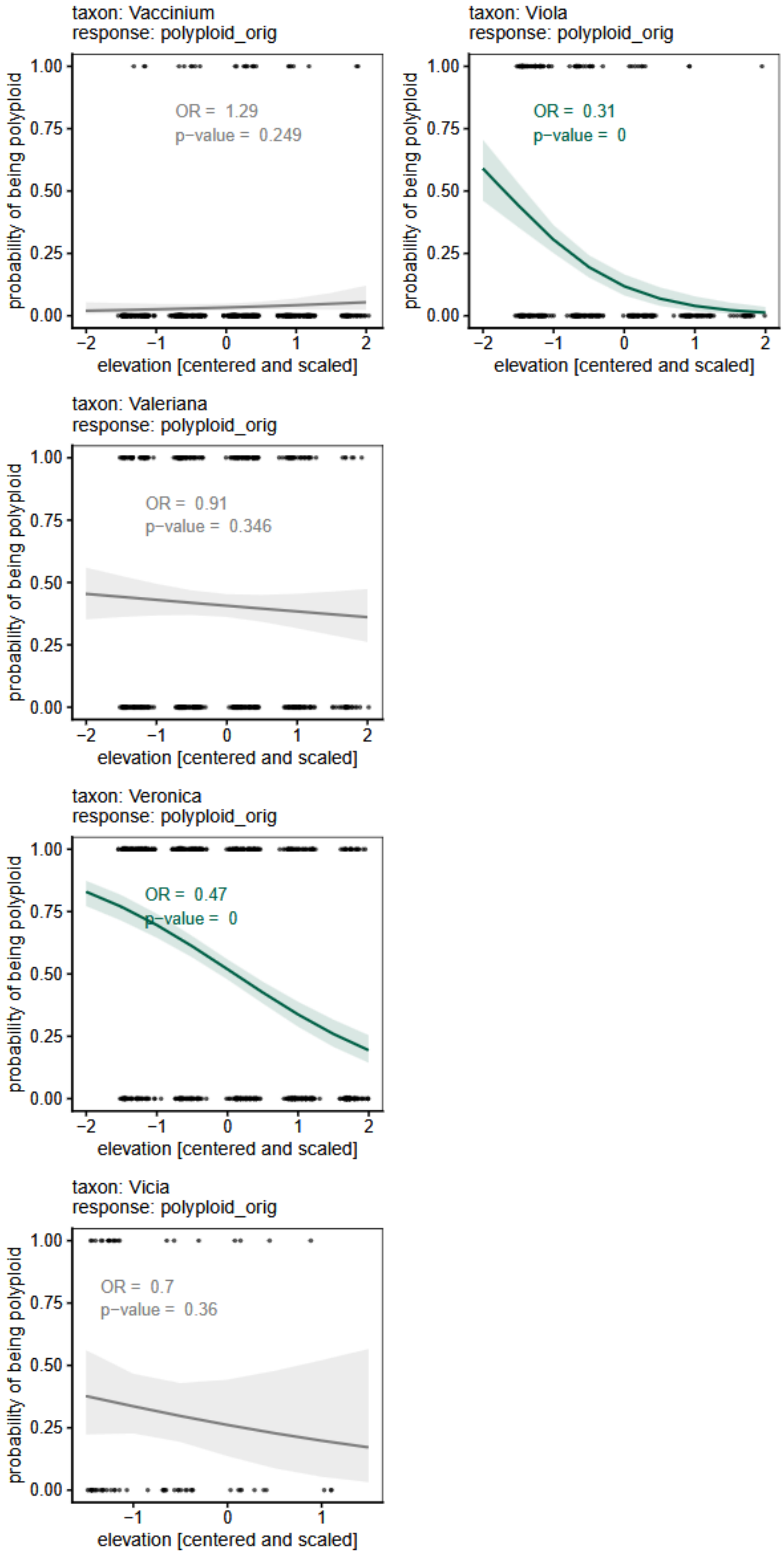

Supplementary Figure 4. Genus-specific model predictions from binomial GLMs (elevation). Predicted probabilities of being polyploidy as a function of relative elevation, based on genus-specific binomial generalised linear models (GLMs) fitted with a logit link. Each panel shows model predictions and observed data for a single mixed-ploidy genus. Lines represent fitted relationships between elevation and the probability of being polyploid, with line colors indicating the direction and significance of the elevation effect (brown = significant positive, green = significant negative, grey = non-significant; $p < 0.05$). Points show observed data (polyploid = 1, diploid = 0). Source data are provided as a Source Data file. Exact p-values from two-sided Wald tests are available in the corresponding Source Data file.

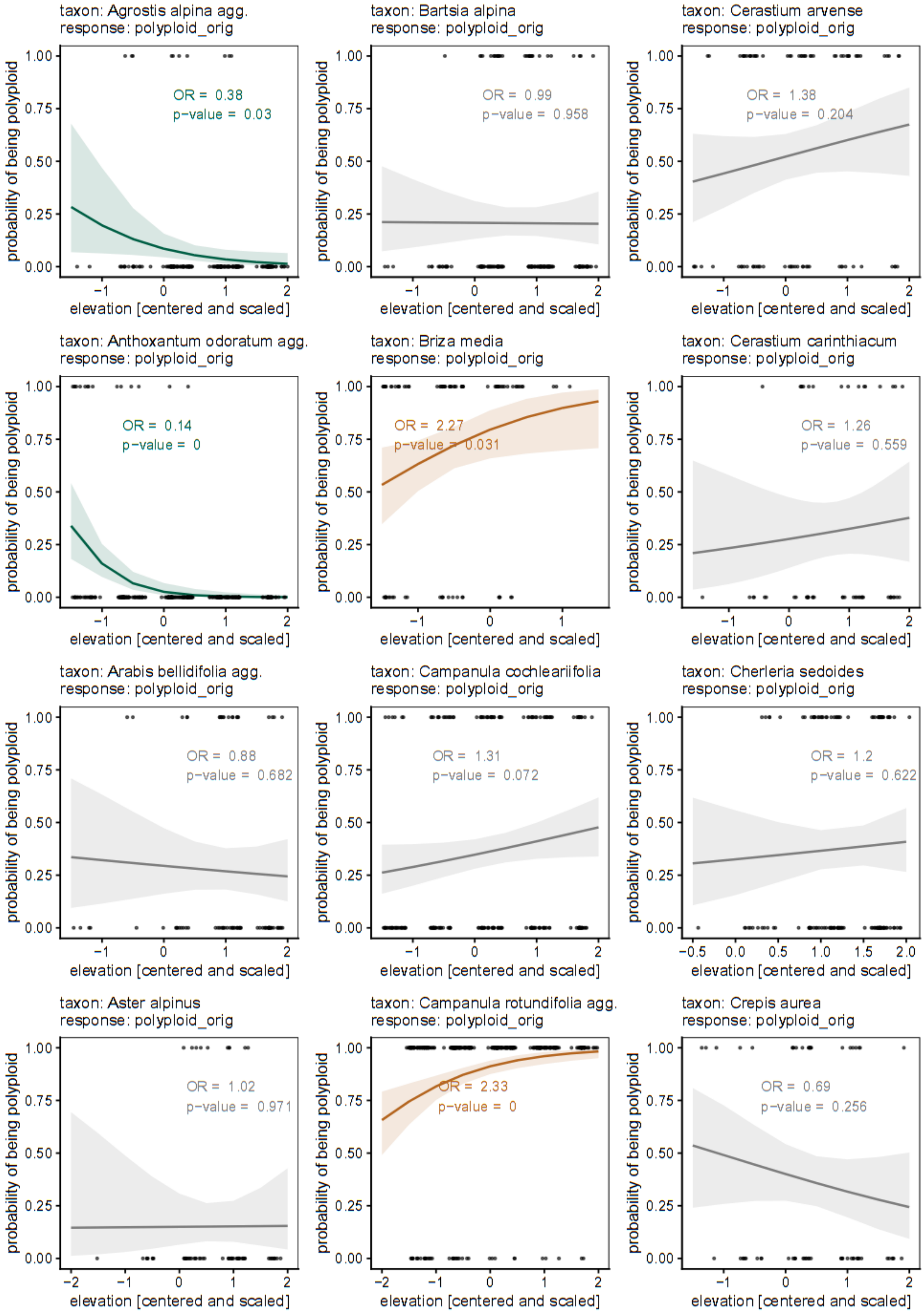

Supplementary FIgure 5, continues on the next page

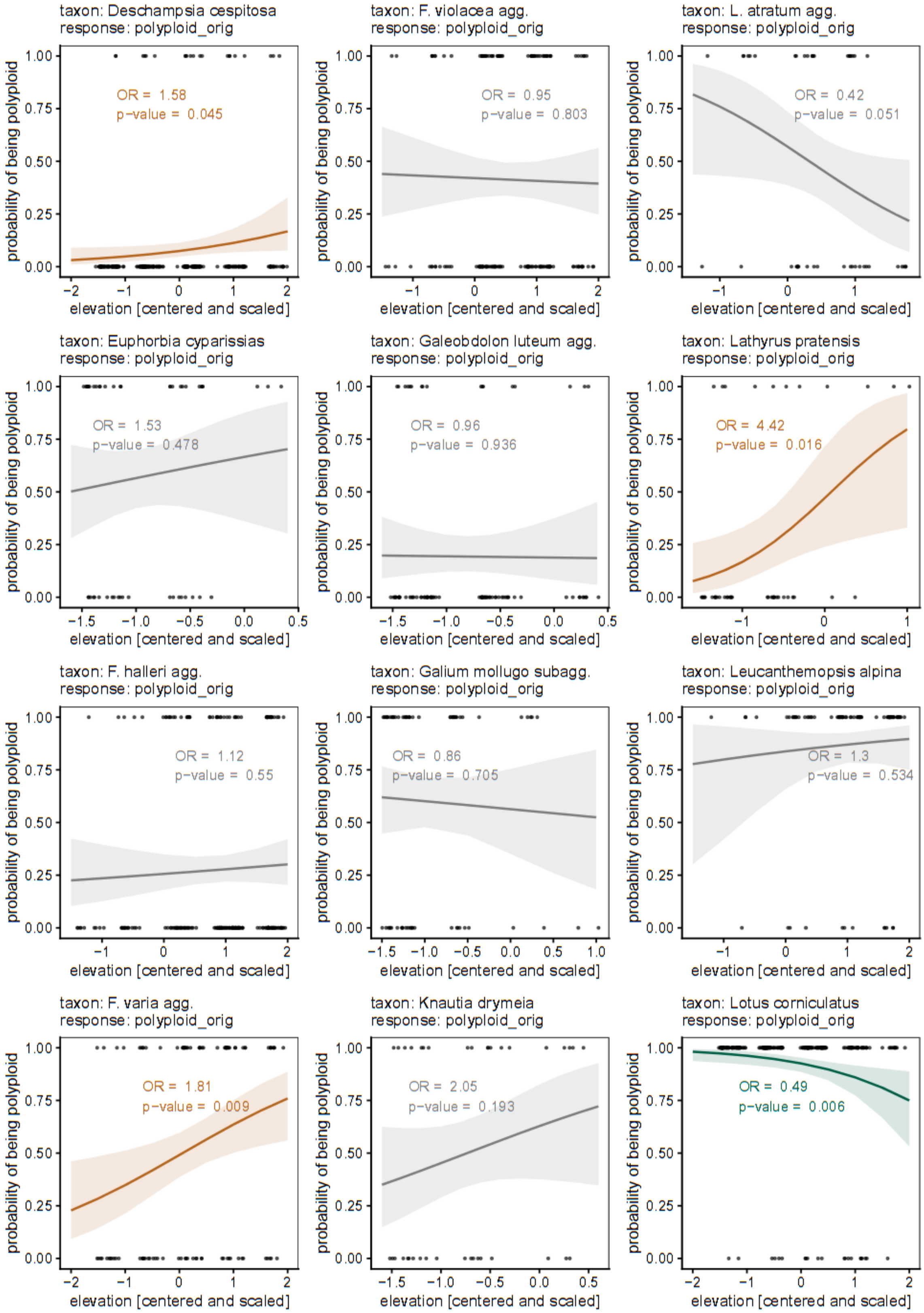

Supplementary FIgure 5, continues on the next page

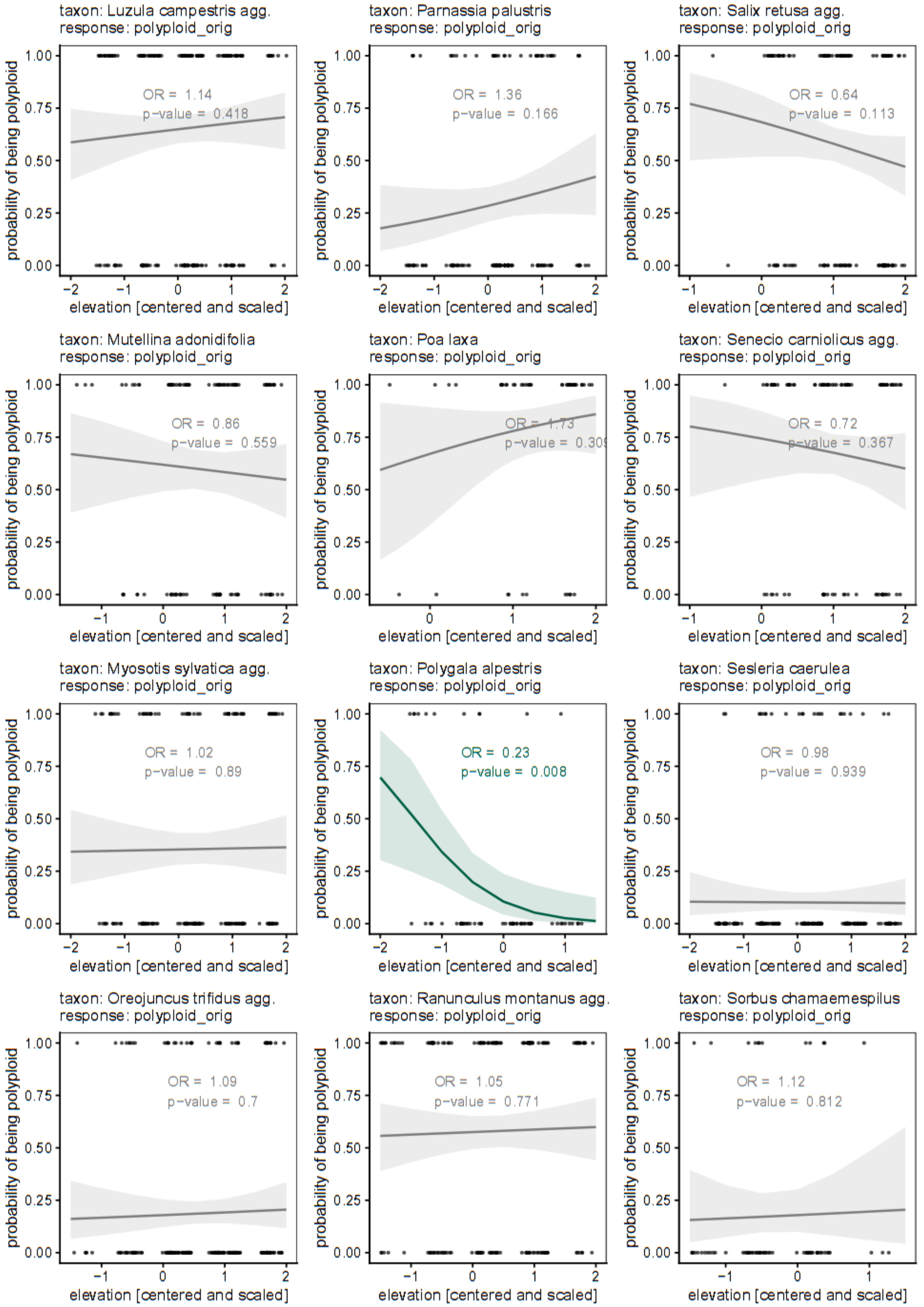

Supplementary FIgure 5, continues on the next page

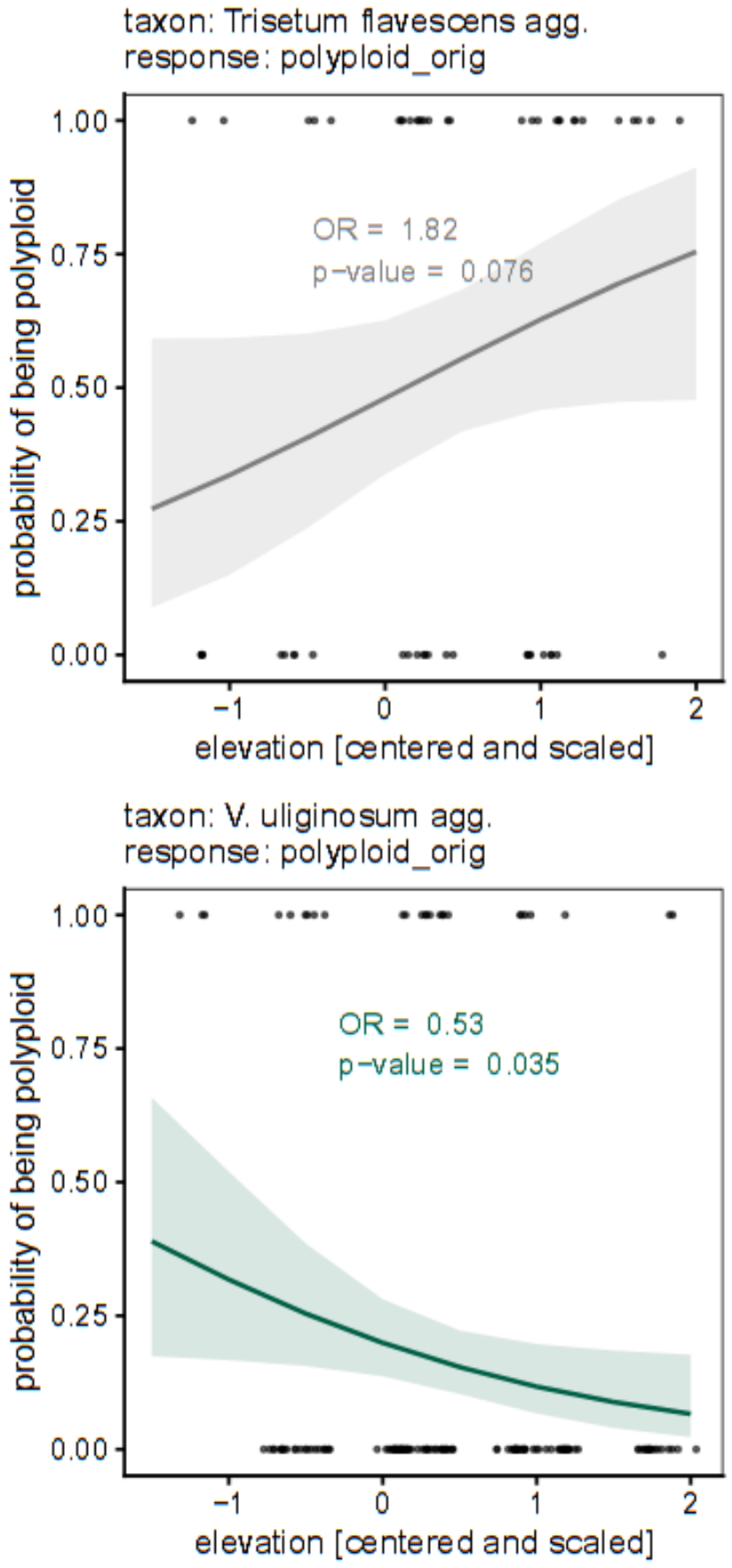

Supplementary Figure 5. Species-specific model predictions from binomial GLMs (elevation). Predicted probabilities of being polyploidy as a function of relative elevation, based on taxon-specific binomial generalised linear models (GLMs) fitted with a logit link. Each panel shows model predictions and observed data for a single mixed-ploidy species or aggregate. Lines represent fitted relationships between elevation and the probability of being polyploid, with line colors indicating the direction and significance of the elevation effect (brown = significant positive, green = significant negative, grey = non-significant; $p < 0.05$). Points show observed data (polyploid = 1, diploid = 0). Source data are provided as a Source Data file. Exact p-values from two-sided Wald tests are available in the corresponding Source Data file.

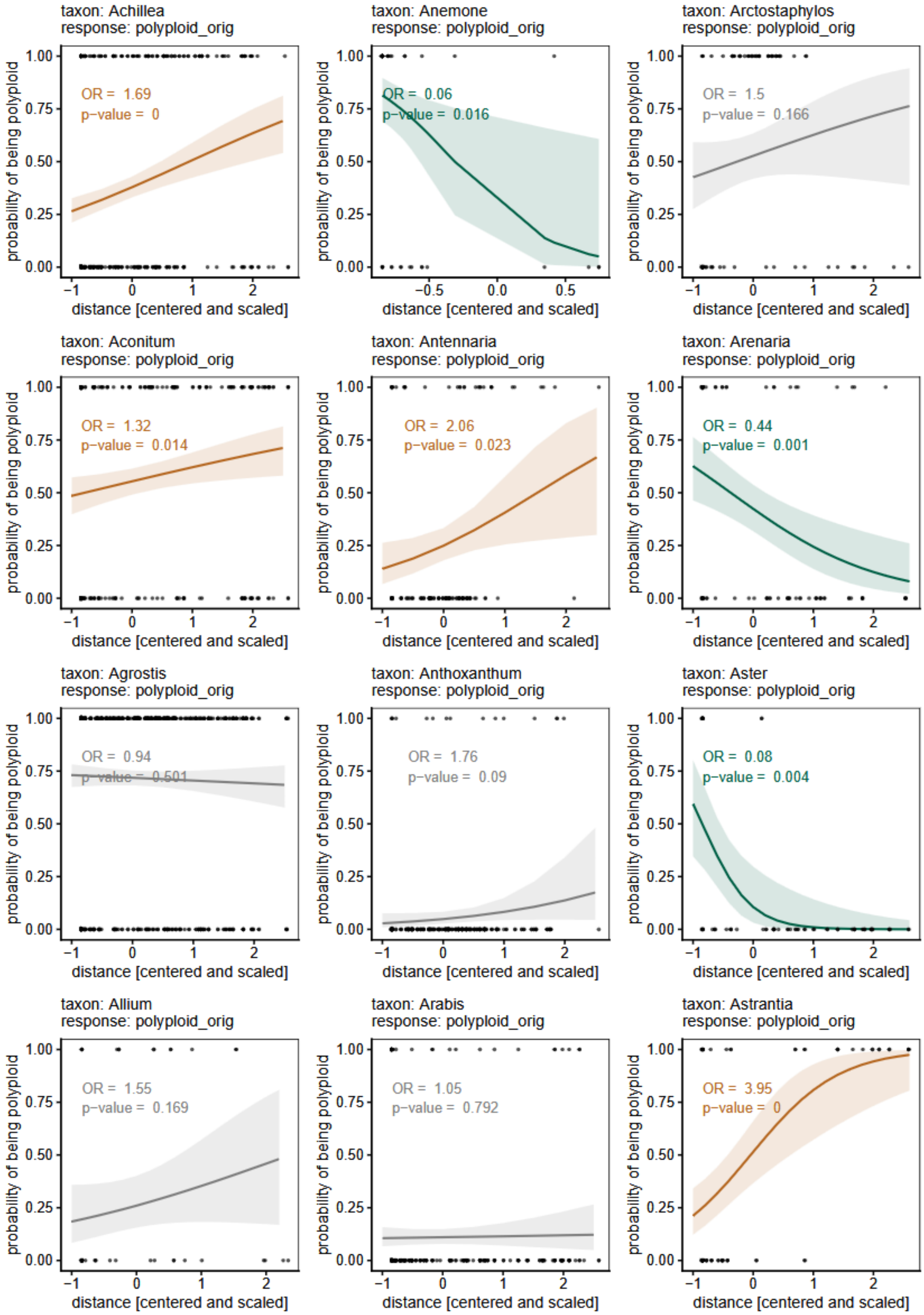

Supplementary FIgure 6, continues on the next page

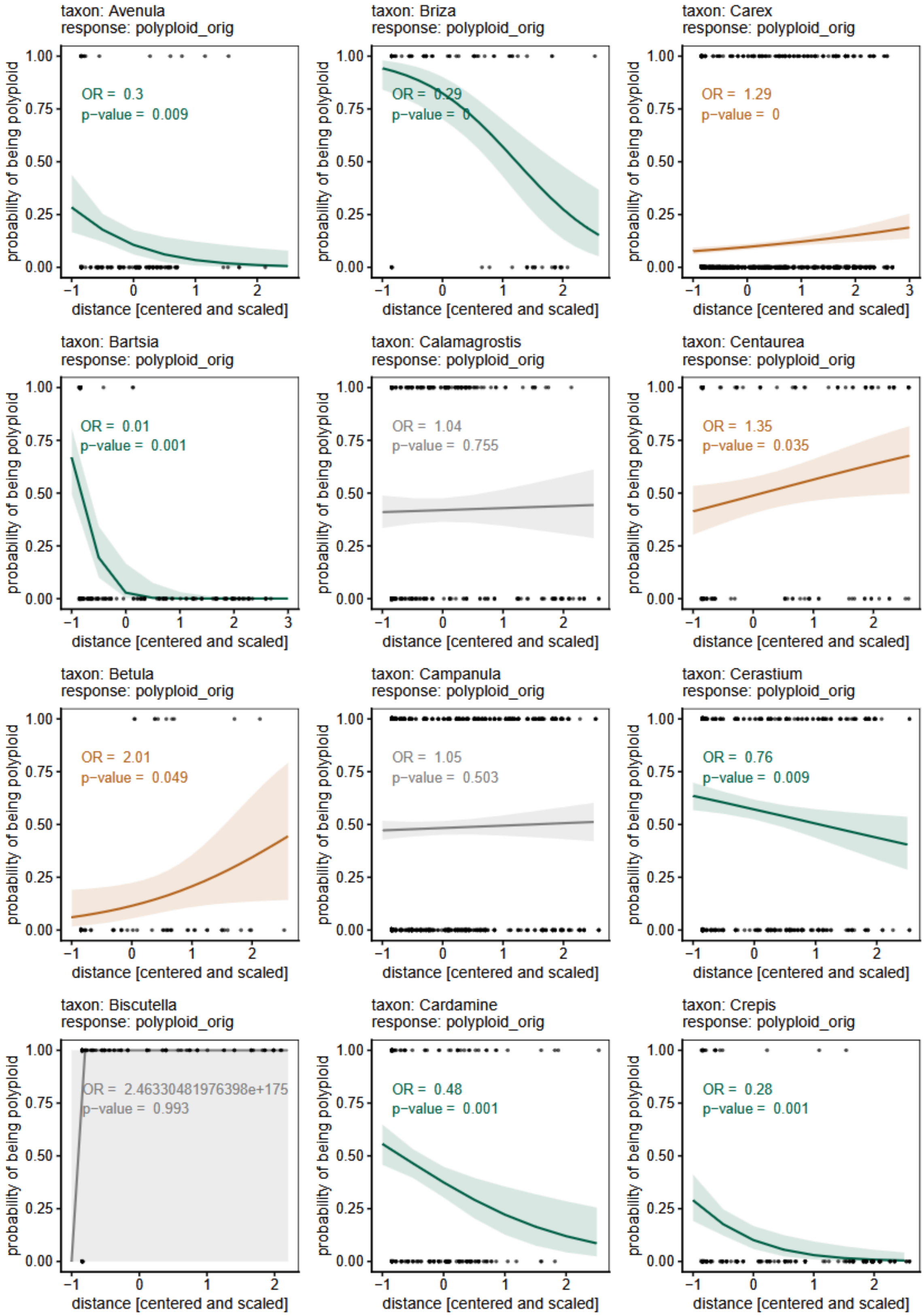

Supplementary FIgure 6, continues on the next page

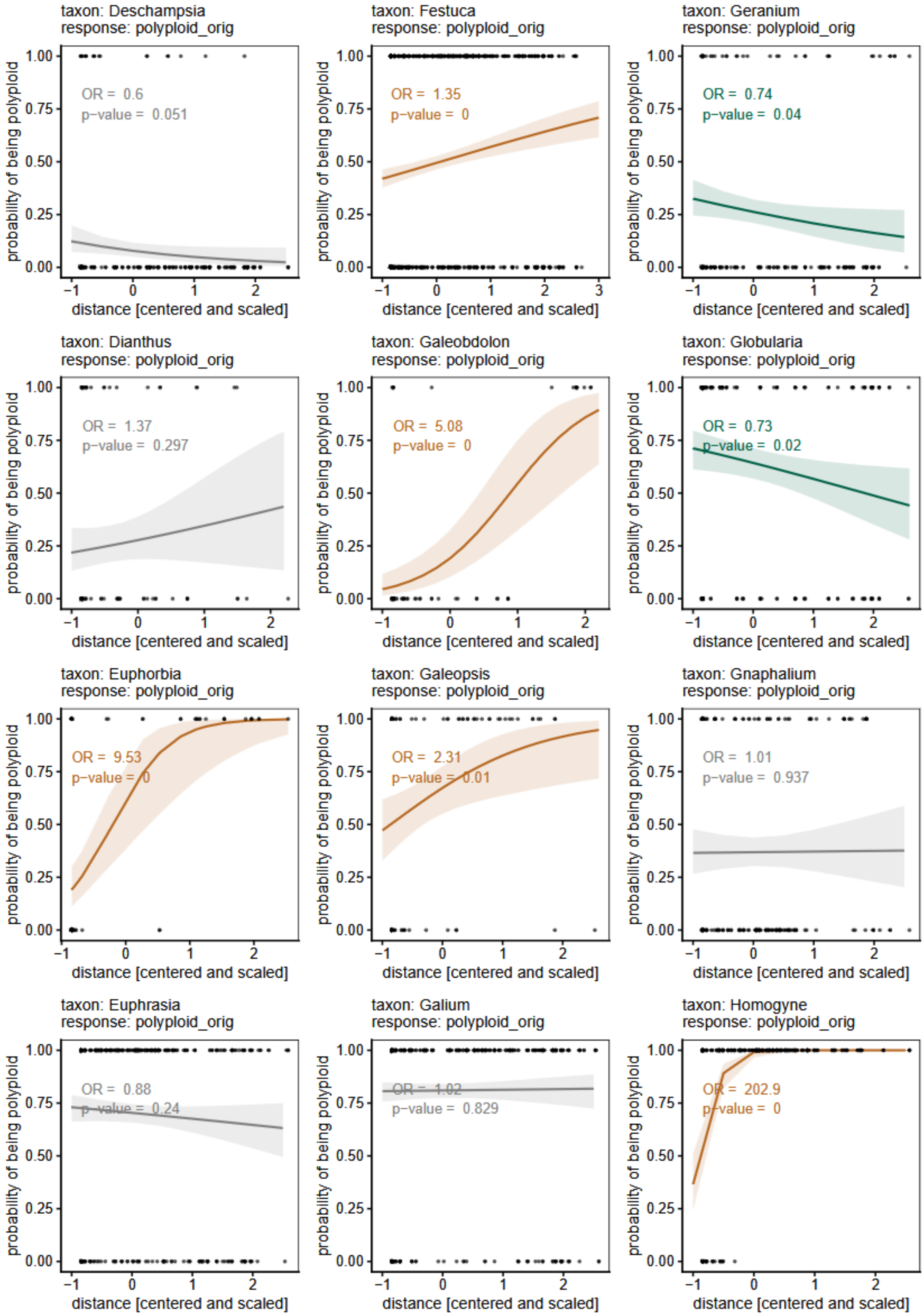

Supplementary FIgure 6, continues on the next page

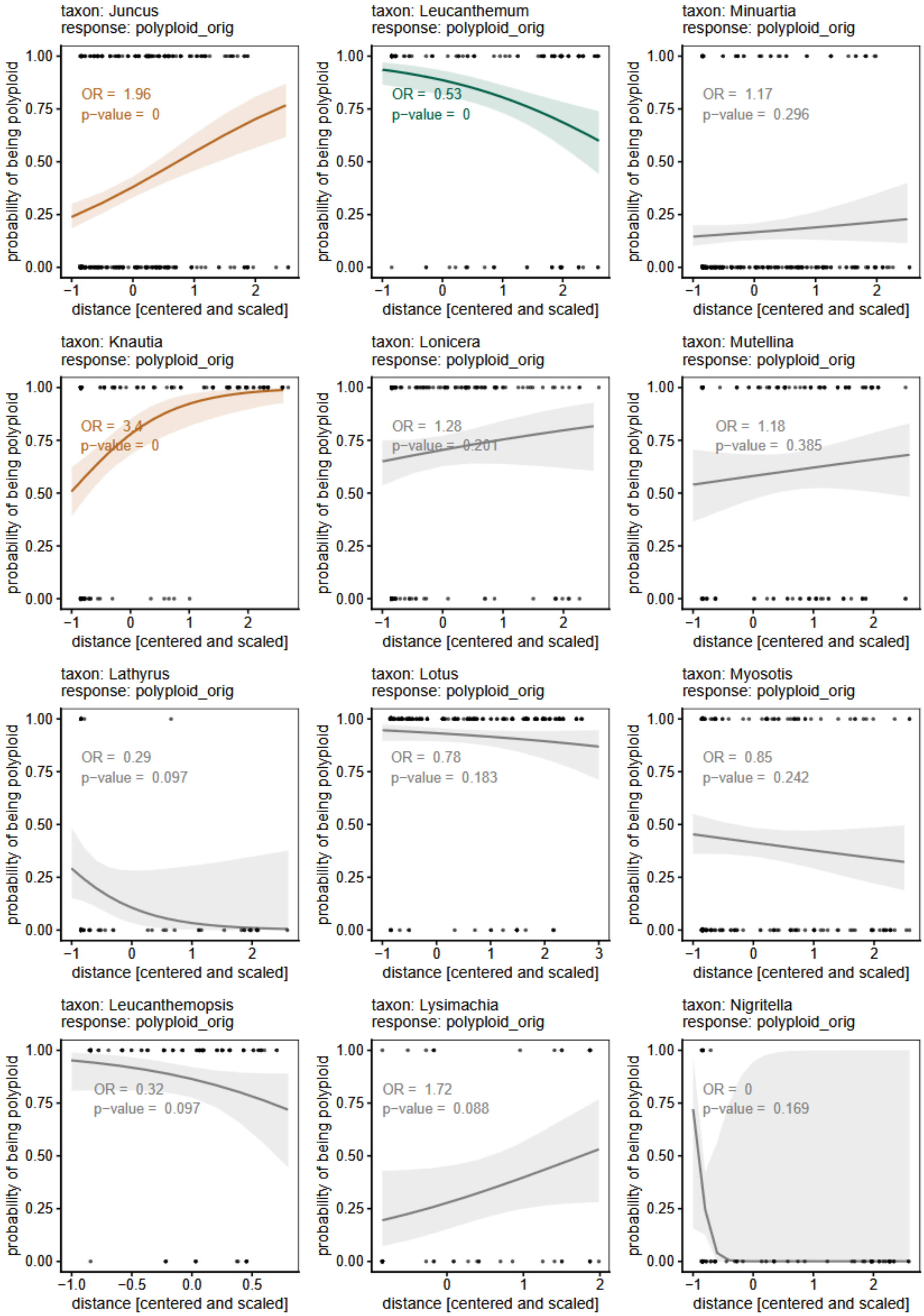
taxon: Juncus
response: polyploid_orig
OR = 1.96
p−value = 0
taxon: Leucanthemum
response: polyploid_orig
OR = 0.53
p−value = 0
taxon: Minuartia
response: polyploid_orig
OR = 1.17
p−value = 0.296
taxon: Knautia
response: polyploid_orig
OR = 3.4
p−value = 0
taxon: Lonicera
response: polyploid_orig
OR = 1.28
p−value = 0.201
taxon: Mutellina
response: polyploid_orig
OR = 1.18
p−value = 0.385
taxon: Lathyrus
response: polyploid_orig
OR = 0.29
p−value = 0.097
taxon: Lotus
response: polyploid_orig
OR = 0.78
p−value = 0.183
taxon: Myosotis
response: polyploid_orig
OR = 0.85
p−value = 0.242
taxon: Leucanthemopsis
response: polyploid_orig
OR = 0.32
p−value = 0.097
taxon: Lysimachia
response: polyploid_orig
OR = 1.72
p−value = 0.088
taxon: Nigritella
response: polyploid_orig
OR = 0
p−value = 0.169
probability of being polyploid
distance [centered and scaled]

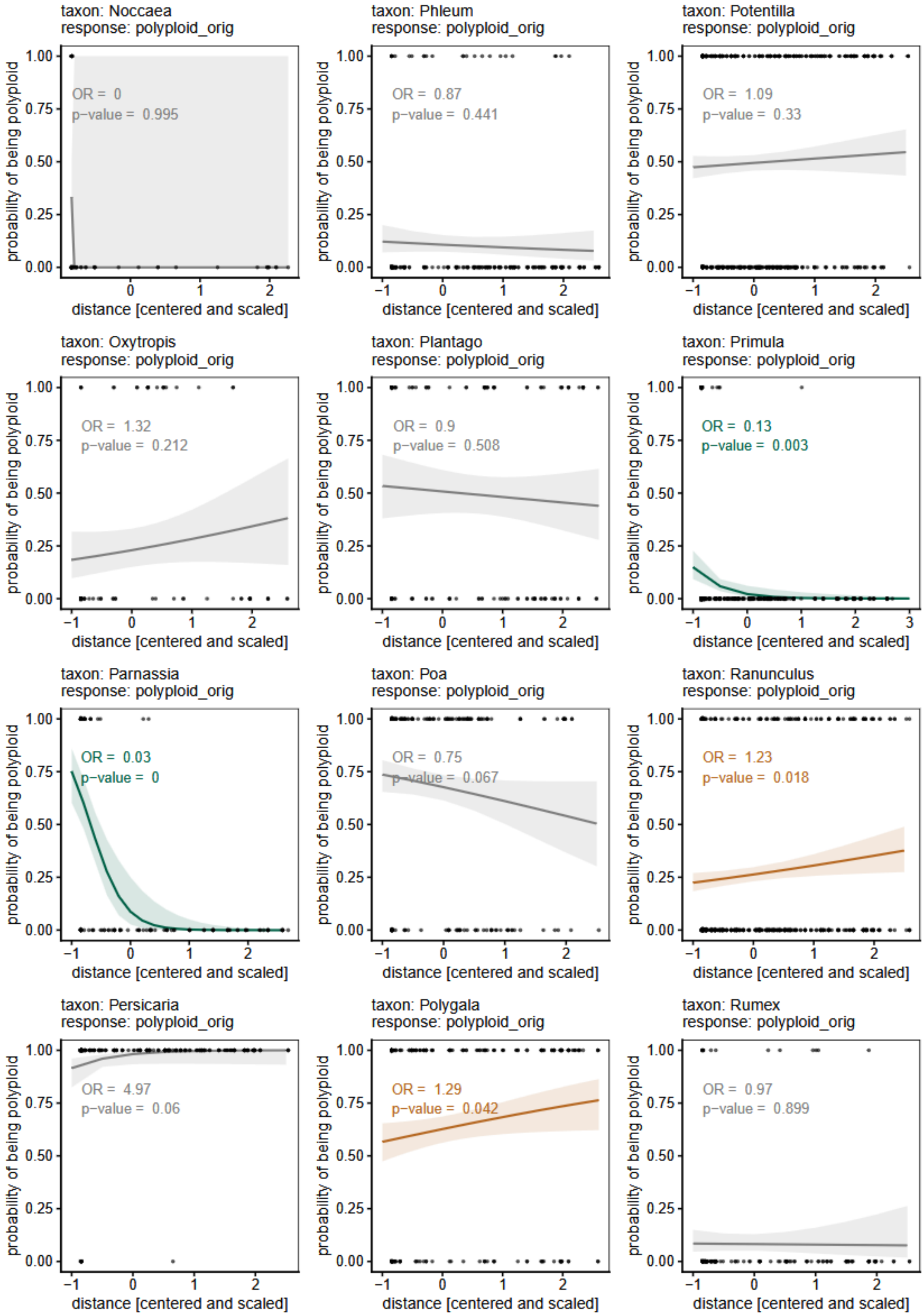

Supplementary FIgure 6, continues on the next page

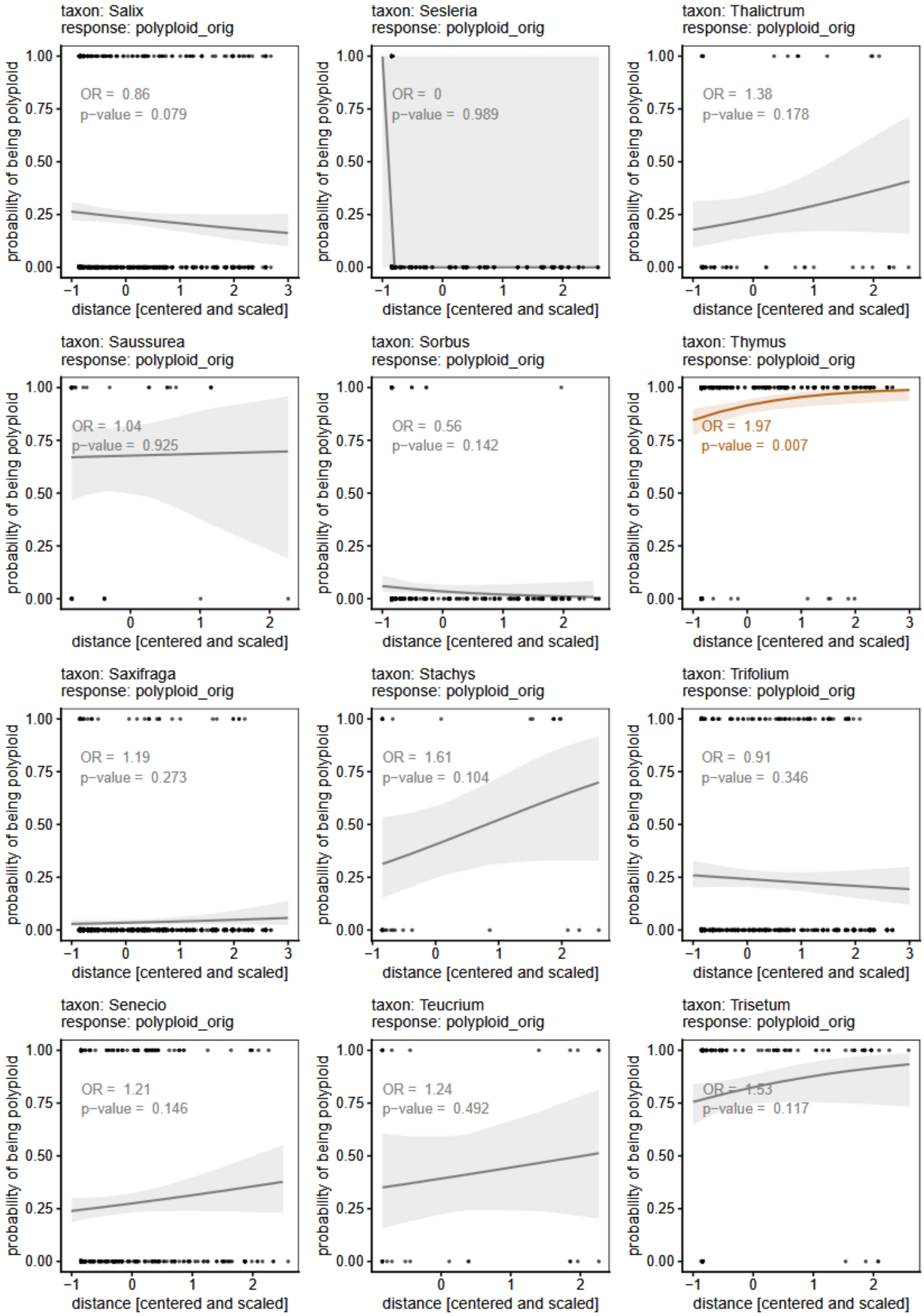

Supplementary FIgure 6, continues on the next page

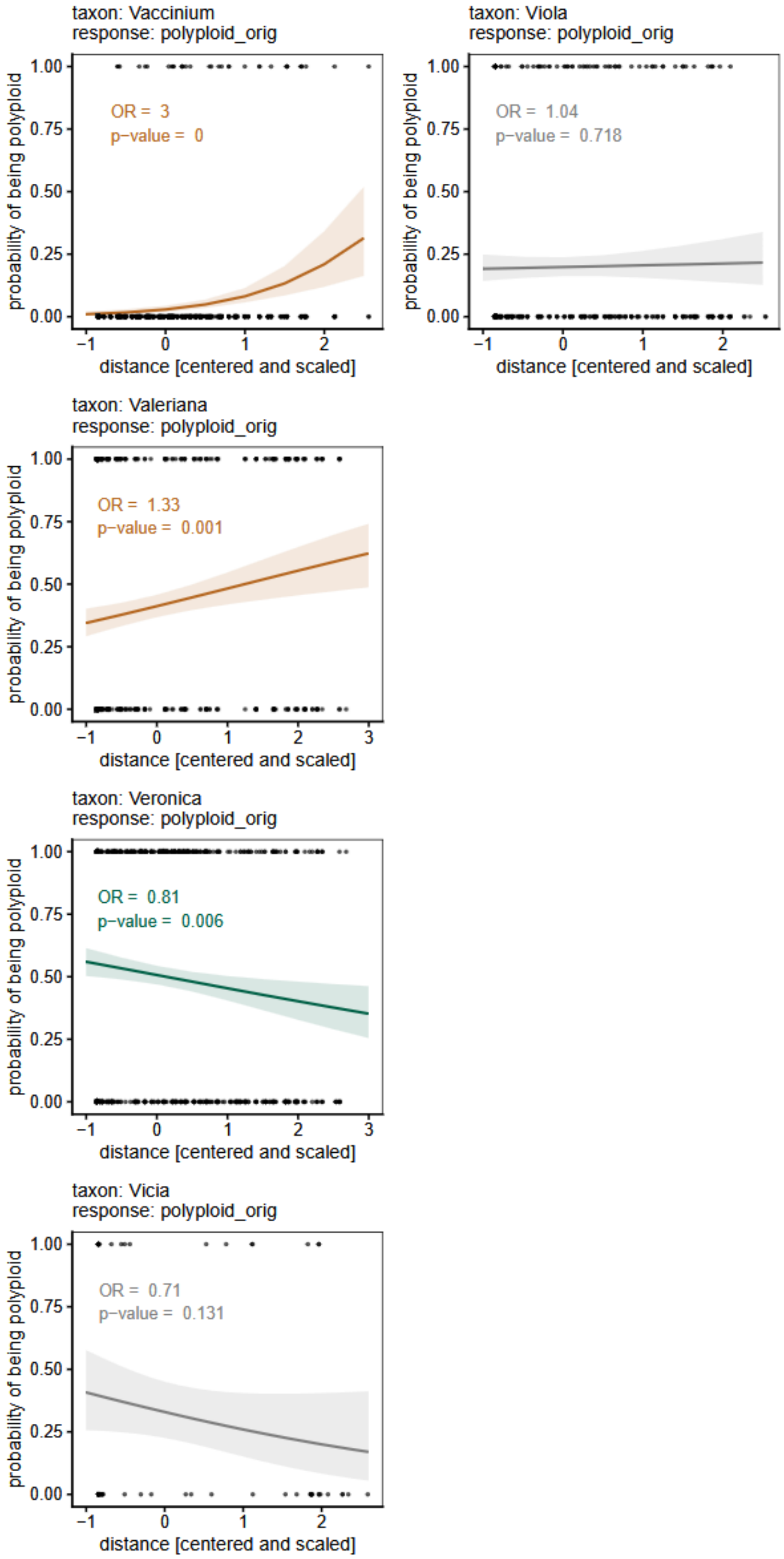

Supplementary Figure 6. Genus-specific model predictions from binomial GLMs (distance). Predicted probabilities of being polyploidy as a function of cost-distance, based on genus-specific binomial generalised linear models (GLMs) fitted with a logit link. Each panel shows model predictions and observed data for a single mixed-ploidy genus. Lines represent fitted relationships between cost-distance and the probability of being polyploid, with line colors indicating the direction and significance of the cost-distance effect (brown = significant positive, green = significant negative, grey = non-significant; $p < 0.05$). Points show observed data (polyploid = 1, diploid = 0). Source data are provided as a Source Data file. Exact p-values from two-sided Wald tests are available in the corresponding Source Data file.

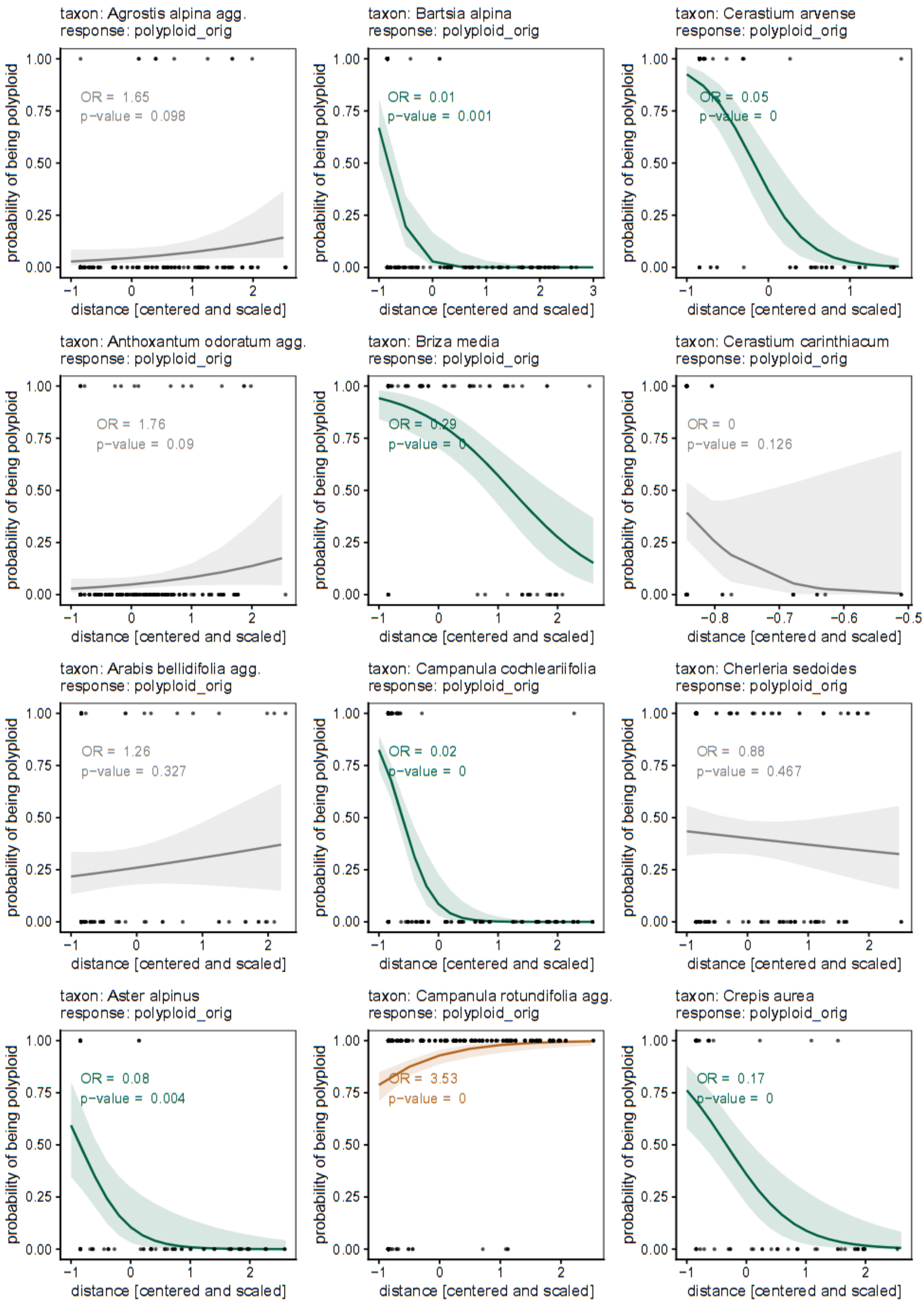

Supplementary FIgure 7, continues on the next page

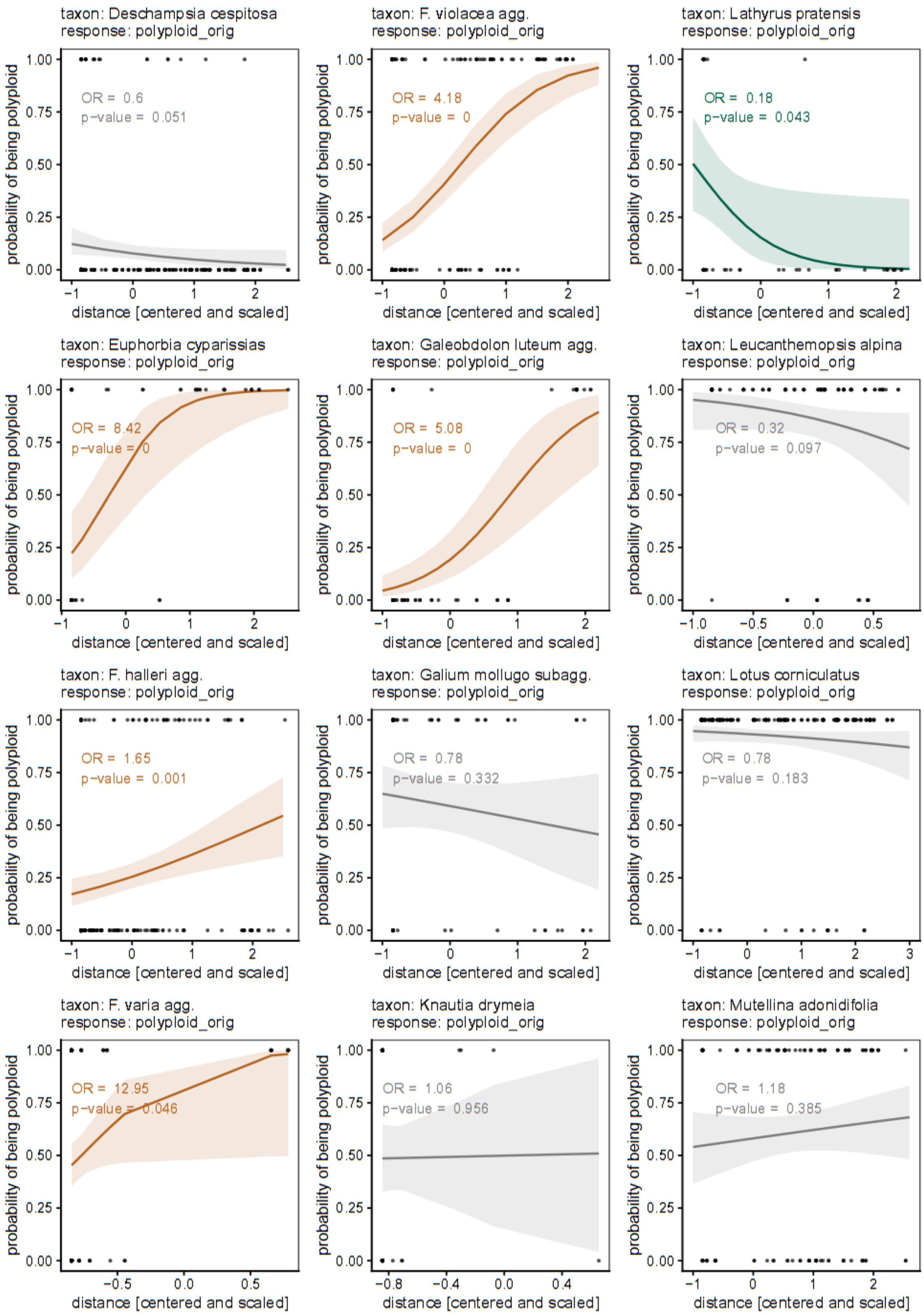

Supplementary FIgure 7, continues on the next page

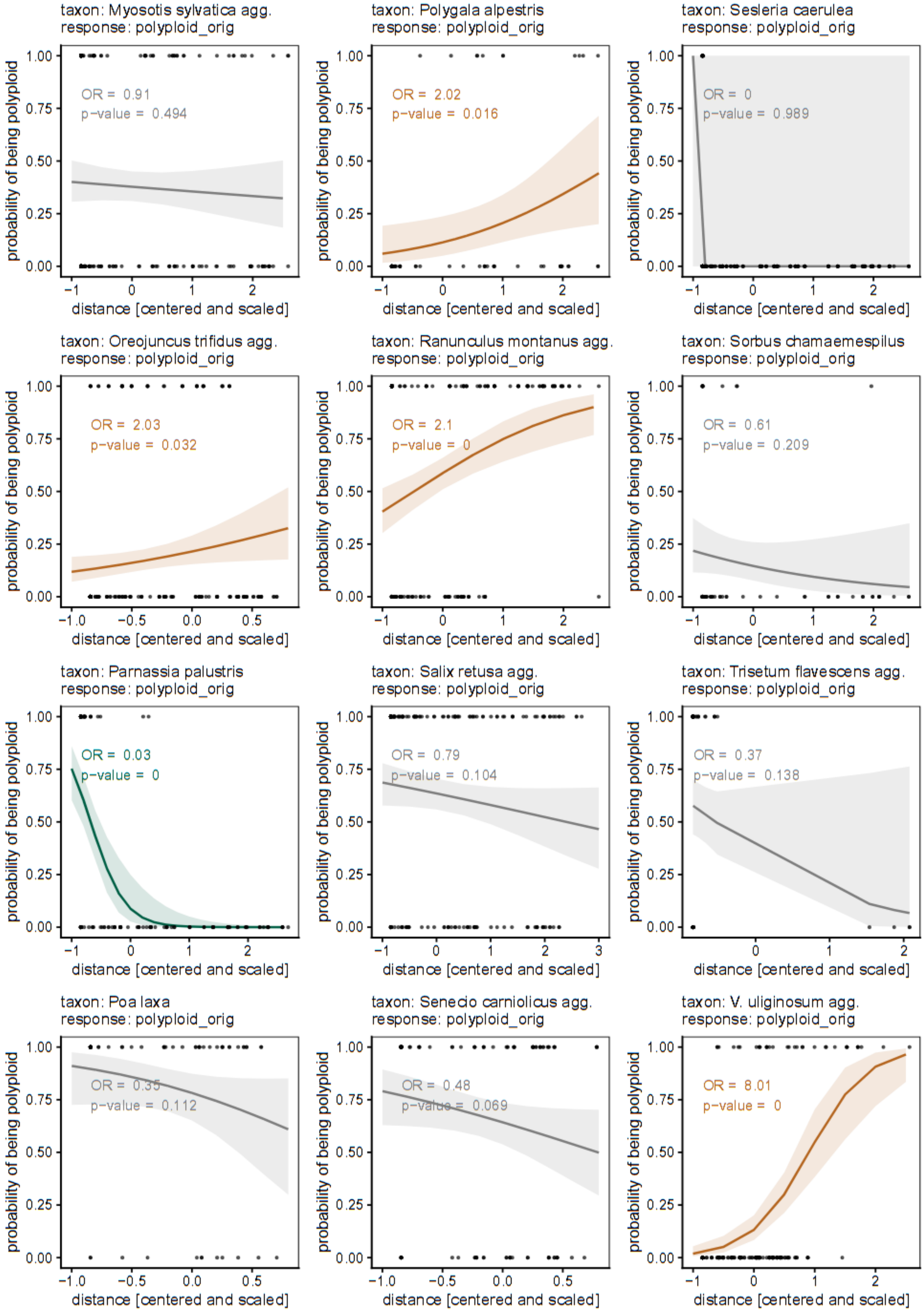

Supplementary Figure 7. Species-specific model predictions from binomial GLMs (distance). Predicted probabilities of being polyploidy as a function of cost-distance, based on taxon-specific binomial generalised linear models (GLMs) fitted with a logit link. Each panel shows model predictions and observed data for a single mixed-ploidy species or aggregate. Lines represent fitted relationships between cost-distance and the probability of being polyploid, with line colors indicating the direction and significance of the cost-distance effect (brown = significant positive, green = significant negative, grey = non-significant; $p < 0.05$). Points show observed data (polyploid = 1, diploid = 0). Source data are provided as a Source Data file. Exact p-values from two-sided Wald tests are available in the corresponding Source Data file.

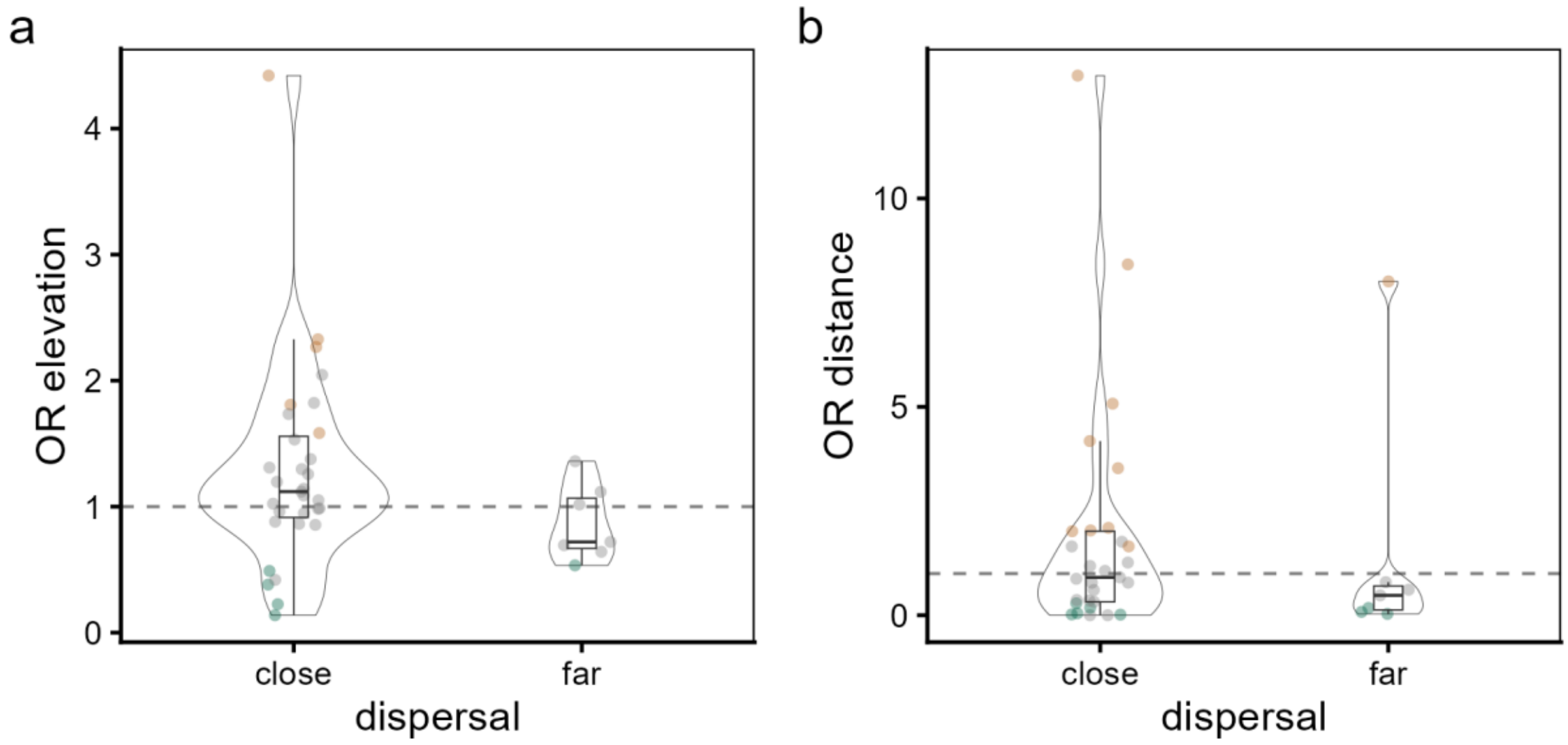

Supplementary Figure 8. Species-level elevation and distance effects on polyploidy. Distribution of taxon-specific odds ratios (ORs) from binomial GLMs testing the association between polyploidy and (a) relative elevation to timberline and (b) cost-distance from glacial refugia in mixed-ploidy species and aggregates, grouped by dispersal ability. Each point represents a taxon, colors indicate the direction and significance of the effect (brown = significant positive, green = significant negative, grey = non-significant). The dashed horizontal line denotes no effect (OR = 1). No group-level deviations from OR = 1 were statistically significant. Source data are provided as a Source Data file. Exact p- values from two-sided Wilcoxon signed-rank tests comparing taxon-specific ORs with OR = 1 are available in the corresponding Source Data file.

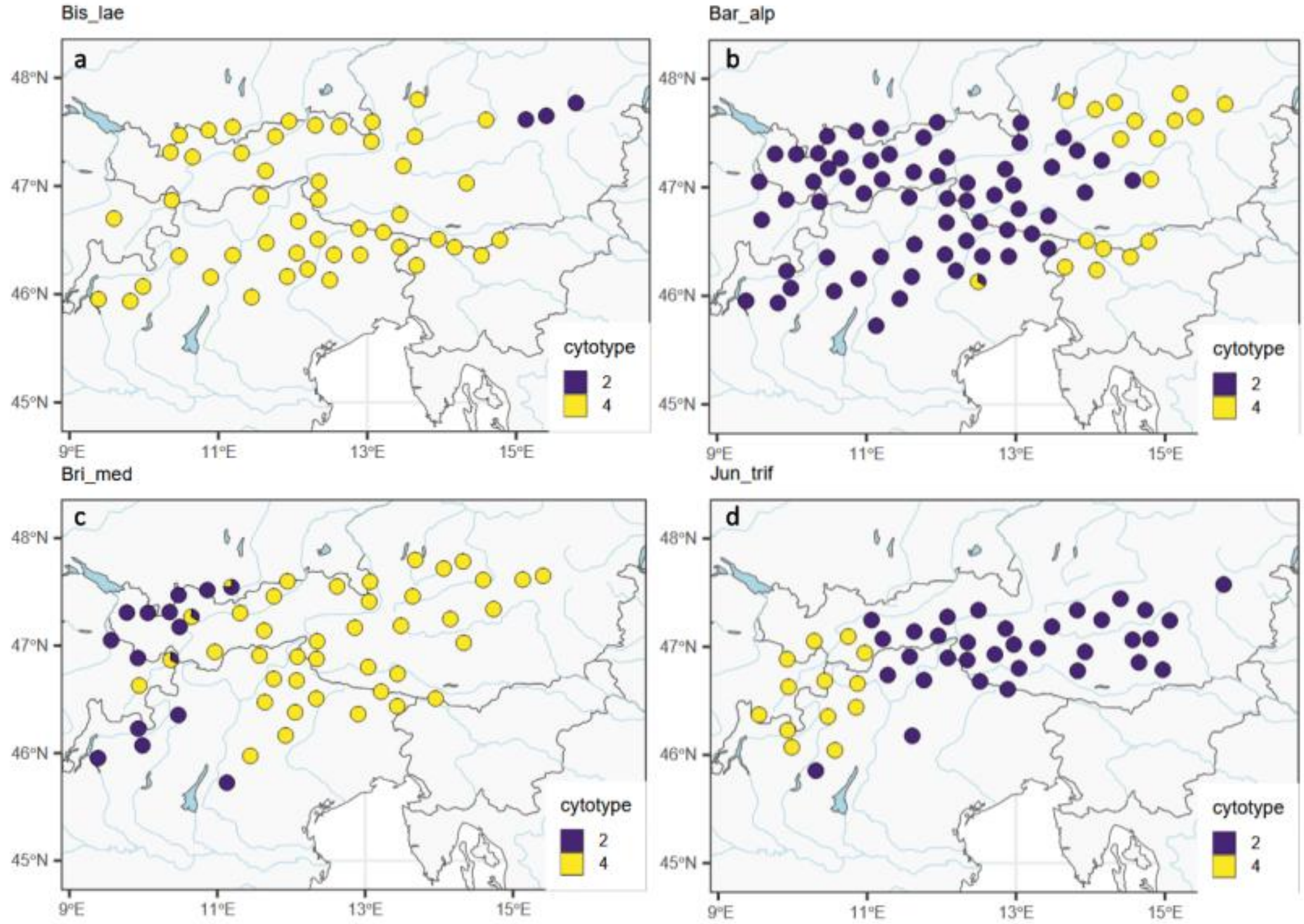

Supplementary Figure 9. Geographic distribution of cytotypes in mixed-ploidy species. Maps show the distribution of diploid (2*x*, purple) and tetraploid (4*x*, yellow) cytotypes across the Eastern Alps for four exemplary species. (a) *Biscutella laevigata* with diploids restricted to peripheral refugial areas, (b) *Bartsia alpina* with tetraploids restricted to peripheral refugial areas, (c) *Briza media*, and (d) *Juncus trifidus* with no strict refugial association but rather longitudinal separation. Country boundaries and other cartographic layers were obtained from Natural Earth[2]. Source data are provided as a Source Data file.

Supplementary References

1. Donnini, M., Marchesini, I. & Zucchini, A. A new geo-lithological map (Geo-LiM) for Central Europe (Germany, France, Switzerland, Austria, Slovenia, and Northern Italy). Zenodo https://doi.org/10.5281/ZENODO.3530257 (2019).
2. Natural Earth. Natural Earth: Free Vector and Raster Map Data. https://www.naturalearthdata.com/ (2026).
