# Supplementary material for: Polyploid frequency decreases with elevation but increases with distance to glacial refugia in the Eastern European Alps
Source: Nat Commun. 2026 Sep 12;17:10768. doi: 10.1038/s41467-026-77752-1 (PMC13661597; doi:10.1038/s41467-026-77752-1)
Supplement: Supplementary file 2 [file 41467_2026_77752_MOESM2_ESM.pdf]

<u>Description of Additional Supplementary Files</u>

Title: Supplementary Data 1. Sample information and associated variables.

Description: Each row represents a single sample and includes genus, species, updated taxonomic name, abbreviation, family, mountain, transect, country, province, mountain code, major geographic subunit of the Eastern Alps, Last Glacial Maximum refugium status, local treeline elevation, sampling belt, sampling-site identifier, geographic coordinates, sampling elevation, collector(s), collection date, cost-distances from putative refugia under three alternative assumptions regarding the permeability of unsuitable bedrock, taxon-level dispersal ability, ploidy information, inferred ploidy, grouping variables used for mixed-ploidy aggregate analyses, relative genome size(RGS), and the reference standard used for flow-cytometric measurements. Definitions of all variables are provided in the accompanying tab description.

Title: Supplementary Data 2. Results of binomial generalised linear mixed models (GLMMs)assessing associations between polyploidy and elevation or cost-distance variables

Description: All models use a logit link function. Statistical significance was assessed using two-sided Wald z-tests for fixed effects. No adjustment for multiple comparisons was applied. The column term indicates the model predictor; estimate gives the fixed-effect estimate on the log-odds scale, with associated std.error, statistic (z-value), p.value, and 95% confidence intervals (conf.low,conf.high). Response specifies the response variable used; random_structure denotes whether the model included a random intercept for transect or for both transect and family. Dist_var and model_name identify the cost-distance and polyploidy versions used, respectively. Model fit is reported as AIC, with explanatory power indicated by R2_marginal (variance explained by fixed effects) and R2_conditional(variance explained by fixed and random effects). Sample_size gives the number of observations included, and effect specifies the modeled effect type

Title: Supplementary Data 3. Results of binomial generalised linear models (GLMs) assessing associations between polyploidy and elevation, fitted separately for each genus.

Description: All models use a logit link function. Statistical significance was assessed using two-sided Wald z-tests for model coefficients. No adjustment for multiple comparisons was applied. The column term indicates the model predictor; estimate gives the fixed-effect estimate on the log-odds scale, with associated std.error, statistic (z-value), p.value, and 95% confidence intervals (conf.low, conf.high). Model fit is reported as AIC, and R2_marginalrepresents the variance explained by fixed effects. Sample_size gives the number of observations included.

Title: Supplementary Data 4. Results of binomial generalised linear models (GLMs) assessing associations between polyploidy and elevation, fitted separately for each mixed-ploidy taxon.

Description: All models use a logit link function. Statistical significance was assessed using two-sided Wald z-tests for model coefficients. No adjustment for multiple comparisons was applied. The column term indicates the model predictor; estimate gives the fixed-effect estimate on the log-odds scale, with associated std.error, statistic(z-value), p.value, and 95% confidence intervals (conf.low, conf.high). Model fit is reported as AIC, andR2_marginal represents the variance explained by fixed effects. Sample_size gives the number of observations included.
