# Supplementary material for: Polyploid frequency decreases with elevation but increases with distance to glacial refugia in the Eastern European Alps
Source: Nat Commun. 2026 Sep 12;17:10768. doi: 10.1038/s41467-026-77752-1 (PMC13661597; doi:10.1038/s41467-026-77752-1)
Supplement: Supplementary file 3 [file 41467_2026_77752_MOESM7_ESM.pdf]

nature portfolio

Corresponding author(s): Peter Schönswetter (peter.schoenswetter@uibk.ac.at)

Last updated by author(s): Jul 30, 2026

Reporting Summary

Nature Portfolio wishes to improve the reproducibility of the work that we publish. This form provides structure for consistency and transparency in reporting. For further information on Nature Portfolio policies, see our Editorial Policies and the Editorial Policy Checklist.

Statistics

For all statistical analyses, confirm that the following items are present in the figure legend, table legend, main text, or Methods section.

n/a	Confirmed	
☐	☒	The exact sample size (*n*) for each experimental group/condition, given as a discrete number and unit of measurement
☐	☒	A statement on whether measurements were taken from distinct samples or whether the same sample was measured repeatedly
☐	☒	The statistical test(s) used AND whether they are one- or two-sided<br>*Only common tests should be described solely by name; describe more complex techniques in the Methods section.*
☐	☒	A description of all covariates tested
☐	☒	A description of any assumptions or corrections, such as tests of normality and adjustment for multiple comparisons
☐	☒	A full description of the statistical parameters including central tendency (e.g. means) or other basic estimates (e.g. regression coefficient) AND variation (e.g. standard deviation) or associated estimates of uncertainty (e.g. confidence intervals)
☐	☒	For null hypothesis testing, the test statistic (e.g. *F*, *t*, *r*) with confidence intervals, effect sizes, degrees of freedom and *P* value noted<br>*Give P values as exact values whenever suitable.*
☒	☐	For Bayesian analysis, information on the choice of priors and Markov chain Monte Carlo settings
☐	☒	For hierarchical and complex designs, identification of the appropriate level for tests and full reporting of outcomes
☒	☐	Estimates of effect sizes (e.g. Cohen's *d*, Pearson's *r*), indicating how they were calculated

Our web collection on statistics for biologists contains articles on many of the points above.

Software and code

Policy information about availability of computer code

Data collection

no software used

Data analysis

All analyses were conducted in R (4.4.1). Provided annotated R scripts reproduce all statistical tests and figures presented in the manuscript and supplementary files. R Version: R >= 4.2.0 (analyses conducted in R 4.4.1)

Required R packages:
phytools_2.4-4
maps_3.4.2.1
phylolm_2.6.5
phyr_1.1.3
here_1.0.1
ape_5.8-1
broom.mixed 0.2.9.6
broom 1.0.7
MuMIn 1.48.11
performance 0.15.2
visreg 2.7.0
dotwhisker 0.8.3
sjlabelled 1.2.0
sjmisc 2.8.10
car 3.1-3

carData 3.0-5
DiagrammeR 1.0.11
ggeffects 1.7.2
sjPlot 2.8.16
piecewiseSEM 2.3.0
lme4 1.1-35.5
ggpubr 0.6.0
rstatix 0.7.2
janitor 2.2.0
patchwork 1.3.2
tidyterra 0.6.1
gdistance 1.6.4
Matrix 1.7-0
igraph 2.1.1
raster 3.6-30
sp 2.1-4
terra 1.7-83
sf 1.0-19
rio 1.2.3
lubridate 1.9.3
forcats 1.0.0
stringr 1.5.1
dplyr 1.1.4
purrr 1.0.2
readr 2.1.5
tidyr 1.3.1
tibble 3.2.1
ggplot2 4.0.2
tidyverse 2.0.0

For manuscripts utilizing custom algorithms or software that are central to the research but not yet described in published literature, software must be made available to editors and reviewers. We strongly encourage code deposition in a community repository (e.g. GitHub). See the Nature Portfolio guidelines for submitting code & software for further information.

Data

Policy information about availability of data

All manuscripts must include a data availability statement. This statement should provide the following information, where applicable:

- Accession codes, unique identifiers, or web links for publicly available datasets
- A description of any restrictions on data availability
- For clinical datasets or third party data, please ensure that the statement adheres to our policy

The complete dataset used for all analyses presented in this study is provided in Supplementary Data 1.

Research involving human participants, their data, or biological material

Policy information about studies with human participants or human data. See also policy information about sex, gender (identity/presentation), and sexual orientation and race, ethnicity and racism.

Reporting on sex and gender	not applicable
Reporting on race, ethnicity, or other socially relevant groupings	not applicable
Population characteristics	not applicable
Recruitment	not applicable
Ethics oversight	not applicable

Note that full information on the approval of the study protocol must also be provided in the manuscript.

Field-specific reporting

Please select the one below that is the best fit for your research. If you are not sure, read the appropriate sections before making your selection.

☐ Life sciences ☐ Behavioural & social sciences ☒ Ecological, evolutionary & environmental sciences

For a reference copy of the document with all sections, see nature.com/documents/nr-reporting-summary-flat.pdf

Ecological, evolutionary & environmental sciences study design

All studies must disclose on these points even when the disclosure is negative.

Study description	This study investigates how polyploid frequency varies along elevational gradients in a temperate mountain system and assesses the relative roles of current environmental conditions and historical range dynamics. Using a flora-wide dataset of georeferenced ploidy estimates from angiosperms sampled across the Eastern Alps, we examined patterns of polyploid distribution in relation to elevation, distance from glacial refugia, and species ecological traits.
Research sample	The research sample comprised 42,241 native angiosperm accessions representing 1,103 species across 68 plant families, collected from natural and semi-natural vegetation in the Eastern Alps (Switzerland, Germany, Austria, Italy, and Slovenia).
Sampling strategy	Sampling followed a standardized, systematic elevational-transect design. A total of 101 evenly spaced transects were centered on the timberline and spanned 1,100 m in elevation. Each transect comprised up to five equally spaced 100-m elevational belts separated by unsampled intervals; where summit height was insufficient, the uppermost belt was sampled on north-exposed summit slopes to capture the local species pool. Transects were preferentially positioned on south-facing slopes while avoiding northern exposures. Sampling was based on elevation and habitat availability and was not targeted toward specific species.
Data collection	Ploidy data were generated from silica-dried plant material collected along 101 elevational transects across the Eastern Alps using flow cytometry, yielding relative genome size estimates that were used to infer ploidy cytotypes. All collectors are listed in Supplementary Data 1. Details regarding flow cytometry and ploidy assignment are reported in Koutecký et al. (2026; https://www.preslia.cz/article/11593). Species ecological trait data, including light preference, substrate preference, and dispersal ability, were compiled from published ecological indicator value databases and trait syntheses (Karrer, 2024; Pignatti et al., 2005; Sádlo et al., 2018).Geological information was obtained from a published geo-lithological map of Central Europe (Geo-LiM; Donnini et al., 2019) and used to derive bedrock-specific conductance layers for cost-distance analyses. Spatial information on glacial refugia was taken from published sources.
Timing and spatial scale	All samples were collected on 101 elevational transects in the Eastern Alps in summer 2021 and 2022. The sampling period has no influence on the analytical approaches applied here, and was thus considered irrelevant.
Data exclusions	Samples from species with missing data on light preference and substrate preference were excluded from the relevant models and analyses. Samples lacking reliable ploidy estimates were excluded from all analyses.
Reproducibility	All findings are reproducible given the presented methodology and data.
Randomization	As our study is based on field-collected samples, no randomisation was necessary.
Blinding	Blinding was not necessary for this study

Did the study involve field work? ☒ Yes ☐ No

Field work, collection and transport

Field conditions	Field conditions were not considered to be relevant for the conducted sampling of plant tissue.
Location	All sampled localities and their coordinates are given in Supplementary Data 1.
Access & import/export	We obtained collecting permits from the relevant local authorities (permits are available upon request from the corresponding author)
Disturbance	Non-invasive sampling of leaf material for flow-cytometry from one individual per species per sampling belt.

Reporting for specific materials, systems and methods

We require information from authors about some types of materials, experimental systems and methods used in many studies. Here, indicate whether each material, system or method listed is relevant to your study. If you are not sure if a list item applies to your research, read the appropriate section before selecting a response.

Materials & experimental systems

n/a	Involved in the study
☒	☐ Antibodies
☒	☐ Eukaryotic cell lines
☒	☐ Palaeontology and archaeology
☒	☐ Animals and other organisms
☒	☐ Clinical data
☒	☐ Dual use research of concern
☐	☒ Plants

Methods

n/a	Involved in the study
☒	☐ ChIP-seq
☐	☒ Flow cytometry
☒	☐ MRI-based neuroimaging

Plants

Seed stocks: Leaf material was collected from the field for flow cytometry. Location information is provided in Supplementary Data 1.

Novel plant genotypes: not applicable

Authentication: not applicable

Flow Cytometry

Plots

Confirm that:

☐ The axis labels state the marker and fluorochrome used (e.g. CD4-FITC).

☐ The axis scales are clearly visible. Include numbers along axes only for bottom left plot of group (a 'group' is an analysis of identical markers).

☐ All plots are contour plots with outliers or pseudocolor plots.

☐ A numerical value for number of cells or percentage (with statistics) is provided.

Methodology

Sample preparation: Approximately 4–25 mm^2 of dried plant material and fresh internal standard were chopped in ice-cold Otto I buffer (0.1 M citric acid monohydrate, 0.5% Tween 20), filtered through a 42 µm nylon mesh, and stained with Otto II buffer (0.4 M $Na_2HPO_4 \cdot 12H_2O$, 0.2% 2-mercaptoethanol, 4 µg/ml DAPI). Samples were analysed after approximately 5 min of staining. The procedure followed the simplified two-step Otto protocol (protocol 1C) of Doležel et al. (2007), with modifications where necessary to obtain satisfactory histograms. Details of the standard protocol are provided in Doležel et al. (2007), and the specific modifications used here are described in Koutecký et al. (2026). https://www.preslia.cz/article/11593

Instrument: Sysmex CyFlow Space and Sysmex CyFlow Space equipped with a CyFlowSpace Autoloading Station 365-nm LED (Sysmex Partec)

Software: FloMax 2.9 (Sysmex Partec), flowPloidy package for R 4.4.2.

Cell population abundance: Cell sorting was not performed. Relative fluorescence intensity of approximately 3,000–5,000 particles was recorded per sample.

Gating strategy: Conventional FSC/SSC and positive/negative staining gates were not applicable. Flow-cytometric histograms were evaluated based on the sample and reference peaks, as described in Koutecký et al. (2026)

☐ Tick this box to confirm that a figure exemplifying the gating strategy is provided in the Supplementary Information.
