# Supplementary material for: Polyploid frequency decreases with elevation but increases with distance to glacial refugia in the Eastern European Alps
Source: Nat Commun. 2026 Sep 12;17:10768. doi: 10.1038/s41467-026-77752-1 (PMC13661597; doi:10.1038/s41467-026-77752-1)
Supplement: Supplementary file 4 [file 41467_2026_77752_MOESM8_ESM.pdf]

nature portfolio

# Peer Review File

# Polyploid frequency decreases with elevation but increases with distance to glacial refugia in the Eastern European Alps

Corresponding Author: Dr Peter Schönswetter

**This file contains all reviewer reports in order by version, followed by all author rebuttals in order by version.**

**Attachments originally included by the reviewers as part of their assessment can be found at the end of this file.**

Version 0:

Reviewer comments:

Reviewer #1

(Remarks to the Author)
See formatted version of the review in pdf form attached.

Review of NCOMMS-26-007639
Polyploid frequency decreases with elevation but increases with distance to glacial refugia in the Eastern European Alps, by Zeni et al.

Overall
The manuscript reports novel and important results based on extensive and thorough design of georeferenced and habitat-based sampling, appropriate statistical modelling and analyses. Main results, as reflected in the title, are (1) ploidy frequency decreases with elevation but (2) increases with distance to glacial refugia in the Eastern European alps. About these two main points, the authors explicitly describe all relevant sections clearly, from background, methodology to results and discussion. But, the third topic, that is "disentangling the effects of elevation and distance from refugia" is more complex and needs some adjustment, such as bringing point-conclusions up front. This third topic includes the analysis of interaction between them (elevation and distance) when accounting for potential effects of taxonomic composition, when sectioning across mixed-ploidy taxa, and when including functional traits, such as plant dispersal strategy and light requirement that may affect the relationship. The relationships are clearly complex, but most interesting.

Ploidy frequency decreases with elevation
Abstract, page 1, lines 21-23. The text reads "Based on these data, we find that polyploid frequency decreases with elevation, in contrast to expectations derived from global latitudinal distribution patterns."
Discussion, page 14, lines 352-355. The text reads "Our results clearly reject the hypothesis that ploidy level shall increase with elevation, challenging at least the generality of the notion on the positive association of polyploidy with harsh and unpredictable environments."
This conclusion has an unfairly negative connotation. Please consider rephrasing. The two scenarios should be compared subjectively, not putting one against another, i.e. as expected and the other as observed. When the observed does not agree with the expected, the expected is rejected. The two scenarios are completely different.
The polyploid frequency by elevation in the present study is a "regional" pattern and therefore should not be compared directly in a yes or no situation with the "global" latitudinal distribution patterns revealed by other studies or compilations, such as Rice et al. 2019 (Nat. Ecol. Evol. 3: 265-273), Brochmann et al. 2004 (Biol. J. Linn. Soc. 82: 521-536), Löve & Löve 1975 (Cytotaxonomical Atlas of the Arctic Flora. J. Cramer, Vaduz) and Stebbins 1971 (Chromosomal Evolution in Higher Plants, JW Arrowsmith Ltd, Bristol). There, the latitudinal range is very large, from equatorial tropics and subtropics, through temperate ecozones, to the arctic regions. Although it may be comparable between "arctic" and "alpine" in terms of low temperatures (cold), there are other parameters involved that make them different, e.g. day length. The elevation gradients over a mountain range could fall within a narrower latitudinal range. High latitudes like those in the arctics allow for short growing season, making flowering times of closely related species significantly overlap, promoting hybridisation, and in some cases leading to allopolyploidisation. High altitudes, on the other hand, comprises stable species diversity and taxonomical compositions, thus there is little space that could allow new hybridisation and colonisation. As rightly argued in the present manuscript, new polyploids may not have a chance to invade the already stable, cold-adapted vegetation that is

also tolerant to high altitude stresses. The vegetation of the upper alpine belts is likely to comprise both adapted polyploids and adapted diploids. The present study revealed low polyploid frequency in the upper belts in proportion to diploids, relative to polyploid frequencies at lower altitudes, i.e. below the timberline. But the polyploid frequencies at low altitudes are shown in the present study to have already been enriched due to the movement away from the refugia peripheral to the glaciated sites. The picture is more likely that the (new) polyploidy on the Eastern Alps does not move up the elevation gradients above the timberline. The polyploid frequency "does not increase" further up to the high elevation, rather than it is decreasing with elevation!. Stebbins (1971) mentioned (page 180) that "the percentage of polyploids in the flora of the high Alps, comprising the rocky refuges for plants which exist above the snow line, is no higher than in the plains and foothills below".

The arctic and the upper alpine are geographically different. While alpine mountain tops may be irregularly spaced and vegetatively discontinuous, not allowing plant migration without passing through the low elevations, there is a history of relevant connectivity throughout the arctic regions, not just across the latitudes, south-north, and also in the east-west direction (Walker et al. 2016. Circumpolar Arctic vegetation. Environ. Res. Lett. 11 055005). Numerous species have circumpolar distributions. Throughout the cycles of glaciation and deglaciation in the Pleistocene, the arctic landscape changed dramatically, often in the back-and-forth manner. At glacial maximum, the sea level fell, thus opening up more space and more connectivity of the unglaciated areas allowing plant migration, often leading to (allo-) polyploidisation. The Berring Landbridge is an example. Shortening distance between islands is another example. Refugia feeding the arctic were not only in the south of the icesheets but also from the east-west peripheries. Polyploid species did not just derive from diploid ancestors, but previously isolated polyploid species most probably hybridised with one another as well, through secondary contacts, hence climbing up the ploidy ladder. Looking at Svalbard as a model area (Brochmann et al. 2004), most (78.3%) of the 161 native taxa are polyploid, and the average ploidal level based on all species, including diploids, is close to hexaploid (5.4), and many species have very high ploidal levels. Further west into the Atlantic, we see polyploid-rich Icelandic flora (Löve & Löve 1956. A cytotaxonomical conspectus of the Icelandic flora. Acta Horti Gothobergensis 20: 65–290.), which is (most likely all) post-glacial, mostly coming in from northern Europe. Overall in the panarctic, the proportion of diploids versus polyploids is 13% vs. 87% in the glaciated region 'Atlantic' (including Svalbard and Iceland), whereas in the non-glaciated region 'Beringian' (including Siberia, Alaska and Yukon) the proportion is 31% vs. 69% (Brochmann et al. 2004). Estimated percentages of polyploidy for north-western Alaska (largely unglaciated) are 56-59% (Stebbins 1971).

Ploidy frequency is positively associated with distance from glacial refugia

The present study, based on the entire dataset of ca. 42,000 samples, revealed positive correlation between ploidy frequency and distance from glacial refugia, and across all transects. The further away from refugia the higher polyploid frequencies. This complies with other studies on this aspect, such as Brochmann et al. 2004. As suggested in this present paper, the results are consistent with the view that polyploids are more successful colonizers following deglaciation. However, when looking into the variation components, the frequency of polyploids was found to vary significantly across the three geographical subunits of the Eastern Alps. Mean frequency was higher in the Central Eastern Alps surrounding most of the previously glaciated area, than in the limestone areas covering the peripheral refugia in the north and the south. This is thought to be due to refugial persistence of diploid linages and differential postglacial expansion of polyploids into newly deglaciated terrains, whereby poor nutrients in the limestone calcareous bedrock (in the refugia at low elevations further away from the main glaciated area) presumably restricting the abundance of polyploids. If I understand it correctly. The authors further concluded that this is because polyploids are probably not adapted to marginal environments, in contrast to the general knowledge. Another question here.

Nevertheless, the authors stated that differences in taxonomic composition of the angiosperm floras across these three regions cannot be excluded as contributing to the observed spatial variation in polyploid frequency. In fact, the present study already showed different directions of polyploid frequencies among different taxonomic groups, be that by species or by genera, regarding either elevation or distance from refugia (Supplementary Figures 6-9). Some genera (or species) showed increasing polyploid frequency with elevation, some not increasing and others displayed no change, and this is the same for the distance from refugia. Supplementary Figure 11 shows examples of four different geographic distribution of diploid vs. tetraploid cytotypes (in mixed-ploidy species) across the Eastern Alps: (a) Biscutella laevigata with diploids restricted to peripheral refugial areas, (b) Bartsia alpina with tetraploids restricted to peripheral refugial areas, (c) Briza media and (d) Juncus trifidus with no strict refugial association but rather longitudinal separation. Clearly, taxonomic composition of the angiosperm floras across these different regions of the eastern Alps cannot be discarded as not contributing to the overall spatial variation in polyploid frequency.

Disentangling the effects of elevation and distance from refugia

The statistical modelling in the present study demonstrated a significant interaction between the effects of distance from refugia and elevation. This is to not surprising. The study region is a mountain range, therefore distance from refugia is mostly in the direction up the elevation. The main point in this study is the discovery that polyploid frequency increases away from the peripheral refugia (low elevation presumably) up to around the timberline and does not seem to make it higher up to the upper alpine zones. This is interesting. The study design included a fine-scale sampling not reported before. This opens up many biological questions to be answered by utilizing this very large dataset further. The reasons for polyploid frequency not increasing more to the upper zones can be many, some of which have been discussed in the present paper.

In the present study, certain plant functional traits were examined. In one scenario, the dataset was classified by plant dispersal ability (good vs. poor) and light preference (light-demanding vs. shade tolerance).

The results on page 7, lines 167-170: "The same elevational and spatial patterns as for the entire data set were revealed for poor dispersers (Supplementary Fig. 2), whereas no significant trend was found for good dispersers (Supplementary Fig. 3)".

The authors suggested that the dispersal mode modified the relationship between ploidy and elevational gradient, likely because efficient migrators maintained higher connectivity among populations during glacial cycles preventing the establishment of newly emerging polyploids at the leading edge of a species' distribution. But when looking at the figures, the reason why no significant trend was found among good dispersers, statistically, is because the variation at the upper

belts is so large and covering as much negative as positive trends. Therefore, the explanation given in the present study may not be the best. The good (fast) dispersers must be highly variable, for example, due to different means of dispersal among different plant taxonomic groups. Long-distance dispersal, such as by wind like in Betula that produces numerous, light winged seeds, can maintain good connectivity among populations as suggested. On the other hand, Salix, which comprises pioneer or early succession species well-adapted to disturbance, is mostly clonal (asexual). More than half of species in this genus is polyploid and the genus comprises numerous (newly formed) hybrids (Kaplan et al. 2019. Key to the flora of the Czech Republic, ISBN: 978-80-200-2660-6). Supplementary Figure 6 shows significantly positive relationship between polyploid frequency and elevation in Salix (page 14), the reverse of the main trend deriving from the whole dataset. When classifying species according to their light preference, clear difference emerged between shade-tolerant and light-demanding plants. Light-demanding taxa exhibited the same pattern as that across the entire dataset, regarding both distance and elevation. Among shade tolerant species, the distance-from-refugia effect persisted while the elevation effect was reversed (Supplementary Figures 4 and 5). Here the authors speculated (on page 12, lines 301-304) that "colonisation dynamics in forest species (mostly shade-tolerant) were more dynamic and hence more influential than in alpine species – which are predominantly light-demanding – as lower elevation species likely experienced stronger fragmentation during cold stages and longer periods of vicariance in smaller and fewer refugia." The authors suggested that the results here pointed to a stronger sorting of established polyploids vs. diploids in refugia in the course of postglacial colonisation dynamics. But when looking closely at supplementary Figure 4, the "reverse elevation effect" among shade-tolerant species is just at the border of statistical significance with p=0.05. Although the mean frequency of polyploid at the upper-most belt is higher than from lower belts, the variation at the uppermost belt is huge, and there, it was based on low number of shade-tolerant polyploid species. Visually, the pattern of polyploid frequency by elevation among shade-tolerant species is not solidly reverse as concluded. The polyploid frequency from both shade-tolerant and light-demanding species appears not to have increased further up above the timberline.

The conclusion stated on page 14, lines 344-346, is most appropriate and fair, that is "Our study bridges the gap between many single-species case studies, which yielded contradictory results on the one hand, and global studies addressing species-level patterns at coarser scales on the other h

Reviewer #2

(Remarks to the Author)
Dear authors, I congratulate you on a good paper!

I have carefully reviewed the manuscript, and left comments as track changes where pertinent. I report below the main concerns I found with the study, as well as less important issues or considerations that do not belong specifically in any one place in the manuscript.

I found the figures especially of very high quality, with legends and text large enough to be read comfortably, appropriate use of colour and a good information density as well as explanations in the captions.

======

MAJOR CONCERNS

How did you estimate timberline elevation?
The whole analysis rests on the relative elevation centered around the treeline, that conceptually makes sense but can also introduce difficulties.
The topic of treelines is somewhat fraught in alpine plant ecology, and especially in the Alps with their long history of human presence, much of current treelines are thought to be influenced by anthropogenic factors (see Körner's Alpine Treelines book and other more recent references). More details should be provided on how it was determined, which considerations were taken into account, and potential sources of bias.

Distance analysis
The conductance layer you calculated seems based on available geological map resolution, not necessarily on biologically meaningful measures. Surely, good dispersers can easily cross 200m in one generation? Is the distance cost calculated step-wise for each cell? Can you comment on the impact on your results if a species can "skip cells" by dispersing to cells that are not adjacent?

Light requirements
I did not understand why you looked at light / shade preference for elevation and distance analyses? Why would these trait affect species' dispersal and/or elevation distribution? If there is a hypotheses, this needs to be clearly laid out. If not clear, it's best to remove as it is not important to understand the results presented.

Polyploid frequency
Polyploid frequency calculated as polyploid samples / number of taxa? Or as polyploid samples / non-polyploid samples? (see my comments in the methods section) Phrased as it is in the methods, it seems like if you have e.g. 10 polyploid samples in a pool of 50 species, this variable would be the same value whether the polyploids samples are from 10 different species or all from the same species!

Phylogenetic correction
You used plant family as a random factor to correct for phylogeny, which is a rather coarse approach. This also results in a multitude of partial models, which lose explanatory power compared to a bigger model. See my comments in the methods and results sections.
An alternative approach would be to use a phylogenetically corrected GLM, but I am keenly aware of scope creep, and this paper is already substantial enough on its own, so I leave this to the authors' discretion. As I point out in some comments in the manuscript, there is potential for a follow-up study that looks more closely at the influence of phylogeny.

=====

MINOR CONCERNS

There is a tendency to use the passive voice, which sometimes results in awkward sentence structures; I pointed out some examples, but when in doubt switching to active voice often improves clarity.

The plant sampling strategy within the transects is not explained very well, more details are needed.

Anthropogenic factors / land use can have shaped metapopulation dynamics (and thus potential polyploid adaptive landscapes) at the edges of the Alps, where population density is higher, increasing habitats with disturbance where polyploids are thought to be more frequent.

I quite liked the path analysis, it nicely shows the reasoning behind the analysis (and in my opinion shows that there is no clear hypothesis behind light requirement inclusion as an explanatory variable) maybe one or two panels could get promoted to the main text. It needs to be explained what is the hypothesis behind forest (i.e. light requirement) having an effect on polyploidy, and perhaps redrawn to have larger text and confidence intervals.

Supplementary figures 6 to 9: I have to be honest, I found hard to parse the information in this panels... It might be helpful to present only (or at least prominently) a select few cases to illustrate the general pattern in the flora.
From what I gather, you plotted here the odds ratio for each taxon that had multiple cytotypes, but why are they split in multiple figures? I found this very confusing; also sometimes the abbreviation is used and sometimes the taxon name.

Version 1:

Reviewer comments:

Reviewer #1

(Remarks to the Author)
The revision following my comments and suggestions is satisfactory, fair and reasonable.

(Remarks on code availability)

Reviewer #2

(Remarks to the Author)
Dear authors,
many thanks for the revised version of your work.

In general I am happy with the revisions and do not ask any more corrections; the work is ready for publication in my opinion. Below a few comments, to conclude the conversation.

I re-read the manuscript, found the flow improved, and I think the narrative is much enhanced by the removal of the light-demanding VS share-tolerant part (not that it is not interesting, but it did not add much to the already solid story in this paper). In general the clarity is also improved, especially in some sections of the methods.

The added phylogenetic correction supports your conclusions, as expected, and I think will help convincing readers; I am satisfied with how it was performed, and hopefully it was not too much work to implement.

On the supplementary figures, I see your point, and I can indeed picture dedicated botanists checking out specific species trends in the supplementary material.

On the nunataks, this sounds like an ongoing discussion, I perhaps wrongly assumed it was settled; in any case the text is not present in the manuscript anymore, but good to know the context nonetheless.

For the altered habitats point, I meant to perhaps clarify with the details that the authors' indeed provided in their response; upon re-reading the passage it did not jump out to me anymore, perhaps I was less attentive on first pass.

Finally, regarding the sampling, I clearly underestimated the authors' mettle, and applaud the effort in collecting ALL

angiosperms along their gradients!

I wish you a smooth rest of the publication process, viel Glück!

(Remarks on code availability)

**Open Access** This Peer Review File is licensed under a Creative Commons Attribution 4.0 International License, which permits use, sharing, adaptation, distribution and reproduction in any medium or format, as long as you give appropriate credit to the original author(s) and the source, provide a link to the Creative Commons license, and indicate if changes were made.
In cases where reviewers are anonymous, credit should be given to 'Anonymous Referee' and the source.
The images or other third party material in this Peer Review File are included in the article's Creative Commons license, unless indicated otherwise in a credit line to the material. If material is not included in the article's Creative Commons license and your intended use is not permitted by statutory regulation or exceeds the permitted use, you will need to obtain permission directly from the copyright holder.
To view a copy of this license, visit https://creativecommons.org/licenses/by/4.0/

Reviewer #1 (Remarks to the Author):

**Review of NCOMMS-26-007639**

Polyploid frequency decreases with elevation but increases with distance to glacial refugia in the Eastern European Alps, by Zeni et al.

**Overall**

The manuscript reports novel and important results based on extensive and thorough design of georeferenced and habitat-based sampling, appropriate statistical modelling and analyses. Main results, as reflected in the title, are (1) ploidy frequency decreases with elevation but (2) increases with distance to glacial refugia in the Eastern European alps. About these two main points, the authors explicitly describe all relevant sections clearly, from background, methodology to results and discussion. But, the third topic, that is "disentangling the effects of elevation and distance from refugia" is more complex and needs some adjustment, such as bringing point-conclusions up front. This third topic includes the analysis of interaction between them (elevation and distance) when accounting for potential effects of taxonomic composition, when sectioning across mixed-ploidy taxa, and when including functional traits, such as plant dispersal strategy and light requirement that may affect the relationship. The relationships are clearly complex, but most interesting.

**Ploidy frequency decreases with elevation**

Abstract, page 1, lines 21-23. The text reads "*Based on these data, we find that polyploid frequency decreases with elevation, in contrast to expectations derived from global latitudinal distribution patterns*."

>> See also our next response. Rephrased to "Based on these data, we find that on a regional level polyploid frequency decreases with elevation, in contrast to expectations derived from global latitudinal distribution patterns", lines 21–22.

---

Discussion, page 14, lines 352-355. The text reads "*Our results <u>clearly reject the hypothesis</u> that ploidy level shall increase with elevation, challenging at least the generality of the notion on the positive association of polyploidy with harsh and unpredictable environments.*"

This conclusion has an unfairly negative connotation. Please consider rephrasing. The two scenarios should be compared subjectively, not putting one against another, i.e. as expected and the other as observed. When the observed does not agree with the expected, the expected is rejected. The two scenarios are completely different.

The polyploid frequency by elevation in the present study is a "regional" pattern and therefore should not be compared directly in a yes or no situation with the "global" latitudinal distribution patterns revealed by other studies or compilations, such as Rice et al. 2019 (Nat. Ecol. Evol. 3: 265-273), Brochmann et al. 2004 (Biol. J. Linn. Soc. 82: 521-536), Löve & Löve 1975 (Cytotaxonomical Atlas of the Arctic Flora. J. Cramer, Vaduz) and Stebbins 1971 (Chromosomal Evolution in Higher Plants, JW Arrowsmith Ltd, Bristol). There, the latitudinal range is very large,

from equatorial tropics and subtropics, through temperate ecozones, to the arctic regions. Although it may be comparable between "arctic" and "alpine" in terms of low temperatures (cold), there are other parameters involved that make them different, e.g. day length. The elevation gradients over a mountain range could fall within a narrower latitudinal range. High latitudes like those in the arctics allow for short growing season, making flowering times of closely related species significantly overlap, promoting hybridisation, and in some cases leading to allopolyploidisation. High altitudes, on the other hand, comprises stable species diversity and taxonomical compositions, thus there is little space that could allow new hybridisation and colonisation. As rightly argued in the present manuscript, new polyploids may not have a chance to invade the already stable, cold-adapted vegetation that is also tolerant to high altitude stresses. The vegetation of the upper alpine belts is likely to comprise both adapted polyploids and adapted diploids. The present study revealed low polyploid frequency in the upper belts in proportion to diploids, relative to polyploid frequencies at lower altitudes, i.e. below the timberline. But the polyploid frequencies at low altitudes are shown in the present study to have already been enriched due to the movement away from the refugia peripheral to the glaciated sites. The picture is more likely that the (new) polyploidy on the Eastern Alps does not move up the elevation gradients above the timberline. The polyploid frequency "does not increase" further up to the high elevation, rather than it is decreasing with elevation!. Stebbins (1971) mentioned (page 180) that "*the percentage of polyploids in the flora of the high Alps, comprising the rocky refuges for plants which exist above the snow line, is no higher than in the plains and foothills below*".

>> We fully agree with Reviewer 1 that the global, latitudinal pattern and the regional, altitudinal pattern may be shaped by entirely different drivers and that a direct comparison is not admissible. However, high-altitude and high-latitude habitats indeed have striking similarities, for instance in the harshness of the climatic conditions they provide. This is what we were referring to in our comparison. We rephrased the text, it now reads (lines 355–356): "Our results clearly show that on a regional level ploidy level does not increase with elevation, challenging at least the generality of the notion on the positive association of polyploidy with harsh and unpredictable environments."

The arctic and the upper alpine are geographically different. While alpine mountain tops may be irregularly spaced and vegetatively discontinuous, not allowing plant migration without passing through the low elevations, there is a history of relevant connectivity throughout the arctic regions, not just across the latitudes, south-north, and also in the east-west direction (Walker et al. 2016. Circumpolar Arctic vegetation. Environ. Res. Lett. 11 055005). Numerous species have circumpolar distributions. Throughout the cycles of glaciation and deglaciation in the Pleistocene, the arctic landscape changed dramatically, often in the back-and-forth manner. At glacial maximum, the sea level fell, thus opening up more space and more connectivity of the unglaciated areas allowing plant migration, often leading to (allo-) polyploidisation. The Berring Landbridge is an example. Shortening distance between islands is another example. Refugia feeding the arctic were not only in the south of the icesheets but also from the east-west peripheries. Polyploid species did not just derive from diploid ancestors, but previously isolated polyploid species most probably hybridised with one another as well, through secondary contacts, hence climbing up the ploidy ladder. Looking at Svalbard as a model area (Brochmann et al. 2004), most (78.3%) of the 161 native taxa are polyploid, and the average ploidal level based on all species, including diploids, is close to hexaploid (5.4), and many species have very high ploidal levels. Further west into the Atlantic, we see polyploid-rich Icelandic flora (Löve & Löve 1956. A cytotaxonomical conspectus of the Icelandic flora. Acta Horti Gothobergensis 20: 65–290.), which is (most likely all) post-glacial, mostly coming in from northern Europe. Overall in the panarctic, the proportion of diploids versus polyploids is 13% vs. 87% in the glaciated region 'Atlantic' (including Svalbard and Iceland), whereas in the non-glaciated region 'Beringian' (including Siberia, Alaska and Yukon) the proportion is 31% vs. 69% (Brochmann et al. 2004). Estimated percentages of polyploidy for north-western Alaska (largely unglaciated) are 56-59% (Stebbins 1971).

>> We are most grateful to Reviewer 1 for sharing his insightful thoughts on the arctic flora with us.

**Ploidy frequency is positively associated with distance from glacial refugia**

The present study, based on the entire dataset of ca. 42,000 samples, revealed positive correlation between ploidy frequency and distance from glacial refugia, and across all transects. The further away from refugia the higher polyploid frequencies. This complies with other studies on this aspect, such as Brochmann et al. 2004. As suggested in this present paper, the results are consistent with the view that polyploids are more successful colonizers following deglaciation.

However, when looking into the variation components, the frequency of polyploids was found to vary significantly across the three geographical subunits of the Eastern Alps. Mean frequency was higher in the Central Eastern Alps surrounding most of the previously glaciated area, than in the limestone areas covering the peripheral refugia in the north and the south. This is thought to be due to refugial persistence of diploid linages and differential postglacial expansion of polyploids into newly deglaciated terrains, whereby poor nutrients in the limestone calcareous bedrock (in the refugia at low elevations further away from the main glaciated area) presumably restricting the abundance of polyploids. If I understand it correctly. The authors further concluded that this is because polyploids are probably not adapted to marginal environments, in contrast to the general knowledge. Another question here.

>> The situation in the Eastern Alps is slightly more complex than summarised by Reviewer 1. The Central Alps were definitely more strongly glaciated than the Southern Limestone Alps, but the Northern Limestone Alps were – in spite of being peripheral – strongly glaciated except for their eastern-most part. Therefore, short migration distances were expected for the Southern Limestone Alps and rather long ones for the Central Eastern Alps and the Northern Limestone Alps. Further, we did not really "conclude[d] that … polyploids are probably not adapted to marginal environments" as we do not have the data backing this statement. What we did write is that other studies have shown that polyploids may be less abundant on substrates with low nutrient availability such as soils on calcareous bedrock (lines 296–300). No changes to the text were done.

Nevertheless, the authors stated that differences in taxonomic composition of the angiosperm floras across these three regions cannot be excluded as contributing to the observed spatial variation in polyploid frequency. In fact, the present study already showed different directions of polyploid frequencies among different taxonomic groups, be that by species or by genera, regarding either elevation or distance from refugia (Supplementary Figures 6-9). Some genera (or species) showed increasing polyploid frequency with elevation, some not increasing and others displayed no change, and this is the same for the distance from refugia. Supplementary Figure 11 shows examples of four different geographic distribution of diploid vs. tetraploid cytotypes (in mixed-ploidy species) across the Eastern Alps: (a) *Biscutella laevigata* with diploids restricted to peripheral refugial areas, (b) *Bartsia alpina* with tetraploids restricted to peripheral refugial areas, (c) *Briza media* and (d) *Juncus trifidus* with no strict refugial association but rather longitudinal separation. Clearly, taxonomic composition of the angiosperm floras across these different regions of the eastern Alps cannot be discarded as not contributing to the overall spatial variation in polyploid frequency.

>> We fully agree with this summary by Reviewer 1.

**Disentangling the effects of elevation and distance from refugia**

The statistical modelling in the present study demonstrated a significant interaction between the effects of distance from refugia and elevation. This is to not surprising. The study region is a mountain range, therefore distance from refugia is mostly in the direction up the elevation. The main point in this study is the discovery that polyploid frequency increases away from the peripheral refugia (low elevation presumably) up to around the timberline and does not seem to make it higher up to the upper alpine zones. This is interesting. The study design included a finescale sampling not reported before. This opens up many biological questions to be answered by

utilizing this very large dataset further. The reasons for polyploid frequency not increasing more to the upper zones can be many, some of which have been discussed in the present paper.
In the present study, certain plant functional traits were examined. In one scenario, the dataset was classified by plant dispersal ability (good vs. poor) and light preference (light-demanding vs. shade tolerance).
>> Again, this is an excellent summary and we agree that the data set can likely be used for further exploring the underlying causes of particularities of the distribution of polyploids, which are beyond the scope of the present MS. For instance, we explicitly state in the concluding paragraph of the Discussion (lines 357–362) that "our results suggest that the relationships between polyploidy and elevation are not an intrinsic ecological or physiological consequence of WGD that would act as a constraint or provide adaptive benefit at particular elevations, but largely reflect species' idiosyncrasies as well as differences in taxonomic composition, ecological strategies, and historical range dynamics of the species occupying different elevational belts".

However, we do not agree with Reviewer 1 that "distance from refugia is mostly in the direction up the elevation" as the Eastern Alps are strongly shaped by east-west running valleys, which are clearly visible in Fig. 1. In the same line, the world-famous landscape of the Dolomites, which are ancient coral reefs protruding from a gentle rolling surface, clearly contradict the cited statement.

The results on page 7, lines 167-170: "*The same elevational and spatial patterns as for the entire data set were revealed for poor dispersers (Supplementary Fig. 2), whereas no significant trend was found for good dispersers (Supplementary Fig. 3)*". The authors suggested that the dispersal mode modified the relationship between ploidy and elevational gradient, likely because efficient migrators maintained higher connectivity among populations during glacial cycles preventing the establishment of newly emerging polyploids at the leading edge of a species' distribution. But when looking at the figures, the reason why no significant trend was found among good dispersers, statistically, is because the variation at the upper belts is so large and covering as much negative as positive trends. Therefore, the explanation given in the present study may not be the best. The good (fast) dispersers must be highly variable, for example, due to different means of dispersal among different plant taxonomic groups. Long-distance dispersal, such as by wind like in *Betula* that produces numerous, light winged seeds, can maintain good connectivity among populations as suggested. On the other hand, *Salix*, which comprises pioneer or early succession species well-adapted to disturbance, is mostly clonal (asexual). More than half of species in this genus is polyploid and the genus comprises numerous (newly formed) hybrids (Kaplan et al. 2019. Key to the flora of the Czech Republic, ISBN: 978-80-200-2660-6). Supplementary Figure 6 shows significantly positive relationship between polyploid frequency and elevation in *Salix* (page 14), the reverse of the main trend deriving from the whole dataset.
>> In the revised text, we acknowledge the large variation in the frequency of polyploids above the treeline in good dispersers (lines 171–174). We also agree that this variability might reflect heterogeneity in dispersal strategies within the "good dispersers" category as different mechanisms might lead to contrasting patterns of connectivity and polyploid establishment. However, disentangling these mechanisms – although highly interesting – is clearly beyond the scope of our study. In addition, our framework does not account for reproductive strategies such as clonal growth, which may strongly influence polyploid establishment in some taxa. These processes could decouple polyploid frequency from dispersal-mediated colonization dynamics and contribute to the high variability observed. Regarding the example of *Salix*, we agree that it shows a pattern opposite from the overall dataset but we disagree that the *Salix* species in the study area are mostly clonal, only a few species (*S. herbacea*, *S. reticulata*) have underground runners. In any event, our analyses focus on average patterns (not capturing lineage-specific processes) and genus-specific dynamics may deviate from the general trend.

When classifying species according to their light preference, clear difference emerged between

shade-tolerant and light-demanding plants. Light-demanding taxa exhibited the same pattern as that across the entire dataset, regarding both distance and elevation. Among shade tolerant species, the distance-from-refugia effect persisted while the elevation effect was reversed (Supplementary Figures 4 and 5). Here the authors speculated (on page 12, lines 301-304) that "*colonisation dynamics in forest species (mostly shade-tolerant) were more dynamic and hence more influential than in alpine species – which are predominantly light-demanding – as lower elevation species likely experienced stronger fragmentation during cold stages and longer periods of vicariance in smaller and fewer refugia*." The authors suggested that the results here pointed to a stronger sorting of established polyploids vs. diploids in refugia in the course of postglacial colonisation dynamics. But when looking closely at supplementary Figure 4, the "reverse elevation effect" among shade-tolerant species is just at the border of statistical significance with p=0.05. Although the mean frequency of polyploid at the upper-most belt is higher than from lower belts, the variation at the uppermost belt is huge, and there, it was based on low number of shade-tolerant polyploid species. Visually, the pattern of polyploid frequency by elevation among shade-tolerant species is not solidly reverse as concluded. The polyploid frequency from both shade-tolerant and light-demanding species appears not to have increased further up above the timberline.
>> Thank you for pointing this out. We removed the analyses based on light / shade preference from the manuscript, as they were not central to the main conclusion of the study.

The **conclusion** stated on page 14, lines 344-346, is most appropriate and fair, that is "*Our study bridges the gap between many single-species case studies, which yielded contradictory results on the one hand, and global studies addressing species-level patterns at coarser scales on the other hand.*"
>> Once again, we are most grateful to Reviewer 1 for his insightful comments and the appreciation of our work.

Reviewer #2 (Remarks to the Author):

Dear authors, I congratulate you on a good paper!

>> We are grateful to Reviewer 2 for his very positive feedback!

I have carefully reviewed the manuscript, and left comments as track changes where pertinent. I report below the main concerns I found with the study, as well as less important issues or considerations that do not belong specifically in any one place in the manuscript.

>> We revised the text accordingly, incorporating changes where appropriate. Below, we provide our responses to comments where we were not able to fully implement the suggested revisions, indicating the corresponding line number for clarity.

I found the figures especially of very high quality, with legends and text large enough to be read comfortably, appropriate use of colour and a good information density as well as explanations in the captions.

======

MAJOR CONCERNS

How did you estimate timberline elevation?

The whole analysis rests on the relative elevation centered around the treeline, that conceptually makes sense but can also introduce difficulties.

The topic of treelines is somewhat fraught in alpine plant ecology, and especially in the Alps with their long history of human presence, much of current treelines are thought to be influenced by anthropogenic factors (see Körner's Alpine Treelines book and other more recent references). More details should be provided on how it was determined, which considerations were taken into account, and potential sources of bias.

>> Thank you for bringing up this important point. We added more details to the Methods as we agree that the description was too short and too general (lines 370–373). The position of the timberline was identified using Google Earth. For each summit (or a nearby summit if image quality was poor), we selected the highest-elevation occurrences of groups of trees within a 5 km radius. We chose this approach to counterbalance anthropogenic depression of the timberline by including areas likely unaffected. We initially considered existing modelled treeline datasets, which proved unsuitable, particularly on limestone substrates, where *Pinus mugo* krummholz was apparently misclassified as forest, leading to overestimation of treeline position. Our approach focuses on the actual timberline based on visual interpretation rather than climatic models. Overall, "our" treeline position exactly follows the expectations in the relevant literature with highest treelines in the interior, most continental parts of the Eastern Alps and much lower treelines along the oceanic margin of the Alps, especially the northern margin (the southern margin is warmer with slightly higher treelines). We acknowledge potential bias including subjective interpretation, variable image resolution and quality, and the possible exclusion of individuals that might lead to a more conservative estimate of timberline elevation. We clarified the procedure in the Methods section.

Distance analysis

The conductance layer you calculated seems based on available geological map resolution, not necessarily on biologically meaningful measures. Surely, good dispersers can easily cross 200m in one generation? Is the distance cost calculated step-wise for each cell? Can you comment on the impact on your results if a species can "skip cells" by dispersing to cells that are not adjacent?

>> Movement is modelled step-wise from cell to adjacent cell, we did not allow for skipping to non-adjacent cells. While some species might disperse longer distances in a single generation, our approach provides a relative measure of connectivity that accounts for variation in landscape resistance more realistically than straight-line distances. We acknowledge that this is a simplification across the species pool and does not incorporate species-specific dispersal traits. Allowing "skipping" could reduce distance estimates for good dispersers, but we found that these species show no variation in polyploid frequency with distance (Supplementary Fig. 3), suggesting that skipping would not affect the observed patterns.

Light requirements

I did not understand why you looked at light / shade preference for elevation and distance analyses? Why would these trait affect species' dispersal and/or elevation distribution? If there is a hypotheses, this needs to be clearly laid out. If not clear, it's best to remove as it is not important to understand the results presented.

>> Thank you for pointing this out. We removed the analyses based on light / shade preference from the manuscript, as they were not central to the main conclusion of the study.

Polyploid frequency

Polyploid frequency calculated as polyploid samples / number of taxa? Or as polyploid samples / non-polyploid samples? (see my comments in the methods section) Phrased as it is in the methods, it seems like if you have e.g. 10 polyploid samples in a pool of 50 species, this variable would be the

same value whether the polyploids samples are from 10 different species or all from the same species!

>> For the analysis presented in Fig 3a, polyploid frequency on each mountain was modelled as the number of polyploid cytotypes divided by the total number of cytotypes recorded on that mountain, counting each cytotype only once. We revised the Methods section to improve clarity (lines 457–459).

Phylogenetic correction

You used plant family as a random factor to correct for phylogeny, which is a rather coarse approach. This also results in a multitude of partial models, which lose explanatory power compared to a bigger model. See my comments in the methods and results sections.

An alternative approach would be to use a phylogenetically corrected GLM, but I am keenly aware of scope creep, and this paper is already substantial enough on its own, so I leave this to the authors' discretion. As I point out in some comments in the manuscript, there is potential for a follow-up study that looks more closely at the influence of phylogeny.

>> We thank the reviewer for pointing out the limitations of using plant family as a random effect to account for phylogenetic non-independence. To complement this approach we performed the analyses suggested (estimate phylogenetic signal of ploidy and phylogenetically corrected GLM) and added them to the manuscript (lines 202–209, lines 511–524). These complementary analyses supported our conclusions while explicitly accounting for shared ancestry. We agree that more detailed exploration of phylogenetic influence could be the basis of follow up studies.

=====

MINOR CONCERNS

There is a tendency to use the passive voice, which sometimes results in awkward sentence structures; I pointed out some examples, but when in doubt switching to active voice often improves clarity.

>> This seems to be a matter of personal writing style. However, the text has been revised according to the reviewer's suggestions.

The plant sampling strategy within the transects is not explained very well, more details are needed.

>> We have revised the text accordingly to clarify the sampling strategy (e.g. line 381).

Anthropogenic factors / land use can have shaped metapopulation dynamics (and thus potential polyploid adaptive landscapes) at the edges of the Alps, where population density is higher, increasing habitats with disturbance where polyploids are thought to be more frequent.

>> We thank the reviewer for this interesting suggestion. While anthropogenic factors may indeed influence metapopulation dynamics (which are beyond the scope of our study), it is not true that anthropogenic pressure is higher at the periphery of the Alps. In contrast, large areas in the southern and especially the northern Prealps are only thinly populated (due to the harsh climate, etc.), inner-Alpine valleys are much more densely populated, which confers a strong anthropogenic pressure. Moreover, some of the thinly populated areas overlap strongly with potential peripheral refugia.

I quite liked the path analysis, it nicely shows the reasoning behind the analysis (and in my opinion shows that there is no clear hypothesis behind light requirement inclusion as an explanatory variable) maybe one or two panels could get promoted to the main text. It needs to be explained

what is the hypothesis behind forest (i.e. light requirement) having an effect on polyploidy, and perhaps redrawn to have larger text and confidence intervals.

>> We redrew the figure with larger text to improve readability and added statistical significance to the path coefficients. One representative panel was moved to the main text (now Fig. 5, line 901), the original figure was removed to the Supplement.

Supplementary figures 6 to 9: I have to be honest, I found hard to parse the information in this panels... It might be helpful to present only (or at least prominently) a select few cases to illustrate the general pattern in the flora.

From what I gather, you plotted here the odds ratio for each taxon that had multiple cytotypes, but why are they split in multiple figures? I found this very confusing; also sometimes the abbreviation is used and sometimes the taxon name.

>> We revised the plot titles to consistently use the full taxon names instead of abbreviations to improve clarity. The figures are separated because they represent two subsets (mixed-ploidy aggregates and genera) and two different explanatory variables. Regarding the suggestion to present only a subset of cases, our aim with these figures is to show that the pattern is not consistent across species; for this reason, we prefer to keep them as they are. We are fully aware that these figures are only relevant for a small fraction of the readers but based on our knowledge of Eastern Alpine botanists we anticipate that they will appreciate these Supplementary figures.

======

FURTHER COMMENTS

94: You have not, so far, described why polyploids might hybridise more frequently than diploids; consider adding a sentence about it here, along with a reference, e.g.
Petit, C., Bretagnolle, F., & Felber, F. (1999). Evolutionary consequences of diploid-polyploid hybrid zones in wild species. Trends in Ecology & Evolution, 14(8), 306–311. https://doi.org/10.1016/S0169-5347(99)01608-0

>> We do not think that in the context of our study it is important whether polyploids hybridise more often (with other polyploids) than diploids do. It is clear that polyploid hybrids retain fertility more easily due to the possibility of chromosome pairing in the meiosis, but we do not think that this has any impact on our results.

113-118: These sentences very clearly do not belong here. You can have something like this at beginning of the discussion to recap your questions and results.

>> These sentences were added to fit the journal style.

148-150: You need to provide background for this statement, ideally in the introduction but adding a sentence and a reference here would be acceptable too. For example, the excellent Wood et al 2009 - https://doi.org/10.1073/pnas.0811575106

>> We added this sentence with the suggested reference to the Results: "Many polyploid lineages are thought to arise frequently but persist only transiently, meaning recently formed polyploids may not yet show consistent evolutionary or ecological patterns" (lines 149–151)

157: What if species could disperse longer distances? See my other comments on cell size

AND

430-434: It is not clear to me if this calculation is step-wise, i.e. with the transition of cells dependent on the result of adjacent cells, or not; see my comment on the main review text about "skipping cells"

>> See reply above (Distance analysis)

170-171: It's entirely unclear to me why these traits would have an influence… What's the hypotheses here? It should have been presented already in the last part of the introduction

>> See reply above (Light requirements)

190-191: This is a rather coarse correction for phylogeny… Not all families are "equidistant" in phylogenetic terms, and a trait similarity in distantly related families should weight more in your model than one in closely related families. See my comment on this in methods

AND

508-510: This is a rather odd approach… What you are asking with this is, really: "does phylogeny affect the distribution of polyploids along elevation?" The typical way of doing this would be a phylogenetically corrected GLM, for example using phyloLM. Alternatively, you could look at the phylogenetic signal (Pagel's lambda) for the trait (polyploidy), for example with function phylosig from phytools.

>> See above (Phylogenetic correction)

220: I think one or two panels of this supplementary fig could be redrawn a bit (bigger text, shorter arrows, * for confidence) and included in the main text. I also think it shows that there is no clear hypothesis with using light requirements as a factor, and this should be either explained better or removed from all analysis.

AND

250: If you are referring to a supplementary figure in the final paragraph of your discussion, it's a telltale sing that it belongs in the main text!

>> see reply above on Path analysis

243-244: There are many examples of the opposite as well, with polyploids flowering earlier and being shorter than diploids. It really is a mixed bag, you should present it as such, and support with 1-2 recent references each side of the argument

>> We agree with Reviewer 2. The text was changed, it now reads (lines 251–253): "However, these patterns are not consistent across taxa. In some taxa polyploids flower earlier than diploids[52,53], and size differences between cytotypes are likewise variable[52]"

306: This terminology is not consistent with Holderegger & Thiel-Egenter 2009, and in fact I am slightly confused by its use still. Just "nunatak" is better in my opinion

>> This comment reflects a long discussion between Rolf Holderegger and myself (P. Schönswetter), which boils down to the question whether a peak surrounded by ice but situated close to the margin of a mountain range is a nunatak (opinion of P. Schönswetter, reflecting the definition of nunatak on Wikipedia: https://en.wikipedia.org/wiki/Nunatak), even if it is a peripheral one, or rather a peripheral refugium (opinion of R. Holderegger) even if it entirely falls into the definition of nunatak. We would prefer the wording as it is as in our opinion it is more precise. However, this text was deleted from the manuscript after excluding the separate analyses of light-demanding species.

374-375: Surely you did not sample ALL angiosperms in these habitats; presumably you had a taxa list that you tried to sample from all regions, or you sampled the most common species, or the most dominant, etc. More detail on the rationale of taxa sampling are needed here.

>> We indeed sampled ALL angiosperm species within the sampled belt, except for difficult apomictic groups, neophytes and water plants. The according text in the MM section was slightly modified and should be clear now (line 381).

376-377: In my experience it is not always so easy to distinguish altered habitats; if you used any specific criteria please mention them here, or if you relied on expert opinion, mention that instead.

>> We wrote "clearly anthropogenic habitats such as ruderal and segetal places as well as strongly altered secondary grasslands were avoided" (lines 382–384). Several of the authors also have a background in vegetation ecology and we are confident that we were able to identify such habitats in the field. Importantly, we do not mean mildly grazed habitats (which are very frequent in the Eastern Alps), but strongly altered ones, which are also identifiable based on their divergent species pool.

394: How did you determine it was reliable? Age of record? Method? How consistent with successive counts it is? Add briefly some detail

>> What we meant is that – following common practice – we excluded chromosome counts (usually (very) old ones) if they deviated from the majority of other counts in other aspects than ploidy (i.e. in untypical base chromosome numbers). This is a practical approach that was followed by essentially all studies comparable to ours as well as in the used data sources such as the account by Dobes & Vitek (2000). The according text in the MM section was modified and should be clear now (lines 398–400).

402: Why were this excluded? I understand the rarity part, and odd-ploidy levels are presumably rare in your dataset; did you have any case of common odd ploidy levels (outside of apomictic genera that you excluded already)?

>> Rare odd ploidy cytotypes were excluded because they typically represent sporadic cytotypes rather than stable, established lineages with long-term persistence. As a result, they are generally rare and not representative of the main cytotype structure within populations. In our dataset, odd-ploidy levels were mostly rare, but in some cases (most of them apomicts from outside the excluded genera / species groups *Alchemilla*, *Hieracium* and *Rubus* sect. *Rubus*) the dominant cytotype was odd, such as in *Calamagrostis villosa*, *Cotoneaster tomentosus*, *Potentilla crantzii*, *Sorbus chamaemespilus*, *Viola sudetica* subsp. *lutea*.

451-452: Is this right? Why polyploid frequency / number of taxa?
Phrased like this seems like if you have e.g. 10 polyploid samples in a pool of 50 species, this variable would be the same value whether the polyploids samples are from 10 different species or all from the same species! What are you looking at with this variable?

>> See reply above (Polyploid frequency)

505-507: Why? Too few samples at that elevation? This really needs a justification

>> The belt N was sampled in order to include the regional pool of cold-adapted species on low mountains not reaching into the 5th belt. For analyses involving elevation, we excluded samples from the N belt since these are not part of the continuous elevational gradient in relation to the timberline, which was used as a reference. The samples from belt N were collected outside this gradient and are not directly comparable in terms of elevation relative to timberline. Including them

would confound the elevational analysis, as these points do not represent the same ecological gradient. In contrast, for analyses involving distance from refugia, all samples were included to better represent the full species pool. The according text in the MM section was slightly modified and should be clear now (lines 509–510).

**Review of NCOMMS-26-007639**

Polyploid frequency decreases with elevation but increases with distance to glacial refugia in the Eastern European Alps, by Zeni et al.

**Overall**

The manuscript reports novel and important results based on extensive and thorough design of georeferenced and habitat-based sampling, appropriate statistical modelling and analyses. Main results, as reflected in the title, are (1) ploidy frequency decreases with elevation but (2) increases with distance to glacial refugia in the Eastern European alps. About these two main points, the authors explicitly describe all relevant sections clearly, from background, methodology to results and discussion. But, the third topic, that is "disentangling the effects of elevation and distance from refugia" is more complex and needs some adjustment, such as bringing point-conclusions up front. This third topic includes the analysis of interaction between them (elevation and distance) when accounting for potential effects of taxonomic composition, when sectioning across mixed-ploidy taxa, and when including functional traits, such as plant dispersal strategy and light requirement that may affect the relationship. The relationships are clearly complex, but most interesting.

**Ploidy frequency decreases with elevation**

Abstract, page 1, lines 21-23. The text reads "*Based on these data, we find that polyploid frequency decreases with elevation, <u>in contrast to expectations</u> derived from global latitudinal distribution patterns*."

Discussion, page 14, lines 352-355. The text reads "*Our results <u>clearly reject the hypothesis</u> that ploidy level shall increase with elevation, challenging at least the generality of the notion on the positive association of polyploidy with harsh and unpredictable environments*."

This conclusion has an unfairly negative connotation. Please consider rephrasing. The two scenarios should be compared subjectively, not putting one against another, i.e. as expected and the other as observed. When the observed does not agree with the expected, the expected is rejected. The two scenarios are completely different.

The polyploid frequency by elevation in the present study is a "regional" pattern and therefore should not be compared directly in a yes or no situation with the "global" latitudinal distribution patterns revealed by other studies or compilations, such as Rice et al. 2019 (Nat. Ecol. Evol. 3: 265-273), Brochmann et al. 2004 (Biol. J. Linn. Soc. 82: 521-536), Löve & Löve 1975 (Cytotaxonomical Atlas of the Arctic Flora. J. Cramer, Vaduz) and Stebbins 1971 (Chromosomal Evolution in Higher Plants, JW Arrowsmith Ltd, Bristol). There, the latitudinal range is very large, from equatorial tropics and subtropics, through temperate ecozones, to the arctic regions. Although it may be comparable between "arctic" and "alpine" in terms of low temperatures (cold), there are other parameters involved that make them different, e.g. day length. The elevation gradients over a mountain range could fall within a narrower latitudinal range. High latitudes like those in the arctics allow for short growing season, making flowering times of closely related species significantly overlap, promoting hybridisation, and in some cases leading to allopolyploidisation. High altitudes, on the other hand, comprises stable species diversity and taxonomical compositions, thus there is little space that could allow new hybridisation and colonisation. As rightly argued in the present manuscript, new polyploids may

not have a chance to invade the already stable, cold-adapted vegetation that is also tolerant to high altitude stresses. The vegetation of the upper alpine belts is likely to comprise both adapted polyploids and adapted diploids. The present study revealed low polyploid frequency in the upper belts in proportion to diploids, relative to polyploid frequencies at lower altitudes, i.e. below the timberline. But the polyploid frequencies at low altitudes are shown in the present study to have already been enriched due to the movement away from the refugia peripheral to the glaciated sites. The picture is more likely that the (new) polyploidy on the Eastern Alps does not move up the elevation gradients above the timberline. The polyploid frequency "does not increase" further up to the high elevation, rather than it is decreasing with elevation!. Stebbins (1971) mentioned (page 180) that "*the percentage of polyploids in the flora of the high Alps, comprising the rocky refuges for plants which exist above the snow line, is no higher than in the plains and foothills below*".

The arctic and the upper alpine are geographically different. While alpine mountain tops may be irregularly spaced and vegetatively discontinuous, not allowing plant migration without passing through the low elevations, there is a history of relevant connectivity throughout the arctic regions, not just across the latitudes, south-north, and also in the east-west direction (Walker et al. 2016. Circumpolar Arctic vegetation. Environ. Res. Lett. 11 055005). Numerous species have circumpolar distributions. Throughout the cycles of glaciation and deglaciation in the Pleistocene, the arctic landscape changed dramatically, often in the back-and-forth manner. At glacial maximum, the sea level fell, thus opening up more space and more connectivity of the unglaciated areas allowing plant migration, often leading to (allo-) polyploidisation. The Berring Landbridge is an example. Shortening distance between islands is another example. Refugia feeding the arctic were not only in the south of the icesheets but also from the east-west peripheries. Polyploid species did not just derive from diploid ancestors, but previously isolated polyploid species most probably hybridised with one another as well, through secondary contacts, hence climbing up the ploidy ladder. Looking at Svalbard as a model area (Brochmann et al. 2004), most (78.3%) of the 161 native taxa are polyploid, and the average ploidal level based on all species, including diploids, is close to hexaploid (5.4), and many species have very high ploidal levels. Further west into the Atlantic, we see polyploid-rich Icelandic flora (Löve & Löve 1956. A cytotaxonomical conspectus of the Icelandic flora. Acta Horti Gothobergensis 20: 65–290.), which is (most likely all) post-glacial, mostly coming in from northern Europe. Overall in the panarctic, the proportion of diploids versus polyploids is 13% vs. 87% in the glaciated region 'Atlantic' (including Svalbard and Iceland), whereas in the non-glaciated region 'Beringian' (including Siberia, Alaska and Yukon) the proportion is 31% vs. 69% (Brochmann et al. 2004). Estimated percentages of polyploidy for north-western Alaska (largely unglaciated) are 56-59% (Stebbins 1971).

**Ploidy frequency is positively associated with distance from glacial refugia**

The present study, based on the entire dataset of ca. 42,000 samples, revealed positive correlation between ploidy frequency and distance from glacial refugia, and across all transects. The further away from refugia the higher polyploid frequencies. This complies with other studies on this aspect, such as Brochmann et al. 2004. As suggested in this present paper, the results are consistent with the view that polyploids are more successful colonizers following deglaciation.

However, when looking into the variation components, the frequency of polyploids was found to vary significantly across the three geographical subunits of the Eastern Alps. Mean frequency was higher in the Central Eastern Alps surrounding most of the previously glaciated area, than in

the limestone areas covering the peripheral refugia in the north and the south. This is thought to be due to refugial persistence of diploid linages and differential postglacial expansion of polyploids into newly deglaciated terrains, whereby poor nutrients in the limestone calcareous bedrock (in the refugia at low elevations further away from the main glaciated area) presumably restricting the abundance of polyploids. If I understand it correctly. The authors further concluded that this is because polyploids are probably not adapted to marginal environments, in contrast to the general knowledge. Another question here.

Nevertheless, the authors stated that differences in taxonomic composition of the angiosperm floras across these three regions cannot be excluded as contributing to the observed spatial variation in polyploid frequency. In fact, the present study already showed different directions of polyploid frequencies among different taxonomic groups, be that by species or by genera, regarding either elevation or distance from refugia (Supplementary Figures 6-9). Some genera (or species) showed increasing polyploid frequency with elevation, some not increasing and others displayed no change, and this is the same for the distance from refugia. Supplementary Figure 11 shows examples of four different geographic distribution of diploid vs. tetraploid cytotypes (in mixed-ploidy species) across the Eastern Alps: (a) *Biscutella laevigata* with diploids restricted to peripheral refugial areas, (b) *Bartsia alpina* with tetraploids restricted to peripheral refugial areas, (c) *Briza media* and (d) *Juncus trifidus* with no strict refugial association but rather longitudinal separation. Clearly, taxonomic composition of the angiosperm floras across these different regions of the eastern Alps cannot be discarded as not contributing to the overall spatial variation in polyploid frequency.

**Disentangling the effects of elevation and distance from refugia**

The statistical modelling in the present study demonstrated a significant interaction between the effects of distance from refugia and elevation. This is to not surprising. The study region is a mountain range, therefore distance from refugia is mostly in the direction up the elevation. The main point in this study is the discovery that polyploid frequency increases away from the peripheral refugia (low elevation presumably) up to around the timberline and does not seem to make it higher up to the upper alpine zones. This is interesting. The study design included a fine-scale sampling not reported before. This opens up many biological questions to be answered by utilizing this very large dataset further. The reasons for polyploid frequency not increasing more to the upper zones can be many, some of which have been discussed in the present paper.

In the present study, certain plant functional traits were examined. In one scenario, the dataset was classified by plant dispersal ability (good vs. poor) and light preference (light-demanding vs. shade tolerance).

The results on page 7, lines 167-170: "*The same elevational and spatial patterns as for the entire data set were revealed for poor dispersers (Supplementary Fig. 2), whereas no significant trend was found for good dispersers (Supplementary Fig. 3)*". The authors suggested that the dispersal mode modified the relationship between ploidy and elevational gradient, likely because efficient migrators maintained higher connectivity among populations during glacial cycles preventing the establishment of newly emerging polyploids at the leading edge of a species' distribution. But when looking at the figures, the reason why no significant trend was found among good dispersers, statistically, is because the variation at the upper belts is so large and covering as much negative as positive trends. Therefore, the explanation given in the present study may not be the best. The good (fast) dispersers must be highly variable, for example, due to different means of dispersal among different plant taxonomic groups. Long-distance

dispersal, such as by wind like in *Betula* that produces numerous, light winged seeds, can maintain good connectivity among populations as suggested. On the other hand, *Salix*, which comprises pioneer or early succession species well-adapted to disturbance, is mostly clonal (asexual). More than half of species in this genus is polyploid and the genus comprises numerous (newly formed) hybrids (Kaplan et al. 2019. Key to the flora of the Czech Republic, ISBN: 978-80-200-2660-6). Supplementary Figure 6 shows significantly positive relationship between polyploid frequency and elevation in *Salix* (page 14), the reverse of the main trend deriving from the whole dataset.

When classifying species according to their light preference, clear difference emerged between shade-tolerant and light-demanding plants. Light-demanding taxa exhibited the same pattern as that across the entire dataset, regarding both distance and elevation. Among shade tolerant species, the distance-from-refugia effect persisted while the elevation effect was reversed (Supplementary Figures 4 and 5). Here the authors speculated (on page 12, lines 301-304) that "*colonisation dynamics in forest species (mostly shade-tolerant) were more dynamic and hence more influential than in alpine species – which are predominantly light-demanding – as lower elevation species likely experienced stronger fragmentation during cold stages and longer periods of vicariance in smaller and fewer refugia*." The authors suggested that the results here pointed to a stronger sorting of established polyploids vs. diploids in refugia in the course of postglacial colonisation dynamics. But when looking closely at supplementary Figure 4, the "reverse elevation effect" among shade-tolerant species is just at the border of statistical significance with $p=0.05$. Although the mean frequency of polyploid at the upper-most belt is higher than from lower belts, the variation at the uppermost belt is huge, and there, it was based on low number of shade-tolerant polyploid species. Visually, the pattern of polyploid frequency by elevation among shade-tolerant species is not solidly reverse as concluded. The polyploid frequency from both shade-tolerant and light-demanding species appears not to have increased further up above the timberline.

The **conclusion** stated on page 14, lines 344-346, is most appropriate and fair, that is "*Our study bridges the gap between many single-species case studies, which yielded contradictory results on the one hand, and global studies addressing species-level patterns at coarser scales on the other hand.*"
